# *ANO5* mutations in the Polish limb girdle muscular dystrophy patients: Effects on the protein structure

**DOI:** 10.1038/s41598-019-47849-3

**Published:** 2019-08-08

**Authors:** Adam Jarmula, Anna Łusakowska, Jakub P. Fichna, Malgorzata Topolewska, Anna Macias, Katherine Johnson, Ana Töpf, Volker Straub, Edyta Rosiak, Krzysztof Szczepaniak, Stanisław Dunin-Horkawicz, Aleksandra Maruszak, Anna M. Kaminska, Maria Jolanta Redowicz

**Affiliations:** 1 Laboratory of Bioinformatics, Nencki Institute of Experimental Biology, Polish Academy of Academy of Sciences, 3 Pasteur St., 02-093 Warsaw, Poland; 2 Department of Neurology, Medical University of Warsaw, 1a Banacha St., 02-097 Warsaw, Poland; 3 Department of Neurodegenerative Disorders, Mossakowski Medical Research Centre, Polish Academy of Sciences, 5 Pawinskiego St., 02-106 Warsaw, Poland; 4 Laboratory of Molecular Basis of Cell Motility, Nencki Institute of Experimental Biology, Polish Academy of Sciences, 3 Pasteur St., 02-093 Warsaw, Poland; 5 John Walton Muscular Dystrophy Research Centre, Institute of Genetic Medicine, Newcastle University, Newcastle-upon-Tyne, United Kingdom; 6 2nd Department of Radiology, Medical University of Warsaw, 1a Banacha St., 02-097 Warsaw, Poland; 7 Laboratory of Structural Bioinformatics, Centre of New Technologies, University of Warsaw, 2c Banacha St., 02-097 Warsaw, Poland

**Keywords:** Genetics research, Computational biology and bioinformatics, Neuromuscular disease, Medical genomics

## Abstract

LGMD2L is a subtype of limb-girdle muscular dystrophy (LGMD), caused by recessive mutations in *ANO5*, encoding anoctamin-5 (ANO5). We present the analysis of five patients with skeletal muscle weakness for whom heterozygous mutations within *ANO5* were identified by whole exome sequencing (WES). Patients varied in the age of the disease onset (from 22 to 38 years) and severity of the morphological and clinical phenotypes. Out of the nine detected mutations one was novel (missense p.Lys132Met, accompanied by p.His841Asp) and one was not yet characterized in the literature (nonsense, p.Trp401Ter, accompanied by p.Asp81Gly). The p.Asp81Gly mutation was also identified in another patient carrying a p.Arg758Cys mutation as well. Also, a c.191dupA frameshift (p.Asn64LysfsTer15), the first described and common mutation was identified. Mutations were predicted by *in silico* tools to have damaging effects and are likely pathogenic according to criteria of the American College of Medical Genetics and Genomics (ACMG). Indeed, molecular modeling of mutations revealed substantial changes in ANO5 conformation that could affect the protein structure and function. In addition, variants in other genes associated with muscle pathology were identified, possibly affecting the disease progress. The presented data indicate that the identified *ANO5* mutations contribute to the observed muscle pathology and broaden the genetic spectrum of LGMD myopathies.

## Introduction

Limb-girdle muscular dystrophies (LGMD) form a group of inherited myopathies showing progressive limb-girdle weakness with sparing of the facial and distal muscles. According to the inheritance mode, LGMD is classified into two general categories: autosomal dominant (LGMD1) and autosomal recessive (LGMD2) forms. So far, 33 genes have been associated with the disease, nine dominant and 26 recessive^1–3^. Molecular pathophysiology and therefore clinical phenotypes of the LGMDs are highly heterogeneous as the mechanisms of disease include defects in numerous proteins^1,4^. The observation that mutations within the same gene could be associated with variable phenotypes was made for numerous LGMD associated genes, including desmin and dysferlin^1,4–8^. It is noteworthy that variable symptoms are often observed even within a family with the same causative mutation, indicating that a primary pathogenic variant combined with potentially modifying variants may exacerbate or mitigate the phenotype (for example^9^).

The LGMD2L subtype is associated with mutations in the anoctamin-5 gene (*ANO5*). *ANO5* is localized to chr11p14.3, spans 90,192 bp and contains 22 exons. The gene encodes anoctamin-5 (ANO5), a ~100-kDa protein composed of 913 amino acids, containing eight transmembrane domains^10,11^. The protein belongs to a family of at least ten proteins (ANO1-10) involved in a variety of cellular functions such as ion transport, phospholipid scrambling, and regulation of other membrane proteins^12,13^. While *ANO1*, *ANO2*, *ANO6*, *ANO8*, and *ANO9* were found to code for calcium-activated chloride channels (CaCC), an exact function of *ANO5* still remains elusive. Currently ANO5 is believed to be engaged in processes related to muscle membrane repair and ion transport^12,13^. ANO5 localizes to the endoplasmic reticulum (ER) where it is postulated to function as an intracellular channel^14^. In addition, *ANO5* is highly expressed in cardiac and skeletal muscle as well as in chondrocytes and osteoblasts, suggesting its important role(s) in the musculoskeletal system^15^.

The still-expanding spectrum of *ANO5*-related clinical phenotypes is highly variable. Although the majority of known mutations are recessive and cause muscle disease, rare autosomal dominant *ANO5* mutations cause a totally different clinical entity, gnathodiaphyseal dysplasia (GDD)^14–16^. Moreover, the recent study linked dental tumor (giant cementoma) to an autosomal dominant mutation in *ANO5* associated with gnathodiaphyseal dysplasia^17^. The functional differences between dominant and recessive *ANO5* mutations are not yet fully understood although there is a report indicating that homodimer formation or interaction with other anoctamin isoforms could be considered^17^. The muscle phenotype ranges from asymptomatic hyperCKemia and exercise-induced myalgia to proximal and/or distal weakness. The most typical presentation is LGMD2L, diagnosed world-wide and is one of the most frequent in Northern Europe encompassing 10–20% of LGMD cases^6,9,18^. Miyoshi-like disease or Miyoshi muscular dystrophy 3 (MMD3), also caused by recessive mutations in *ANO5*, is less common^19^.

LGMD2L is characterized generally by asymmetric muscle involvement with prevalent quadriceps, biceps brachii atrophy, and pain following exercise. The disease onset is in adulthood and the patients are usually ambulant^6^. There are numerous mutations identified within ANO5 and many of them are localized to the exons coding for the cytoplasmically-localized domains, predominantly at the 299 amino acid-long N-terminal segment^9,20^. The first described and most common mutation described so far is c.191dupA (p.Asn64LysfsTer15) in exon 5, most probably due to a founder effect^9,17,21,22^.

In the study presented herein, we address the mechanisms of clinical variability of *ANO5*-associated muscular dystrophies. Thorough analyses of the clinical data of five patients with heterozygous mutations within *ANO*5 and variable phenotypes were performed. Variants identified in two of these patients were already reported in our previous study^23^ without addressing the consequences of mutations on the protein structure and clinical phenotype. Out of nine identified mutations, three were not reported in databases, p.Lys132Met and p.Trp401Ter, and one, p.Tyr23Ter, was only listed in the ExAC database. None of the them was described in literature. Clinical evaluation of the patients (including MRI imaging) and morphological examination of biopsied muscles were aided by the molecular dynamics-based structural analysis of the effects of missense mutations on the structure of ANO5 dimer, the model of which was created for the purpose of this study. Our results indicate that the location of the mutation within the polypeptide chain affects the protein structure not only locally, in the vicinity of the mutated residue, but also globally, influencing the dimer interface and dynamics, and seemingly the function.

## Results

Comprehensive analyses of five patients admitted to the Department of Neurology of the Medical University of Warsaw with symptoms of LGMD were carried out; mutations in *ANO5* were subsequently identified for each of them. The analyses included clinical and electrophysiological evaluation, morphological examination of biopsied muscles, MRI of muscles, and genetic analyses including whole exome sequencing (WES). In addition, extensive molecular modeling analyses were performed in order to evaluate the potential effects of the identified mutations on the ANO5 dimer structure and hence function.

### Clinical evaluation

A clinical summary of the examined patients (numbered consecutively 1–5) is presented in Table 1. Pedigrees of the probands’ families as well as detailed case descriptions are presented in Supplementary Material 1 (parts I and II, respectively). Except for patient 1, family members of other patients were not affected.

**Table 1:** Summary of patients’ clinical history.

Patient	Gender	Family history	Age at onset/biopsy in yrs	First symptoms	Clinical phenotype	Asymmetry/location	CK range	EMG + ENG	Age of last examination
1	M	Reportedly older brother had similar symptoms	Third decade/54	Gait disturbances, myalgia	Progressive muscle weakness with limb-girdle distribution, most pronounced in posterior thigh muscles. Two episodes of cardiac insufficiency. Alcoholism in anamnesi	+/upper limbs	19x	Myogenic in proximal muscles, mixed in distal muscles. ENG-motor-sensory, axonal-demyelinating neuropathy	Died in 2016 COD unknown
2	M	Sporadic	22/22	Myalgia, exercise intolerance	Mild generalized hypertrophy, right scapular winging	+/calves	20–50x	Mild myopathic changes, ENG normal	30
3	M	Sporadic	Third decade/36	Episodic myalgia and muscle cramps	Mild generalized muscle hypertrophy	+/calves and qadriceps	10–15x	Normal	36
4	F	Sporadic	38/38	Myalgia, gait disturbances	Slowly progressing mild weakness of upper and lower extremities	+/calves	3–8x	Normal	41
5	F	Sporadic	25/42	Weakness of the right lower limb	Slowly progressing weakness of lower and upper limbs-with waddling gait and calf muscles involvement	+/lower and upper limbs	9–20x	Myopathic in upper limbs, normal in VL	42

M, male, F, female, CK-creatine kinase, EMG-electromyography, ENG-electroneurography, COD- cause of death, VL-vastus lateralis.

Clinical characteristics of all examined patients fulfilled the criteria of limb-girdle muscular dystrophy. All patients presented with mild, slowly progressive muscles weakness and/or atrophy, more prominent in the proximal muscles of the lower legs. High creatine kinase (CK) levels (up to 50 times the upper limit), asymmetric weakness and myalgia were observed in all cases. The age of onset was in the third or fourth decade, however, the first symptoms such as gait disturbances, myalgia, muscle cramps, exercise intolerance or asymmetric weakness (of one leg) were variable.

Patients 2 and 3 (males) presented with mild generalized muscle hypertrophy. In two cases (patients 1 and 5), the posterior compartment of the lower leg muscles was substantially involved. On EMG, myopathic changes were detected, however, in two patients (3 and 4) EMG was normal.

### Magnetic Resonance Imaging (MRI)

**Figure 1:**
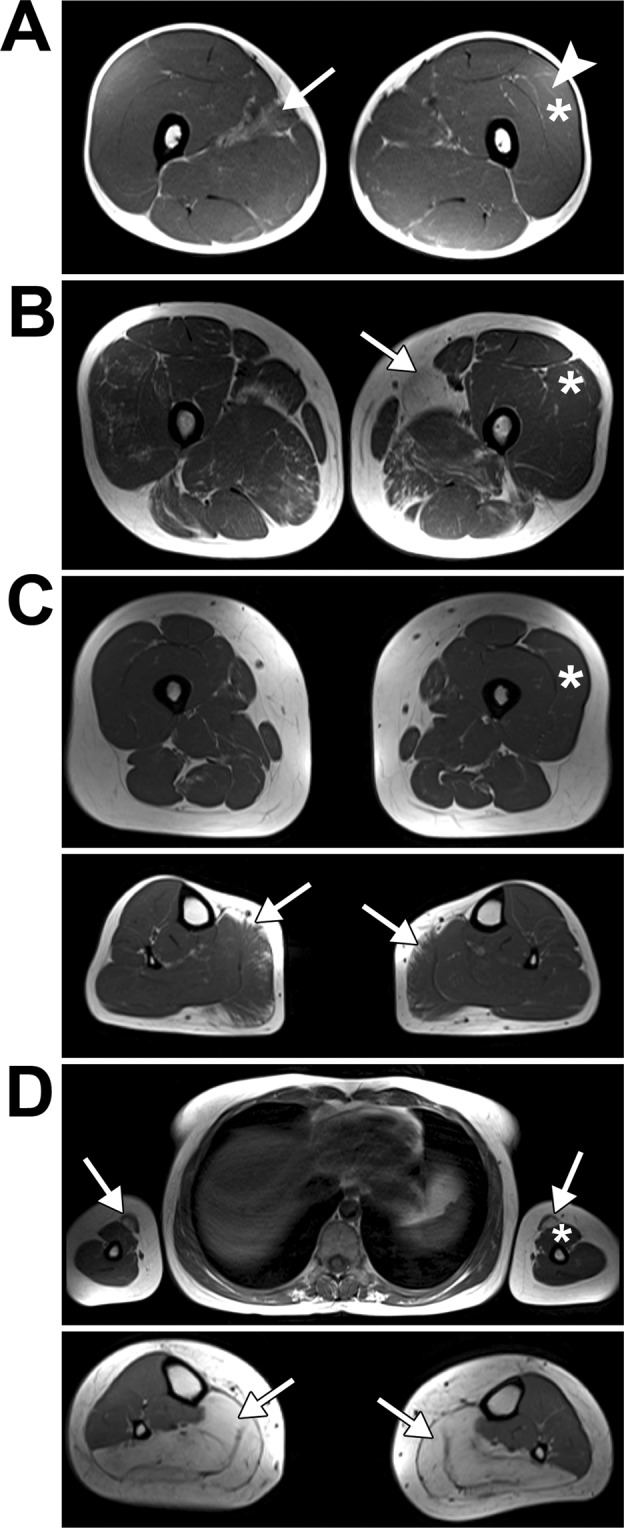
Magnetic Resonance Imaging (MRI). (**A**–**D**) Selected axial T1W images of muscles of patients 2–5. (**A**) Images of patient 2 thigh muscles - arrow points to fatty atrophy in right adductor longus muscle; asterisk marks biopsied left vastus lateralis muscle with a mild fatty degeneration (arrowhead). (**B**) Images of patient 3 thigh muscles - arrow points to severe fatty atrophy in left adductor longus muscle; asterisk marks biopsied left vastus lateralis muscle. (**C**) Images of patient 4 muscles; upper panel - thigh muscles with biopsied left vastus lateralis muscle (asterisk); lower panel - arrows point to fatty atrophy in medial head gastrocnemius muscles. (**D**) Images of patient 5 muscles; upper panel - arrows point to fatty infiltrations in biceps brachii muscles, asterisk marks biopsied left biceps brachii muscle; lower panel - arrows point to severe bilateral fatty infiltration of the soleus and gastrocnemius muscles.

MRI examinations were performed for patients 2–5 and are presented in Fig. 1 and Supplementary Material 2. Patient 1 is deceased and thus could not be examined (see Table 1).

The analysis revealed that patient 2 presented with very severe, symmetric atrophy of the medial head of gastrocnemius muscle as opposed to other patients who showed muscle fatty replacement with little volume loss or even relative hypertrophy (for details see Supplementary Material 2). Asymmetric changes and fat infiltration were visible in the adductor longus muscle at the thigh level (Fig. 1A, arrow), similar to patient 3 (see Fig. 1B). However, there was no decrease in muscle bulk. The biopsied left vastus lateralis muscle showed only mild fatty degeneration (Fig. 1A, asterisk and arrowhead; and Supplementary Material 2).

In patient 3, muscle fatty degeneration changes in lumbar paraspinal muscles and lower limbs muscles with little or no change in the muscle bulk were seen (for details see Supplementary Material 2). At the pelvic level, the gluteus minimus muscle was bilaterally severely affected, more than the gluteus medius and maximus muscles. Asymmetric muscle changes and fatty degeneration were visible at the thigh level in the adductor longus muscle and all heads of the vastus muscle (Fig. 1B, arrow). Mild to moderate symmetric changes were observed in the adductor magnus muscle and hamstrings. In the lower legs, diffuse and severe changes were present in the medial head of gastrocnemius muscle and less severe in the lateral head of the gastrocnemius muscle with relative sparing of other muscles (Supplementary Material 2). The biopsied left vastus lateralis muscle (Fig. 1B, asteriks) showed only very mild fatty changes.

Patient 4 presented with mild fatty degeneration changes in the following muscles of the lower extremities: the gluteus muscles, the adductor magnus muscle and hamstrings (see Supplementary Material 2). Moderate fatty degeneration were visible in the medial head of the gastrocnemius muscle (Fig. 1C, lower panel, arrows). Also, MRI showed little decrease in the volume of the affected muscles. The biopsied left vastus lateralis muscle showed no fatty degeneration (Fig. 1C, upper panel, asterisk).

Patient 5 was the only patient in this study who showed abnormalities in the upper limbs with severe fatty degeneration and atrophy of the biopsied left biceps brachii muscle (Fig. 1D, upper panel, asterisk and arrow, and Supplementary Material 2). Changes in paraspinal muscles were more severe than those in patient 3. At the thigh level, the gluteus minimus and medius, adductor magnus and hamstrings were bilaterally severely affected with decreased muscle bulk (Supplementary Material 2). Also, some fatty degeneration changes were seen in the vastus muscles, similarly to those in patient 3 (Supplementary Material 2). In the lower legs, the patient presented with severe or complete fatty infiltration and relative hypertrophy of the medial head of the gastrocnemius muscle (Fig. 1D, lower panel, arrows), the soleus muscle, and the flexor hallucis longus muscle (Supplementary Material 2).

Moreover, in all the patients edema-like changes of the affected muscles (being a hallmark of ongoing disease activity in the muscles^24^), manifesting as increased signal in STIR (Short Tau Inversion Recovery) sequences,were seen (Supplementary Material 2).

### Morphological analysis of biopsied muscles

**Figure 2:**
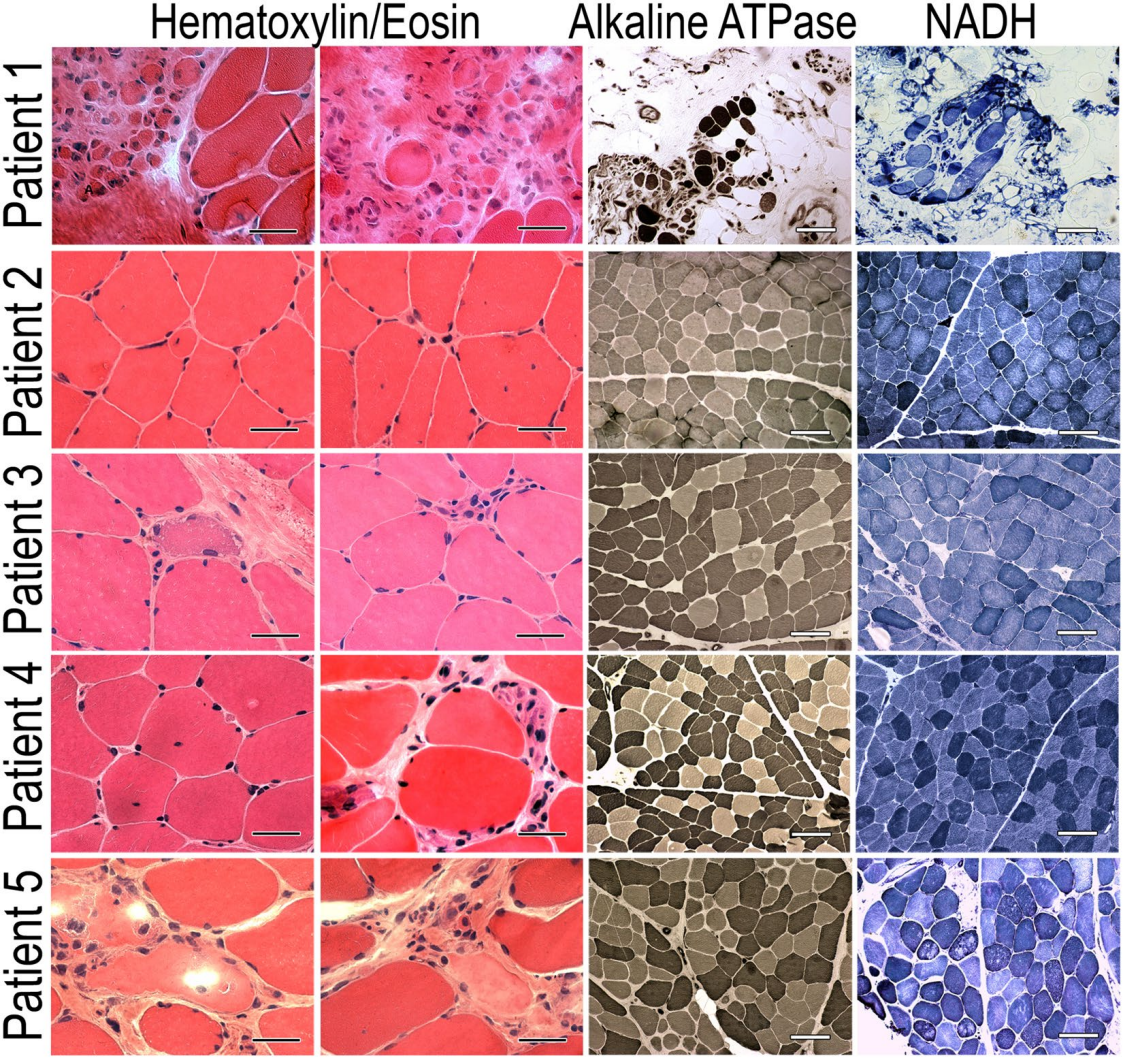
Analysis of morphology of the patients’ muscles. The panels present routine stainings with hematoxylin/eosin (bars 50 μm) as well as for the alkaline ATPase and NADH activities (bars 150 μm).

Hematoxylin/*eosin (HE) stain*ing of muscle transverse sections of the patients’ biopsies revealed variable primary myopathic changes (Fig. 2 and Table 2). The changes were either mild as in the case of patient 2 or very severe as in the case of patient 1 (Fig. 2). Variability in the fiber size was observed in all the biopsied muscles and centrally positioned myonuclei were visible as well. Also, variable changes in stainings for the alkaline ATPase and NADH activities were visible, indicating a different state of muscle degeneration of biopsied muscle. The summary of the described morphological changes is presented in Table 2.

**Table 2:** Morphological analysis of muscle biopsy.

	Age at biopsy	Biopsied muscle	Atrophy	Hypertrophy	Splitting	Necrosis	Phago-cytosis	Regeneration	Internal nuclei	Endomysial infiltrations	Endomysial connective tissue	Endomysial fatty tissue	General conclusion
Patient 1	54	BB	+++	−	−	+	−	+	+	+	+++	+++	Advanced dystrophic changes
Patient 2	22	VL	+	+	+	−	−	+	++	−	+	−	Discrete myopathic changes suggesting DM2
Patient 3	36	VL	+	+	+	−	−	+	++	−	−	−	Moderate uncharacteristic myopathic changes
Patient 4	38	VL	++	+	+	−	−	−	+	+	+	+	Moderate uncharacteristic myopathic changes.
Patient 5	42	BB	++	++	+	++	++	++	+	+	+	+	Advanced dystrophic changes suggesting dysferlinopathy

BB, *biceps brachii*; VL, *vastus lateralis*; −, not observed; +, minimal; ++, moderate; +++, advanced; DM2, myotonic dystrophy type 2.

### Genetic analyses

**Figure 3:**
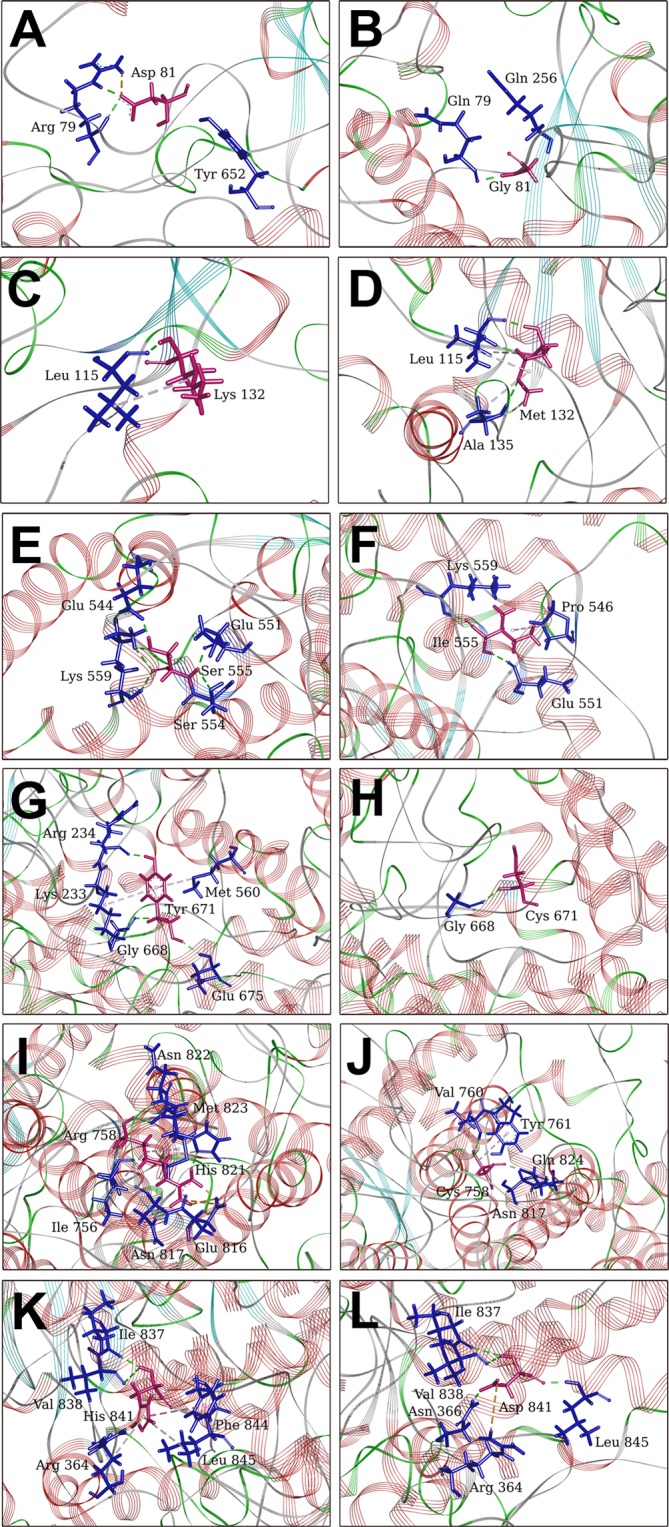
Local effects of mutations on ANO5 structure. Most occupied hydrogen bonds and hydrophobic interactions (green and light purple dashed lines, respectively) for the wild-type (left panels) and mutant sites (right panels) at positions: p.Asp81Gly (**A**,**B**), p.Lys132Met (**C**,**D**), p.Ser555Ile (**E**,**F**), p.Tyr671Cys (**G**,**H**), p.Arg758Cys (**I**,**J**) and p.His841Asp (**K**,**L**). The mutated residues at positions 81, 132, 555, 671, 758 and 841 are shown in magenta, while all residues interacting with them are shown in blue. All remaining parts of the protein are shown as line ribbons and colored according to secondary structure assignment (α-helices in brown and β structures in green). Hydrogen bonds occurring in the trajectory but not in the average structures lack the dashed lines. Their appearance in the figure consists solely in the presence of both residue 81, 132, 555, 671, 758 or 841 (magenta) and its interacting partner (blue).

**Figure 4:**
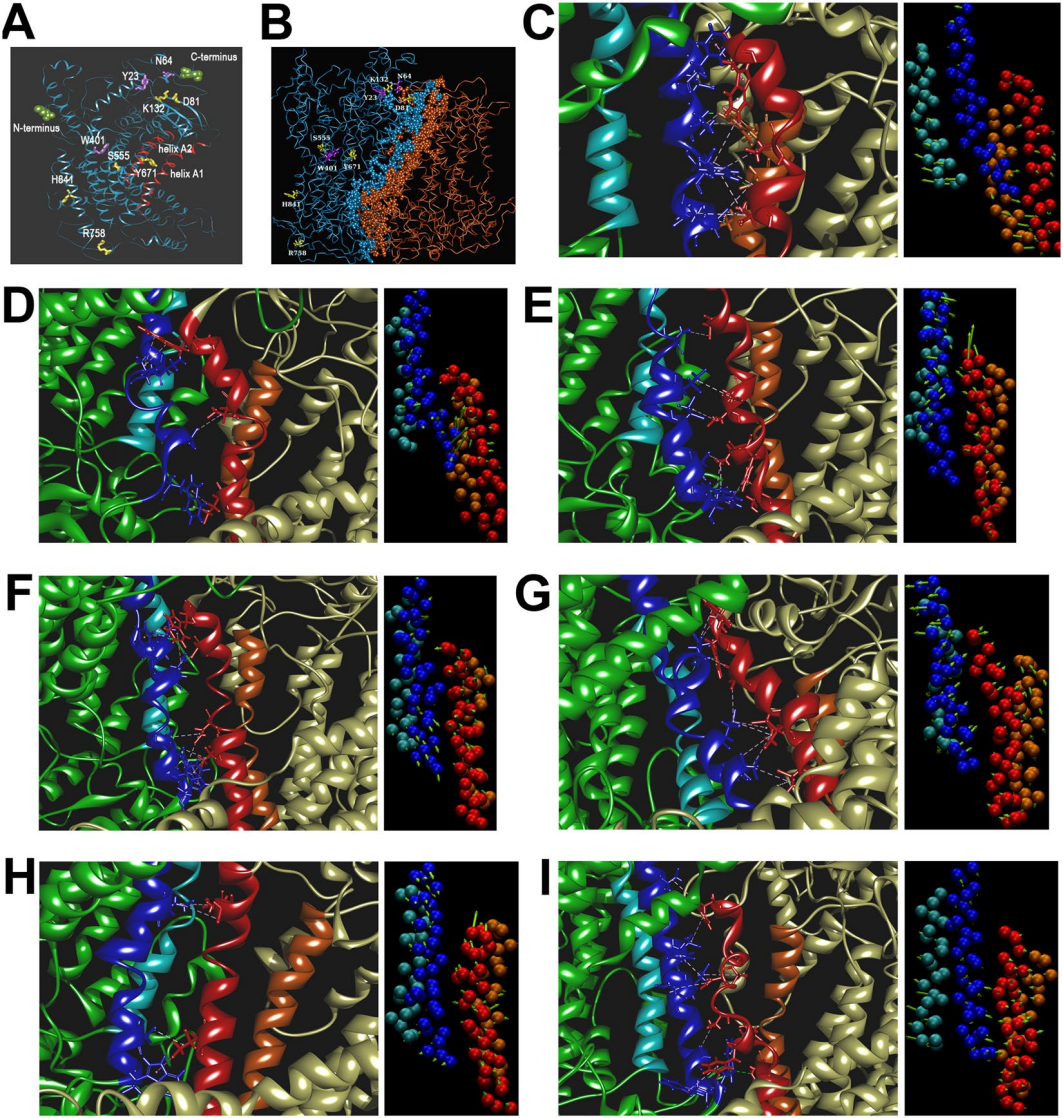
Global effects of mutations on ANO5 structure. (**A**,**B**) Models of ANO5 monomer and dimer, respectively. Residues undergoing missense and nonsense mutations are shown as yellow and purple sticks, respectively. (**A**) Flat ribbon representation of the ANO5 monomer. For space limitation, the residues are presented in the single-letter code. C- and N-termini are shown as olive CPK spheres, respectively. The α-helices A1 (residues 609–639) and A2 (residues 692–715) are shown in red. Cytoplasmic and extracellular domains are facing the top and bottom of the figure, respectively. (**B**) Model of ANO5 dimer in the tube representation with location of the mutated residues. Subunits A and B are colored in blue and orange, respectively, and residues forming dimerization interface are shown as spheres and color-coded as above. For space limitation, the residues are presented in the single-letter code. (**C**–**I**) Effects of mutations on the dimer interface. Left panels, close view of the central α-helices at the dimerization interface of the wild-type (**C**) and mutants (**D**–**I**) ANO5 structures. (**D**) p.Asp81Gly, (**E**) p.Lys132Met, (**F**) p.Ser555Ile, (**G**) p.Tyr671Cys, (**H**) p.Arg758C, (**I**) p.His841Asp. Helix A1, blue; helix A2, cyan, helix B1, red; and helix B2, orange. Subunits A and B are colored in green and straw, respectively. Hydrophobic interactions and hydrogen bonds between helices from subunits A and B are shown as light violet and green dashed lines, respectively. Right panels, concerted motions associated with the first eigenvectors (PC1) in PCA-2 for the four central α-helices: A1 (blue), A2 (cyan), B1 (red) and B2 (orange). The actual frames represent the average structures from the first mode of motion. Residues forming the helices are shown as C-α van der Waals spheres. Motions are represented by green arrows indicating the direction of motion and its amplitude.

WES identified on average 66,873 genetic variants per patient, of which 45,156 off-target (defined as intergenic or intronic, but not affecting splice sites) were removed from further consideration. In total variants in 1,153 genes were further analyzed. In every filtering/prioritization approach mutations in *ANO5* were ranked first on the results lists. The identified changes are rare, with a minor allele frequency below 0.1% (see Table 3). According to our ANO5 model that has been created for the purpose of this study, Tyr23, Asn64, p.Asp81 and Lys132 residues are situated within the cytoplasmic domains, Arg758 is facing the extracellular matrix, while the other residues (Trp401, Ser555, Tyr 671 and His841) are located in the central part of the molecule, mostly within the transmembrane α-helices (Figs 3 and 4). The variants were deemed pathogenic by at least one prediction program: Mutation Taster, Polyphen2 PROVEAN or SIFT, and were absent in our in-house database of 110 Polish exomes/genomes (see Table 3)^25–28^. In addition, His841Asp was predicted by dbscSNV as potentially altering splicing with both its adaptive boosting (ada_score) and random forest (rf_score) scores larger than 0.6^29^. All these variants were individually assessed by a board of geneticists and clinicians according to the guidelines of the American College of Medical Genetics and Genomics^30^. Also, several other accompanying mutations in muscle genes have been identified. Unfortunately, segregation analysis could not be performed because of the unavailability of the DNA samples of the relatives. A detailed list of accompanying mutations for patients 1 and 2 was presented in^23^, and for patients 3–5 is presented in Supplementary Material 3.

**Table 3:** Heterozygous mutations identified in *ANO5* in Polish patients.

	cDNA ENST00000324559,8 NM_213599.2	Protein ENSP00000315371.8 NP_998764.1	Type	Effect on protein PolyPhen2/SIFT	Location GRCh38	Frequency in Exac database	ACMG evidence of pathogenicity	Accompanied heterozygous mutations with a predicted damaging effect*
Patient 1	c.242A>G	p.Asp81Gly	missense	probably damaging/ deleterious	11:22221158	2.25e-04	PS4, PM3, PP3, PP4, PP5	***BAG3***, c.380C>T p.Ser185Leu; 7.63e-5 ***FNLC***, c.2846A>G p.Asp949Gly; 3.89e-04
	*c*.*1203G*>*A*	*p*.*Trp401Ter*	*nonsense*	*stop gained* /*deleterious*	*11:22255393*	*0*	*PVS1*, *PM2*, *PM3*, *PP3*, *PP4*	
Patient 2	c.242A>G	p.Asp81Gly	missense	probably damaging/ deleterious	11:22221158	2.25e-04	PS4, PM3, PP3, PP4, PP5	***NEB*** c.9055G>A p.Ala3019Thr 6.62e-05
	c.2272C>T	p.Arg758Cys	missense	probably damaging/ deleterious	11:22274605	3.30e-05	PS4, PM3, PP3, PP4, PP5	
Patient 3	c.69C>A	p.Tyr23Ter	nonsense	stop gained /deleterious	11:22203832	9.84e-06	PVS1, PM2, PM3, PP4	***RYR1***, 2654G>A p.Arg885His; 1.77e-04
	c.1664G>T	p.Ser555Ile	missense	possibly damaging/ deleterious	11:22262162	7.42e-05	PM3, PP3, PP4	
Patient 4	*c*.*395A*>*T*	*p*.*Lys132Met*	*missense*	*probably damaging*/ *deleterious*	*11:22227333*	*0*	*PM2*, *PM3*, *PP3*, *PP4*	***COL12A1***, c.6752G>A p.Arg2251His; 3.73e-04 ***FNLC***, c.3721C>T p.Arg1241Cys; 1.08e-02 ***LAMA2***, c.2462C>T p.Thr821Met; 2.03e-03
	c.2521C>G	p.His841Asp	missense / splice site	probably damaging/ deleterious	11:22279544	1.67e-05	PM2, PM3, PP3, PP5, PP4	
Patient 5	c.191dupA	p.Asn64LysfsTer15	frameshift, essential splice	frameshift/ deleterious	11:22221107	1.03e-03	PVS1, PM3, PP4, PP5	***AGRN***, c.3353C>A p.Thr1118Lys; 2.15e-03 ***ITGA7***, c.3371G>A p.Arg1124Gln; 4.01e-04 ***ITGA7***, c.2656G>A p.Glu886Lys; 4.01e-03 ***LAMB2***, c.2891G>A p.Arg964Gln; 2.48e-05
	c.2012A>G	p.Tyr671Cys	missense	probably damaging/ deleterious	11:22270425	8.24e-06	PM2, PM3, PP3, PP4	

*With at least one prediction program: Mutation Taster, Polyphen2, PROVEAN or SIFT; *in italic*, mutations not registered in the gnomAD database.

### Molecular modeling

**Figure 5:**
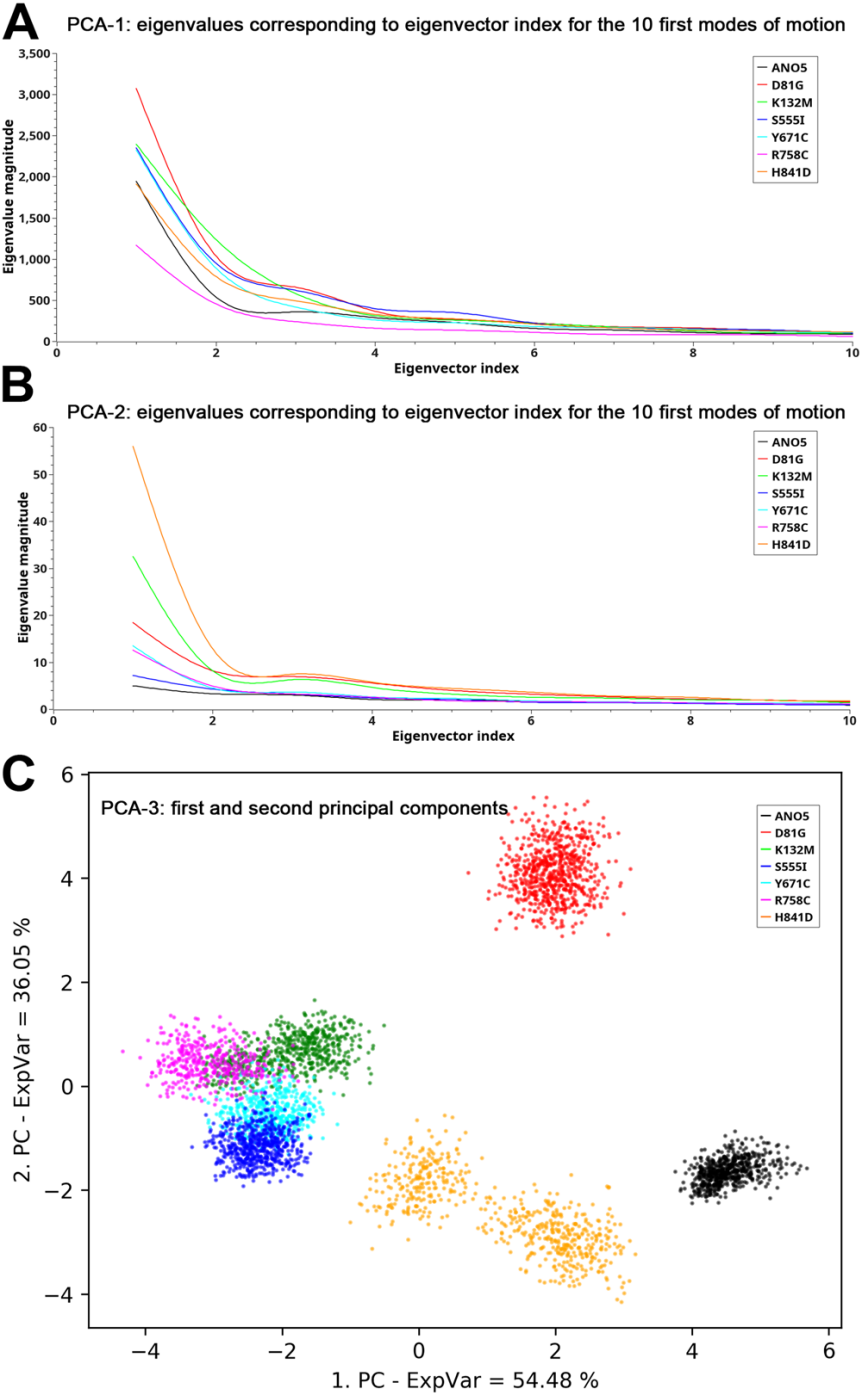
Eigenvector analysis. Plot of eigenvalues corresponding to the eigenvector index for the first 10 modes of motion in the PCA-1 (in (**A**)) or PCA-2 (in (**B**)) analyses carried out for the wild-type and mutant ANO5 dimer structures. **(C)**, time development of projections of 2 features (distance-dependent functions) obtained from PCA-3 analysis of the central α-helices at the dimerization interface in the wild-type and mutant ANO5 structures. Color legend in (**C**) ANO5, black; p.Asp81Gly, red; p.Lys132Met, green; p.Ser555Ile, blue; p.Tyr671Cys, cyan; p.Arg758Cys, magenta; and p.His841Asp, orange.

To predict the impact of identified mutations on the ANO5 structure and hence function, we created a *de novo* model of ANO5 and its dimer (see Materials and Methods section, and Fig. 4A,B). The models were subjected to molecular dynamics (MD) analyses that enabled visualization of both local and global effects of the identified mutations (Figs 3–5 and Supplementary Materials 4–7, and Table 4). The analyses were not performed for the nonsense mutations (p.Trp401Ter, p. Tyr23Ter) and a frameshift p.Asn64LysfsTer15 mutation, which *de facto* also introduced the stop codon.

**Table 4:** Selected parameters from *PISA* evaluation of the dimerization interfaces in ANO5 and its mutants.

Model	Chain A			Chain B			Interface A-B		
	N(at)	N(res)	Surface [Ẳ^2^]	N(at)	N(res)	Surface [Ẳ^2^]	Surface [Ẳ^2^]	ΔG [kcal/mol]	ΔG P-value
ANO5	279	70	42474	307	85	41873	2520.7	−39.7	0.142
p.Asp81Gly	358	94	48767	345	88	48092	3033.2	−36.2	0.661
p.Lys132Met	376	97	47313	381	97	48815	3314.8	−47.0	0.375
p.Ser555Ile	456	121	47717	433	110	49308	3887.3	−46.8	0.713
p.Tyr671Cys	375	105	40483	358	102	39668	3226.0	−48.9	0.202
p.Arg758Cys	360	90	46726	348	91	48777	2977.8	−27.8	0.906
p.His841Asp	320	90	47315	323	92	47046	2844.7	−42.6	0.243

PISA, protein interfaces, surfaces and assemblies service at the European Bioinformatics Institute (http://www.ebi.ac.uk/pdbe/prot_int/pistart.html); N(at) and N(res) indicate the number of interfacing atoms and residues, respectively. ΔG and ΔG P-value indicate the solvation free energy gain and the P-value of the solvation free energy gain, respectively.

#### Local mutation effects

Local effects of the identified mutations were analyzed by investigating the conformational rearrangements around the site of mutation (Fig. 3A–L). To this end, hydrogen bonding and hydrophobic contacts throughout the simulations were accounted. The results indicate that two mutations to cysteine, p.Tyr671Cys and p.Arg758Cys, thoroughly change a role of mutated residues in local interaction networks (Fig. 3G–J). In particular, p.Arg758Cys mutation, the site of which is located on a distant-from-the-interface surface loop 753–822 (Figs 3I,J and 4B), caused the relatively short residue of Cys758 to lose almost entirely several hydrogen contacts with residues from the same loop (especially with Glu816, His821 and Asn822) and with Met823 from a short helical segment 823–825. All of them were maintained by the long and strongly polar residue of arginine 758. Instead, Cys758 maintains a strong H-bond contact with Gln824 and a weak contact with Asn817. In addition, positions of Arg758 and Cys758 are stabilized on a similar level (although more persistently in the case of Arg758) due to hydrophobic contacts with Ile756 and Met823 (to Arg 758) and Val760 and Tyr761 (to Cys758) (Fig. 3I,J, Supplementary Material 4: Tables 8 and 9). Furthermore, there is a weakened stabilization of the mutated residue of Cys671 compared to the wild-type residue of Tyr671 (Fig. 3G,H). This site is located in α-helix 669–684 in the core of each subunit ~15 Å from the dimerization interface (Figs 3G and 4A,B). In the wild-type structure, Tyr671 maintains medium persistent H-bonds with Glu675 from the same α-helix and with Gly668 from a short loop 664–668 as well as strong H-bond with Arg234 from a surrounding loop 222–237 (Fig. 3G). In the p.Tyr671Cys mutant structure, Cys671 loses the H-bonds with Glu675 and Arg234, and only preserves the H-bond with Gly668 (Fig. 3H). In addition, Tyr671 interacts hydrophobically with Met560 from a long α-helix 549–568 (strong contact) and Lys233 from the loop 222–237 (periodic contact), compared with Cys671 loosing entirely the former and almost entirely the latter contacts (Fig. 3G,H; Supplementary material 4: Tables 6 and 7). Moreover, the replacement of tyrosine with cysteine within the 669–684 α-helix perturbs the symmetry of the N-terminal end of this α-helix (residues 669–674), resulting in the disruption of periodic hydrogen bonding and subsequently weakened, less persistent local α-helical structure (see secondary structure assignment for this α-helix in the wild type and Tyr671Cys mutant in Supplementary Material 5).

However a different picture can be seen in the case of the p.His841Asp mutation, which is located in the surface α-helix 834–849, nearby the site of the p.Arg758Cys mutation (Fig. 4A,B). In the mutant structure, Asp841 is coordinated with hydrogen bonds more persistently than His841 in the wild-type structure(Fig. 3K,L). However, the imidazole side chain of His841 is engaged in several hydrophobic contacts, including with Phe844 and Leu845 from the same α-helix and with Arg364 from a surrounding short surface loop 232–234 (Fig. 3K). Such a stabilization cannot be reached upon mutation to a polar residue of aspartate and hence the p.His841Asp mutant loses all the mentioned hydrophobic contacts, rendering histidine (His841 in the wild-type structure) as an overall better stabilized residue (Fig. 3K,L, Supplementary Material 4: Tables 10 and 11).

The other two mutants, p.Asp81Gly (Fig. 3A,B) and p.Ser555Ile (Fig. 3E,F), show less distinct rearrangements, accounted for as differences in local interaction networks with respect to the wild-type structure. Asp81, located at the dimerization interface in the loop 71–83 (Fig. 4B), is strongly saturated with H-bonds to Arg78; these interactions appear to be crucial in stabilizing the position of Asp81 in the wild-type structure. Nevertheless, other H-bond contacts of Asp81 are very limited and even less persistent than the hydrogen bonds maintained in the mutant structure by Gly81 (Fig. 3A,B, Supplementary Material 4: Table 1). Ser555, located in the core α-helix 549–568 (Fig. 4A), maintains a very strong H-bond with neighboring Glu544 and two much less persistent H-bonds with Glu551 and Lys559 (Fig. 3E), compared to the lack of the Glu544 contact and more persistent contacts with Glu551 and Lys559, maintained in the p.Ser555Ile mutant structure by Ile555 (Fig. 3F). In addition, the aliphatic side chain of Ile555 is mildly engaged in hydrophobic interaction with the pyrrolidine side chain of Pro546, whereas Ser555 lacks the hydrophobic stabilization at all (Fig. 3E,F, Supplementary Material 4: Tables 4 and 5).

Contrary to the other analyzed mutations, the local effect of the p.Lys132Met mutation, located close to the dimerization interface at the C-terminal end of the β-strand 126–132 (Fig. 4B), is neither unfavorable nor at most neutral as it seems to stabilize the local protein structure. This can be observed in the larger occupations of both hydrogen bonds and especially hydrophobic interactions maintained by Met132 in comparison to Lys132 (Fig. 3C,D). Met132 forms, both as a donor and acceptor, persistent H-bonds with Leu115 from a surrounding loop 106–115. Moreover, there is a strong hydrophobic contact between side chains of Met132 and Leu115 (Fig. 3C,D). Conversely, Leu132 interacts with Leu115 less strongly, forming with it a less persistent H-bond (only as a donor) and maintaining a much less persistent hydrophobic contact (Fig. 3C). Besides, unlike Leu132, Met132 maintains yet another stabilizing interaction, *i*.*e*. a very strong hydrophobic contact with Ala135 from a surrounding short loop 133–136 (Fig. 3C,D, Supplementary Material 4: Tables 2 and 3).

#### Global mutation effects

Stability of the dimerization interface in view of the effects on central α-helices: Parameters from the *PDBePISA* evaluation of dimerization interfaces in the average structures from the stable parts of trajectories of the wild-type ANO5 and its six single mutants are shown in Table 4. There is a diversity of the calculated interface surfaces and the estimated solvation free energy gains upon formation of the interface. The ΔG P-values, indicating the P-value of the observed solvation free energy gain, are either below or above 0.5. This allows the distinction between hydrophobic, interaction-specific interfaces, observed for wild type ANO5 and its mutants: p.Tyr671Cys, p.His841Asp and p.Lys132Met (ΔG P-values below 0.5), from interfaces that are more “random”, *i*.*e*. less hydrophobic than they optimally could be, observed for ANO5 mutants p.Asp81Gly, p.Ser555Ile and p.Arg758Cys (ΔG P-values significantly above 0.5). Therefore, the ΔG P-value is an important overall indication of the differences in hydrophobic character of the dimerization interfaces in the modeled sample.

The dimerization interface in the ANO5 structure and its mutants is composed of four central α-helices. Each subunit contributes two α-helices [609–639 (A1) and 692–715 (A2) in subunit A and, symmetrically, 1523–1552 (B1) and 1605–1628 (B2) in subunit B] together with surrounding loops and a few patches from other helices (see Fig. 4A,B). As expected, based on their buried location in the protein, these central α-helices are of mostly hydrophobic character. The conservation of this hydrophobic core during MD simulations can be evaluated by counting up the hydrophobic contacts present in the trajectories and in the average structures between hydrophobic residues from each of the central α-helices (Supplementary Material 4: Table 12, and Supplementary Material 5). These results are in accordance with the *PDBePISA* ΔG P-values. The highest number of contacts between subunits (20) was predicted for the wild type protein with 12 contacts between subunits A and B of the dimer (Supplementary Material 4: Table 12; Fig. 4C, left panel). The low and thus disadvantageous number of contacts is calculated for the p.Asp81Gly and p.Arg758Cys mutants with five and four contacts, respectively, and only with three contacts between residues in subunits A and B of both mutants (Supplementary Material 4: Table 12 and Fig. 4D,H, left panels). The higher number of contacts, 15, is predicted for the p.Ser555Ile mutant with nine intersubunit contacts (Supplementary Material 4: Table 12 and Fig. 4F, left panel). Similar values were calculated for other mutants with 17(6) for p.Lys132Met, 14(9) for p.His841Asp and 14(7) for p.Tyr671Cys (numbers in parenthesis reflect the intersubunit contacts) (Supplementary Material 4: Table 12; Fig. 4E,H,I, left panels). The percentage of trajectory occupancies of all hydrophobic contacts between residues of the four central α-helices of the analyzed structures are listed in Supplementary Material 4: Table 12. Moreover, only the wild-type protein shows the contacts from the A1 α-helix distributed equally to the B1 and B2 α-helices, whereas in all the mutant structures the contacts from the A1 α-helix reach exclusively the B1 α-helix, putting the B2 α-helix totally aside. It means the larger segment of interface stabilization, embracing the A1, B1 and B2 central α-helices, to be present only in the wild-type structure, in contrast to all mutant structures contributing to the stabilization pool only the A1-B1 α-helices’ interactions. Furthermore, in the trajectory of the wild-type structure there are two strong H-bonds linking the A1 α-helix with the B1 α-helices (Tyr632-Ile1540, occupied at 71.9% of the analyzed trajectory) and B2 (Ile627-Trp1623, occupied at 57.4%), which seem to consolidate even further the dimerization interface. On the contrary, in their own trajectories, the mutant structures lack similarly persistent H-bonds at the central helices. Besides, all H-bonds occupied above 10% of the mutants’ trajectories (between Asn626-Asn1550, occupied at 45.2% in the p.Asp81Gly mutant; between Trp638-Thr1532, occupied at 31.7% in the p. His841Asp mutant; and between Trp638-Thr1532, occupied 27.5% in p.Lys132Met) are formed solely between the A1 and B1 α-helices. Unlike in the wild-type protein, the B2 α-helix is not engaged in all those H-bonds (Fig. 4C–I and Supplementary Material 4: Table 13).

The secondary structure content throughout the simulations (see Supplementary Material 5) shows that the p.Asp81Gly and, to a lesser extent, p.His841Asp mutations perturb the central helices. This results in their accumulated average α-helical assignments of 70.4% and 74.1%, respectively, compared to 78.7% for the wild-type structure. The values for the p.Tyr671Cys, p.Ser555Ile and p.Lys132Met mutants were similar to that of the wild type and were 76.7%, 77.8% and 77.9%, respectively. Interestingly, the p.Arg758Cys mutation even strengthened the α-helical assignment of the four central helices, bringing it to an accumulated average of 85.2% (Supplementary Material 5).

Essential Dynamics: Essential dynamics was performed to enable identification of concerted motions that are often fundamental for protein functions^31–33^. In the present work, three Principal Component Analyses (PCA) were performed: first based on the decomposition of the global conformational space (PCA-1), second on the conformational space explored by four central helices (PCA-2), and the third on the distances between the central helices (PCA-3) (Figs 4C–I, right panels, and 5, as well as Supplementary Materials 6 and 7).

The eigenvalues against the corresponding eigenvector index for the first 10 modes of motion are plotted for the PCA-1 and PCA-2 analyses (Fig. 5A,B, respectively). It demonstrates that only few modes of motion are required to explain the majority of variance in the motions. Indeed, the first eigenvectors (PC1) alone explain 29.5–37.6% or 13.4–40.6% of the total variance in the motions observed in PCA-1 or PCA-2, respectively. Together with the second (PC2) as well as the second (PC2) and third eigenvector (PC3) the explanatory rate increases to 41.0–50.3% and 30.5–62.6%, for PCA-1 and PCA-2, respectively. The correlation coefficients (r^2^), computed from the time evolution of trajectories projected on the first eigenvector and plotted for each pair composed of the wild-type protein and mutant, show the motions associated with PC1 being either analogous (ANO5 versus p.Ser555Ile and ANO5 versus p.His841Asp) or strongly analogous between the wild-type protein and the mutant systems in PCA-1. On the other hand, r^2^ are very poorly correlated between ANO5 and its mutants in PCA-2 (Supplementary Materials 6 and 7). It demonstrates that while the motions are similar on the global scale, the local motions pertaining to the central helices are not similar at all, *i*.*e*. they do not repeat the same pattern.

Investigating further the eigenvalues against eigenvector index plots in Fig. 5B, a very shallow PCA-2 profile can be observed for ANO5, indicating the concerted motions associated with different eigenvectors in ANO5 to be of similar explanatory relevance. In addition, the PC1 motions in mutant structures have slightly (p.Ser555Ile) through moderately to largely (p.His841Asp and p.Lys132Met) higher amplitudes compared to the PC1 motion in ANO5. It indicates that the central helices in mutated structures undergo larger PC1 motions compared to the wild-type protein (see Fig. 4C–I, right panels, Supplementary Materials 6 and 7). It points to supposedly destabilizing effects of the mutations, as can be concluded also from the comparison of the plots depicting the PCA-2 motions associated with PC1 (Fig. 5 and Supplementary Material 7). The consolidated position and moderate motions of the central helices in the average structure from the PC1 motion mode of ANO5, compared with less ordered position and more “random” motions of those helices in all mutant structures are noticed there. Moreover, the “subtle” PCA-2 motions associated with PC2 and PC3 in ANO5 (see Supplementary Materials 6 and 7), worth of considering due to similar explanatory relevance of the first three modes of motion of ANO5, do not overrule the picture emerged from the comparison of the motions associated with PC1.

An incisive dependence pattern of the amplitudes (eigenvalues) associated with eigenvectors, observed in PCA-2, does not occur in PCA-1, where the amplitudes of the PC1 motions are less mutually different and the eigenvalue associated with PC1 in ANO5 is not the smallest one in the series (Fig. 5A,B, and Supplementary Materials 6 and 7). Nevertheless, the concerted motions associated with PC1 in ANO5 appear to be the most ordered in the series, with few surface segments and the central loop 1409–1424 from subunit B moving while the rest of the structure remaining virtually still (Supplementary Material 7). Overall, all mutants appear to be more “restless”, with concerted motions involving large parts of their structures, which is especially evident in the p.Tyr671Cys, p.Lys132Met, p.R758C and p.His841Asp mutants (see Supplementary Material 7). But given that, the results of PCA-1 do not seem to provide a clear message on the differences potentially destabilizing the mutant structures compared to the wild-type protein.

The PCA-3 analysis can be considered as complementary to PCA-2. The two obtained features were able to explain ~90% of variance in the dataset. Results plotted on Fig. 5C show the time development of projections of the functions characterizing distances between the central α-helices along PC1 vs PC2. Figure 5C presents a separate localization and concentrated appearance of the wild-type protein (black) and different localizations and more dispersed appearances of all mutant structures, though less so of the p.Ser555Ile mutant (blue). It suggests (and possibly confirms) the stable behavior of the central α-helices in the wild-type structure in contrast to less stable/more perturbed behaviors of those helices and, in turn, of the dimerization interface, in mutant structures. In particular, the p.Asp81Gly and p.His841Lys mutants (red and orange, respectively) appear to be significantly different from all other mutants and from the wild-type protein, while mutants p.Lys132Met (green), p.Arg758Cys (magenta), p.Tyr671Cys (cyan) and p.Ser555Ile (blue) also differ from the wild-type protein but exhibit mutual similarity when described with two PCA-derived features. p.Asp81Gly and p.His841Asp mutants are the ones in the series with weakened α-helical secondary structure assignments within the central α-helices. Besides, A1 and A2 α-helices in the p.His841Asp mutant are found wandering quite a lot during the first mode of motion (see Supplementary Material 7), the one explaining as much as 46% of the total motion variance in PCA-2. This wandering perturbs their between-helices-distances, which appears to be reflected in a very strongly dispersed appearance of His841Asp on the plot, with A1 and A2 helices either in contraction or separated (approximately two parts of the His841Asp appearance on the plot in Fig. 5C).

## Discussion

We presented herein clinical and diagnostic data of five Polish patients with *ANO5* heterozygous mutations and clinical diagnosis of LGMD2L. Analyses of both clinical phenotypes and muscle morphology (based on histochemistry and MRI) revealed a high degree of variability. The MRI imaging also showed a high degree of variability of changes in different muscles in the same patient, making the LGMD2L picture even more complicated.

Genetic analysis identified nine mutations, among them three: p.Tyr23Ter, p.Lys132Met and p.Trp401Ter were not yet characterized and the latter two not even registered in the gnomAD database. The other six mutations: p.Asn64LysfsTer15, p.Asp81Gly, p.Ser555I, p.Tyr671C, p.Arg758C and p.His841Asp have been already described in the European population^9,21,22^ as damaging, implying their contribution to the observed pathology.

The two newly characterized nonsense mutations introduce a premature stop codon and most probably lead to degradation of a truncated protein through proteasomal pathways^23,31,32^. The lack of the full length ANO5 protein was recently shown for the homozygous p.Asn64LysfsTer15 (c.191dupA) mutation that in fact introduce a premature stop codon (see^34^). In many genes (e.g. *DMD*, *RYR1*) the presence of frameshift or nonsense mutations is often associated with a severe disease course^35^.

To characterize the effects of missense mutations on the ANO5 structure, and possibly function, we employed molecular dynamics analyses. For this purpose, models of the monomer and dimer structure were created. The models were generally analogous to the models presented by Andreeva *et al*.^17^. However, the number of transmembrane helices depicted in our model surpasses the number declared by those authors. Unfortunately, more detailed comparison of both models is impossible due to the lack of access to the aforementioned models.

Analysis of the local effects indicated that the most visible changes were observed for p.Tyr671C and p.Arg758C mutants, both associated with the introduction of a cysteine residue, much smaller than the original arginine and tyrosine residues but also prone to form new interactions. On the other hand, p.Asp81Gly (introducing a small glycine residue) and p.Ser555Ile (introducing a hydrophobic isoleucine residue) mutations have much less impact on local conformational rearrangements. Contrary to that, the novel p.Lys132Met (introducing a similar in molecular mass but non-polar and sulphur containing methionine residue) mutation seems to rather stabilize (stiffen) the local protein structure. Interestingly, Asp81 and Tyr671 as well as Lys132 residues are situated either at the dimerization interface (Asp81) or in its vicinity (Tyr671 and Lys132). Thus the local effects of the mutations on the protein structure are variable and do not depend on the localization of the mutated residues within the molecule. However, they could influence the protein dimerization and so we examined the effects of the mutations on the dimerization interface.

We predict that all the analyzed mutations affect dimer formation. In particular, the Asp81Gly and Arg758Cys mutations appear to have a strong detrimental effect on the stability of the dimerization interface as shown by the high ΔG P-values, low overall numbers and lack of balanced pattern of hydrophobic contacts, lower SSC (for p.Asp81Gly) as well as lack of (for p.Arg758Cys) or weakened (for p.Asp81Gly) hydrogen bonds. More elaborate although ultimately disadvantageous seem to be the effects of the p.Ser555Ile (the high ΔG P-value, lack of balanced hydrophobic interactions pattern and lack of strong hydrogen bonds) and p.His841Asp (lower SSC, lack of balanced hydrophobic interactions pattern, weaker hydrogen bonds compared with the wild-type system) mutations. The other two mutations, p.Lys132Met and p.Tyr671Cys, exhibit both a less balanced hydrophobic interactions pattern, and the lack of (for p.Tyr671Cys) or weakened (for p.Lys132Met) hydrogen bonds, with both these features expectedly lowering the stability of the dimerization interface.

Moreover, essential dynamics analysis revealed that all analyzed mutations affect the concerted motions of the ANO5 dimer and have supposedly destabilizing effects as central α-helices of mutant structures undergo larger motions with respect to the wild type protein. All the mutants appear to be more motile (restless) with larger parts of the protein engaged in the concerted motions. In particular, this was most evident in p.Lys132Met, p.Tyr671C, p.Arg758C and in particular in p.His841Asp. Thus the results of essential dynamics highlight the differences between the wild-type protein and the mutant structures on the ground of less stable central α-helices at the dimerization interface, that in mutant proteins undergo larger and seemingly more random motions. These dynamic structural differences could therefore result in damaging/deleterious effects on the protein function.

The question arises whether and how particular mutations affect the protein conformation and influence the patients’ phenotype. Patient 1, with most severe dystrophic changes, bore a p.Trp401Ter truncation mutation and p.Asp81Gly missense mutation. The nonsense mutation introduces premature stop codon located in the proximity of the final exon-junction thus suggesting elimination of the mRNA through the nonsense mediated decay. The latter mutation was also found in patient 2 where it co-existed with a missense p.Arg758Cys mutation, which also had negative impact on the dimerization interface. Despite carrying mutations that negatively affect dimerization, patient 2 presented mild myopathic changes (in clinical and MRI examinations) but with early disease onset. Interestingly, the same pair of mutations has been already identified in a Finnish male patient with the similar disease onset and clinical phenotype^20^. Of note, the p.Arg758Cys mutation is considered as a founder mutation in the Finnish population^36^. Patient 3, bearing the novel p.Tyr23Ter truncation and known missense p.Ser555Ile mutation (with disadvantageous effect on dimer formation), also presented mild muscle involvement but with disease onset in third decade of life. Patient 4 bears a novel p.Lys132Met mutation accompanied by a known p.His841Asp mutation that was first described in a British patient as homozygous and was predicted to be pathogenic^22^. Our analyses showed that both mutations affect the dimerization interface with p.His841Asp being more disadvantageous. This patient, with onset in his fourth decade presented a moderate phenotype. Patient 5 carries the most common p.Asn64LysfsTer15 mutation predicted to have deleterious effect on the protein function, and p.Tyr671Cys missense mutation, which as we showed strongly affected ANO5 conformation. This patient had disease onset in her third decade and presented advanced dystrophic changes.

The complexity to the picture is aided by the fact that numerous WES analyses have revealed mutation burden in genes other than *ANO5* that are also associated with LGMD phenotypes (for example^9,20,23^). These were depicted by prediction softwares either as benign/moderate or as deleterious/damaging and hence possibly pathogenic. In our study, such accompanying variants were also found and differed among patients. Here, we will focus on those depicted by the prediction programs as potentially most pathogenic. Patient 1 had potentially phenotype-modifying missense variants found in *BAG3*, coding for BAG3 (BCL2 associated athanogene 3), a protein involved in the chaperone-assisted selective autophagy (CASA) pathway^37^ and in *FLNC*, coding for filamin C (FLNC), a protein involved in actin cytoskeleton organization. Interestingly, a missense *FLNC* variant was also identified in patient 4. Both *FLNC* variants, predicted as damaging, are located within Ig-like domains, mutations in which are also often associated with cardiomyopathy phenotypes^38^. It is noteworthy that patient 1 had cardiac failure but so far patient 4 has not reported any heart problems. The reported herein p.Ser185Ile *BAG3* variant could affect chaperone function of the protein as even a mild impairment of the function could cause abnormalities in the sarcomere structure or dysfunctional protein folding quality control mechanisms^39,40^. Therefore it cannot be excluded that for patient 1 the observed muscle phenotype of *ANO5* could be also exacerbated by *BAG3* and *FLNC* variants. Patient 2 carries a variant in *NEB*, encoding a large protein regulating the length of thin filament in the contractile apparatus. *NEB* mutations are associated with a wide pattern of myopathic symptoms, and are often linked with nemaline myopathies^41,42^. We did not find nemaline rods in patient’s 2 biopsy indicating that the effect of this *NEB* mutation on the clinical phenotype seems to be rather minor. Patient 3 carries a known variant in *RYR1*, encoding skeletal muscle-type ryanodine receptor 1 (RYR1). Over 500 mutations have been mapped to *RYR*s and the majority of them are implicated in human diseases such as malignant hyperthermia as well as RYR1 myopathies, previously classified as central core disease (CCD) and multiminicore disease^43,44^. Identified *RYR1* mutation, affecting a repeat within the cytoplasmic part of the protein, is reported in the ClinVar database with a note that there are conflicting interpretations of pathogenicity. Therefore we cannot conclude whether or not this variant could contribute to the observed phenotype of patient 3. Patient 4, besides the *FLNC* mutation mentioned above, carries mutations in *COL12A1* and *LAMA2*, encoding isoforms of collagen 12 and extracellular matrix glycoprotein laminin A, respectively. Mutations within both genes could have deleterious effects on the skeletal muscle organization and function as they are associated with several myopathies, including LGMD, collagenoses and merosin deficient congenital muscular dystrophy^45,46^. The *COL12A1* variant is listed in the ClinVar database as likely benign while the one in *LAMA2* has contradictory predictions from benign to deleterious. We saw an increase in endomysial connective tissue infiltrations that could result from changes in collagen structure, however, they are much smaller than the ones observed in patient 1, who did not have variants in the gene encoding this collagen isoform. Moreover, it is also possible that minimal endomysial infiltrations could be associated with effects of mutation with *LAMA2*. However, mutations in both genes do not seem to greatly contribute to the observed phenotype. Interestingly, variants in several other genes involved in cell adhesion, namely *AGRN*, encoding agrin, a protein crucial for the development of the neuromuscular junction (NMJ), *ITGA7*, encoding integrin subunit α7, and *LAMB2*, encoding laminin B2 isoform, were identified. Both *AGRN* and *LAMB2* are associated with congenital myasthenic syndromes, associated with basal-lamina dysfunction^47^ and *ITGA7* mutations are associated with congenital muscular dystrophy^48^. Identified variants in these three genes could possibly affect sarcolemma and synaptic activity at the NMJ. Thus they could to some extent explain the observed phenotype, especially the changes in muscle morphology (as examined by MRI and histochemistry).

There is also another possibility that is beyond a scope of this study that changes evoked by *ANO5* mutations (and/or by mutations in other muscle-related genes) could have effect on functions of unmodified genes. It could be speculated that impairment of ANO5 might affect other anoctamin isoforms and/or proteins involved in the same processes, in which ANO5 is engaged.

In conclusion, we have described five Polish LGMD2L patients with recessive mutations in *ANO5* that according to the criteria proposed by Antonarakis *et al*.^49^ and MacArthur *et al*.^50^ are highly likely to be disease-causing according to ACMG criteria (see Table 3). Our data, in frame with other recent results, indicate that recessive *ANO5* mutations are a frequent cause of muscular dystrophy and are population-specific^51–54^. We showed that *ANO5* mutations (possibly *in concerto* with other variants in numerous genes linked with myopathies) are associated with the observed phenotypes. The severity of the clinical and morphological muscle impairment seems to be associated with the location of the mutation, and therefore its effect on ANO5 structure and function. However, there is not a direct correlation between the effects of mutations and clinical picture, probably due to mutation burden in other muscle-related genes. Finally, our data seem to support the ongoing hypothesis that LGMD and other dystrophies/myopathies could be in fact oligogenic disorders, with few mutated genes contributing unevenly to the development of the disease^23,55^.

## Materials and Methods

### Magnetic Resonance Imaging (MRI)

The whole body MRI (WB MRI) was performed on 1.5 T scanner (Siemens Avanto) with a multi-sequence imaging protocol. T1-weighted, T2-weighted and STIR (Short Tau Inversion Recovery) sequences were obtained in coronal planes with 5 mm slice thickness while T1-weighted and STIR sequences were obtained in axial planes with 8 mm slice thickness. Muscle bulk was assessed on T1-weighted images. Muscles were scored on axial T1-weighted sequences with the semi-quantitative^56^ visual scale modified by Fischer *et al*.^57^ to estimate the degree of muscle degeneration. STIR sequences were used to assess the presence and symmetry of edema-like changes.

### Muscle biopsy

The open muscle biopsies of the probands biceps brachii or vastus lateralis were performed, and the muscle specimens were routinely processed for further analyses.

### Light microscopy

Biopsied muscles were frozen in isopentane cooled in liquid nitrogen, cut on a cryomicrotome with a slice thickness of 8 µm and stained with the routine battery of histological and histochemical methods.

### Genetic analyses

DNA was extracted from peripheral blood of the proband using standard methods^58^. Whole exome sequencing (WES) was performed in two institutions: samples of patients 1 and 2 were sequenced commercially at Oxford Gene Technology (Oxfordshire, United Kingdom), and samples of patients 3, 4 and 5 were sequenced at the Broad Institute’s Genomics Platform as part of the MYO-SEQ project coordinated by the John Walton Muscular Dystrophy Research Centre, Newcastle University.

Samples 1 and 2 were sequenced using SureSelect Human All Exon v4 enrichment kit and 100 bp paired-end sequencing on the Illumina HiSeq2000 platform. Fastq read files were generated from the sequencing platform via the Illumina software. The paired-end reads were aligned to the human genome build 37 (hg19) reference using Burrows-Wheeler Alignment (BWA) package^59^. Duplicate reads were removed with Picard and base quality Phred scores were recalibrated using Genome Analysis Toolkit (GATK) covariance recalibration^60^. Alignments were viewed with Integrative Genomics Viewer (IGV)^61^. Sequencing resulted in >80% of targets covered at least 20x and a mean target coverage was 55x. Single Nucleotide Polymorphism (SNPs) and insertions/deletions (indels) were called using the GATK Unified Genotyper Annovar for initial variant annotation^62^ and further annotation, filtering and analysis performed on the Galaxy platform (on PL-Grid Infrastructure).

WES for samples 3, 4 and 5 was performed at the Broad Institute’s Genomics Platform, using Illumina exome capture and 76 bp paired-end reads on the Illumina platform. Fastq read files were generated from the sequencing platform via the Illumina software. The paired-end reads were aligned to the human genome build 37 (hg19) reference using Burrows-Wheeler Alignment (BWA) package^59^. Duplicate reads were removed with Picard and base quality Phred scores were recalibrated using Genome Analysis Toolkit (GATK) covariance recalibration (McKenna *et al*.^60^). Alignments were viewed with IGV. Sequencing resulted in >80% of targets covered at least 20x and a mean target coverage was above 80x. SNPs and indels were called using the GATK HaplotypeCaller Default filters were applied to SNP and indel calls using the GATK Variant Quality Score Recalibration (VQSR) approach. Lastly, the variants were annotated using Variant Effect Predictor (VEP).

Filtering was based on the allele frequency in ExAC database (<3% for variants in genes already associated with LGMD, and <1% for variants in other genes), and predicted pathogenicity (predicted pathogenic by at least one out of Mutation Taster, PolyPhen2, and SIFT software). Prioritization was based on inheritance mode, on the predicted effect with truncating and elongating variants being evaluated more carefully, on predicted pathogenicity, and on known association with myopathic phenotypes reported in the Human Phenotype Ontology. Standardized phenotypic information was uploaded to PhenoTips^63^. Analyses were made with Exomiser2, PhenIX and Exome Walker with prioritization of variants based on possible association with Human Phenotype Ontology term “limb-girdle muscular dystrophy” and based on random-walk analysis of protein interaction networks^64^. *ANO5* mutations and selected mutations in other genes (*BAG3*, *FLNC*, *RYR1*) were confirmed using direct, fluorescent sequencing (ABI 3130 Gemetic Analyzer, Applied Biosystems, USA).

### Models of ANO5 and its mutants

A model of dimeric wild-type ANO5 protein was prepared in two steps: (i) the monomeric structure of ANO5 was generated using the intensive mode of Phyre2 algorithm^65^ based on the human ANO5 wild-type sequence (Q75V66 in the UnitProt Knowledgebase), and (ii) the monomer into dimer conversion was carried out using the M-ZDOCK server^66^. Modeling of both steps was performed with default settings implemented in the Phyre2 intensive mode and M-ZDOCK algorithms. 602 out of 913 residues in the ANO5 sequence (66%) were modeled by homology and rated by the Phyre2 algorithm as highly confident (above 90% of accuracy), while the rest of the structure was modeled *ab initio* due to the lack of respective homology motifs.

The following single mutations were incorporated in the ANO5 dimer wild-type model in order to investigate their effect on the 3D structural level: Asp81Gly, Lys132Met, Ser555Ile, Tyr671Cys, Arg758Cys and His841Asp (Fig. 4A,B). Both the wild-type structure and all mutant structures were prepared to MD simulations according to the following protocol. Hydrogen atoms were added in *tleap* from AmberTools16^67^ according to standard protonation states of the amino acids. Resulting structures were immersed in truncated octahedral boxes of water molecules (TIP3P model^68^) extended up to 9 Å away from the protein surfaces. Monovalent sodium ions were placed in calculated positions to keep the neutrality of the simulation boxes.

### Molecular dynamics (MD)

The simulation setup was as follows. Initially, the models were subjected to mixed steepest descent and conjugate gradient energy minimizations carried out in two steps involving (i) only solvent molecules and (ii) the whole systems. Then, gradual heating to 300 K was performed in three 10 ps intervals (0 → 100 K, 100 K → 200 K and 200 K → 300 K), followed by equilibrations at 300 K lasting for 10 ns. Finally, productive MD simulations in the NpT ensemble (conserved number of atoms (N), pressure (p) at 1 atm and temperature (T) at 300 K) were performed and lasted for 130, 180 or 230 ns, depending on the simulation. Pressure and temperature were monitored by the isotropic position scaling and the weak-coupling algorithms, respectively, with both coupling times of 1 ps. Periodic boundary conditions were applied throughout simulations. Long-range electrostatic interactions were handled using the particle mesh Ewald method^69^ with a charge grid spacing of approx. 0.9 Å. All bonds involving hydrogen atoms were constrained using the SHAKE algorithm^70^ with a relative tolerance of 0.00001 Å, allowing a time step of 2 fs. Trajectories were saved at 2 ps intervals. All minimizations and MD simulations except the productive part were carried out with *sander* from AmberTools16 using the ff14sb force field^71^. Productive MD simulations were performed with *pmemd* using the same force field.

### Trajectory analysis

Root mean square deviations (RMSD) of the backbone atoms from the collected trajectories have been calculated with *cpptraj*^72^ from AmberTools16 to assess the conformational stability of the wild-type and mutant models. Those stable parts of each trajectory which spanned at least 40 ns were used for further analysis with *cpptraj*, including computing average structures, hydrogen bond (HB) and hydrophobic interaction (HI) occupancies and secondary structure content. The average structures have been evaluated with the *Protein interfaces*, *surfaces and assemblies service PISA at the European Bioinformatics Institute (*http://www.ebi.ac.uk/pdbe/prot_int/pistart.html*)*^73,74^. The HB occupancies were calculated based on the following limits: 3.2 Å for the donor-acceptor distance and 120° for the donor-hydrogen-acceptor bond angle. The HI occupancies were calculated for the hydrophobic contacts between two residues in the range of 3.5–5.5 Å.

### Essential dynamics (ED)

In general, the method is based on the construction of the covariance matrix of coordinate fluctuations, which is diagonalized in order to obtain the eigenvectors and eigenvalues that represent the concerted motion in the system. While the eigenvectors indicate the directions of motion, the corresponding eigenvalues reflect the amplitudes of motion along those directions. The trajectories have been sampled uniformly either to obtain 2,500 frames for each individual analysis (PCA-1 and PCA-2) or every 50^th^ frame to obtain 4,406 frames for combined analysis (PCA-3). In the latter analysis, all possible distances between four central α-helices were measured. The objective was to reduce the number of features describing those distances to 2. The helices were defined as follows: 609–639 (A1) and 692–715 (A2) from subunit A and 1523–1552 (B1) and 1605–1628 (B2) from subunit B. All analyses were carried out with either pyPCAZIP, a python-based reimplementation of the PCAZIP package (PCA-1 and PCA-2)^75^ or scikit-learn Python package (PCA-3)^76^. The plots of concerted motions were prepared with the help of *cpptraj* and the Normal Mode Wizard VMD plugin^77,78^ and rendered with POV-Ray 3.6 (PCA-1 and PCA-2)^79^.

### Ethics Statement

Written informed consent was obtained from all patients for muscle biopsy analyses as well as for genetic testing, according to the Declaration of Helsinki. The study was approved by the Ethics Committee of the Warsaw Medical University (Warsaw, Poland) in compliance with national legislation and with the Code of Ethical Principles for Medical Research Involving Human Subjects of the World Medical Association.

## Supplementary information

Supplementary Materials 1-7

## Data Availability

The datasets generated during and/or analyzed during the current study are available from the corresponding author on a reasonable request.
