# Supplementary material for: ANO5 mutations in the Polish limb girdle muscular dystrophy patients: Effects on the protein structure
Source: Sci Rep. 2019 Aug 8;9:11533. doi: 10.1038/s41598-019-47849-3 (PMC6687736; doi:10.1038/s41598-019-47849-3)

ANO5 mutations in the Polish limb girdle muscular dystrophy patients: Effects on the protein structure

Jarmula A., Łusakowska A., Fichna J.P., Topolewska M., Macias A., Johnson K., Töpf A., Straub V., Rosiak E., Szczepaniak K., Dunin-Horkawicz S., Maruszak A., Kaminska A.M., Redowicz M.J.

Supplementary Materials Legend

Supplementary Material 1. Description of the patients

Supplementary Material 2. Sequencing data of patients 3-5

Supplementary Material 3. MRI analysis

Supplementary Material 4. Tables

Supplementary Material 5. Secondary Structure Content.

Supplementary Material 6. Principal Component Analyses (Essential Dynamics)

Supplementary Material 7. Motions

Jarmula, Lusakowska et al.

SUPPLEMENTARY MATERIAL 1 - Case Descriptions

Part I. Pedigrees of the examined families

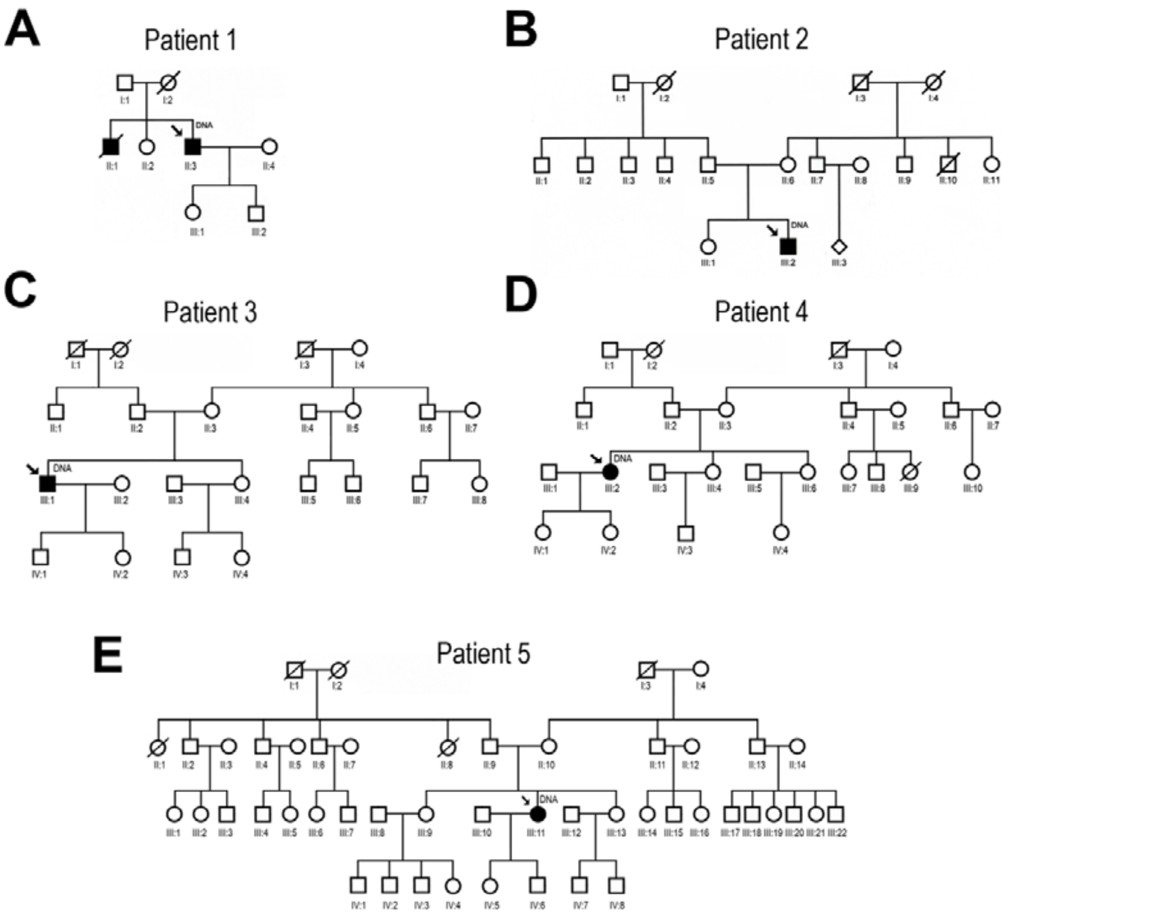

Fig. 1. Pedigree diagrams of LGMD patients. A-E, diagrams of probands' families. Arrows point to probands.

II. Case Descriptions

Patient 1*:* a 54-year old Caucasian male was admitted with symptoms of LGMD. The proband (Part I A, II:3) was a third child of unaffected, non-consanguineous parents. His deceased elder brother had an undiagnosed neuromuscular disease with gait impairment, while his sister, and his both children (30 and 28 year-old) are clinically unaffected. The proband reported gait problems with a gradual onset in the third decade, moderate myalgia and arthralgia. At 49 years, he underwent cervical spine surgery without improvement, afterwards he noticed slowly progressive weakness and clumsiness of the hands. The proband's medical history included posttraumatic epilepsy treated with Depakine and Lamotrigine, ethanol addiction and abuse, and severe

depression, as well as arterial hypertension, glaucoma and hepatitis B. He was hospitalized twice due to cardiac failure with massive generalized oedema and increased creatinine level of unknown etiology. At 54 years, the proband presented with limb-girdle muscular weakness, mild and asymmetrical in the upper limbs, severe in the lower limbs, with posterior thigh muscles strength most impaired. Biopsy of biceps brachii was then performed. Deep tendon reflexes were absent in lower limbs, there was visible thigh muscle atrophy. There was slight sensory deficit along L4 dermatome in the left lower limb. The proband was able to walk with two crouches with marked knee hyperextension. He required help with sitting up from prone position. Nerve conduction studies revealed axonal-demyelinating mild multifocal involvement of motor and sensory nerves indicating acquired character of peripheral neuropathy. Electromyography showed myopathic changes in the biceps brachii and rectus femoris muscles, while in the distal muscles there were signs of both myogenic and neurogenic process. The level of creatine kinase (CK) was elevated 19 times above normal upper limit. Biceps brachii muscle biopsy revealed advanced dystrophic features. Brain and spinal MR imaging did not show any significant abnormalities. Echocardiography was normal. We have no follow-up of the patient.

Patient 2: a male patient was referred to our neuromuscular department at the age of 22 because of myalgia and increased CK level. The patient is the second child of unaffected, non-consanguineous parents (Part IB; III:2). There is no neuromuscular disease history in the family, although slightly increased CK level was found in both parents (less than 1,5 x above the normal upper limit). The patient has a 31-old year unaffected sister, whose CK level is within normal range. At the age of 2 1, hyper-CKmia of 11000 U/L was detected after increased aminotransferase level in had been found in routine blood tests. The patient who was an athlete and a dancer was then asymptomatic. According to available documentation, hypertrophy of quadriceps femoris was noticed, electromyography was normal. In subsequent years the patient developed muscle pain localized mainly in the thighs and shoulders, evoked by exercise and exacerbated a few hours after the exercise. He also reported feeling of increased muscle temperature, trembling or pulsation within the muscles, both muscle and general fatigue, increasing exercise intolerance. He reported infrequent muscle cramps. Exercise tolerance was better in summer months than in winter. Apart from feeling of “heavy legs” during climbing stairs, he did not notice any muscle weakness. On neurological examination generalized muscular hypertrophy was seen, mild scoliosis with right-sided scapular winging, and mild pectus excavatum. Muscular strength was normal except for slight impairment of hip flexors bilaterally. Deep tendon reflexes in the upper limbs and knee reflexes were decreased, ankle jerks were preserved. No contractures were seen. The rest of the neurological examination was normal. CK level fluctuated between 2300-4000 U/L (about 20x

UNL). Electromyography revealed unspecific changes in lower limb muscles suggesting myopathy (motor unit potentials with slightly increased amplitude and shortened duration in vastus lateralis, borderline low motor unit potential amplitude in tibialis anterior muscle; biceps brachii electromyogram was normal). No spontaneous activity during rest was recorded. Motor and sensory nerve conduction velocities were normal. Echocardiography revealed mitral valve prolapse without hemodynamic impairment. 24h electrocardiogram did not show heart rhythm disturbances. Pulmonary function test were normal. In course of genetic diagnostics, type2 myotonic dystrophy was excluded and dystrophin gene analysis with MLPA did not reveal deletion or duplication. In a follow-up examination 8 years later, there were no new findings except for mild asymmetry of calf muscles. The patient remains fully ambulatory, able to stand up from the crouch, walk on toes or heels. There is no heart function or respiratory muscle impairment.

***Patient 3*: ** a 36-year old male patient was referred to the neuromuscular department because of an increased CK serum level (Part I C, III:1). He complained of mild episodic myalgia and muscle cramps. Eleven years before he was diagnosed for a liver disorder because of an increased level of transaminase and bilirubin. Viral hepatitis B and C were excluded. The liver biopsy showed nonspecific changes. The Gilbert syndrome diagnosis was established. At that time CK level was not assessed. One year before hospitalization in our department, the patient was diagnosed due to a high blood pressure and in routine laboratory tests the CK serum level was 15 times above the normal upper limit. The patient is the first child of unaffected, non-consanguineous parents. There is no neuromuscular disease history in the family. The patient was very active in sports since childhood. He is working as a policeman. On neurological examination, he presents with asymmetrical calf and quadriceps muscles hypertrophy, more prominent in the left lower limb. Muscular strength was normal (5/5 in all groups of muscles in MRC scale). Deep tendon reflexes were normal. No contractures were seen. CK level was 10 times the UNL (3108U/l, N.39-308). Transaminase and GGTP were elevated. Lactic acid curve was normal. Electromyography was normal in biceps brachii and vastus lateralis. However, neurogenic changes were observed in tibialis anterior musclesuch as an increased amplitude of motor unit potentials, duration in the upper limit and increased size index (SI). Electroneurography in sensory and motor nerves was normal. Muscle biopsy taken from the left vastus lateralis showed noncharacteristic moderate myopathic changes. Pulmonary function tests were normal. In course of genetic diagnostics, myotonic dystrophy type 2 as well as Pompe disease were excluded. Brain MRI imaging did not show any significant abnormalities. Spine MRI imaging showed L5-S1 discopathy.

Patient 4: a 38-year old female was referred to the neuromuscular department because of myalgia and weakness in lower limbs (Part ID, III:2). The symptoms started three months earlier without any triggering factors. At that time, she complained of severe myalgia and lower legs muscle weakness. She could not walk for some days and she used a wheelchair. Initially, she was diagnosed in a local hospital at a neurological department. The CK level was elevated about 8 times above the normal upper limit. EMG revealed myopathic changes. The diagnosis of polymyositis was suspected and steroid therapy (Encorton) in low dose (20mg) was started. Because of poor tolerance and worsening of the symptoms after steroids, the treatment was gradually stopped. Patient was referred to department of reumathology where the diagnosis of polymyosistis was excluded. At the age of 34 she underwent a partial strumectomy because of nodular goiter. The patient is the first child among three siblings of unaffected, non-consanguineous parents. There is no neuromuscular disease history in the family. On neurological examination, the patient presented with mild proximal muscle weakness in upper limbs and moderate proximal muscle weakness in lower limbs. All deep tendon reflexes were present and normal. The CK serum level was elevated to 3 times the upper limit. EMG as well as ENG tests were normal. The muscle biopsy showed moderate non-characteristic myopathic changes. No inflammatory cells infiltration were found. Spirometry was normal.

Patient 5**:** a 42-year old female, was referred to our neuromuscular department because of limb-girdle muscle weakness and difficulty in walking (Part IE, III:11). First symptoms appeared at the age of 25 years as weakness of right lower limb with accompanying lumbar pain. After treatment with non-steroid anti-inflammatory drugs, pain subsided but muscle weakness persisted. After a few years, she started to complain of weakness of the left leg resulting in difficulty in running and climbing stairs. At that time she also started to report mild proximal muscles weakness of upper limbs. Two years before admission to our department the CK level was elevated up to more than 20 times of upper normal limits, but EMG was normal. The patient was one of the three children of unaffected, non-consanguineous parents. There is no neuromuscular disease history in the family. On neurological examination the patient presented with mild proximal more than distal muscle weakness of lower limbs (more prominent on the left side), especially in posterior compartment. The patient could not walk on the toes whereas she was walking on the heels without problem. Deep tendon reflexes were normal in upper limbs and weak , but present in lower limbs. The CK serum level was about 9 times of upper normal limits elevated. The EMG revealed myopathic changes in biceps brachii whereas the EMG of vastus lateralis was within normal limit. Electroneurography (ENG) of motor and sensory nerves was normal. Spirometry was normal.

Jarmula, Lusakowska et al.

Supplementary Material 2

Detailed MRI analysis of muscles of patients 2-5. The following parameters were analyzed: muscle volume, muscle fat, fat symmetry and the presence of edema

Patient/Muscle/Parameter	Patient 2				Patient 3				Patient 4				Patient 5			
	Volume	Fatty	Fat symmetry	Edema	Volume	Fatty	Fat symmetry	Edema	Volume	Fatty	Fat symmetry	Edema	Volume	Fatty	Fat symmetry	Edema
**upper girdle**	0	0	1	0	0	0	1	0	0	0	1	0	0	0	1	0
**biceps brachii**	0	0	1	0	0	0	1	0	0	0	1	0	1	3	1	1
**brachialis**	0	0	1	0	0	0	1	0	0	0	1	0	0	0	1	0
**triceps brachii**	0	0	1	0	0	0	1	0	0	0	1	0	0	0	1	0
**forearm extensors**	0	0	1	0	0	0	1	0	0	0	1	0	0	0	1	0
**forearm flexors**	0	0	1	0	0	0	1	0	0	0	1	0	0	0	1	0
**thoracic wall**	0	0	1	0	0	0	1	0	0	0	1	0	0	0	1	0
**abdominal wall**	0	0	1	0	0	0	1	0	0	0	1	0	0	0	1	0
**paraspinal cervical, thoracic**	0	0	1	0	0	0	1	0	0	0	1	0	0	3	1	0
**paraspinal L/S**	0	0	1	0	0	2	1	1	0	1	1	0	0	4	1	2
**gluteus maximus**	0	0	1	0	1	2	1	0	1	1	1	0	0	2	1	0
**gluteus medius**	0	0	1	0	0	2	0	2	0	0	1	0	0	3	1	2
**gluteus minimus**	0	1	1	0	1	3	1	2	0	1	1	0	0	4	1	0
**sartorius**	0	0	1	0	0	0	1	0	0	1	1	0	0	1	1	0
**tensor fasciae latae**	0	0	1	0	1	3	0	0	0	1	1	0	0	4	1	0
**adductor longus**	1	3	0	1	0	4	0	2	0	0	1	0	0	0	1	0
**adductor magnus**	0	0	1	0	1	2	0	2	0	1	1	0	1	3	1	0
**gracilis**	0	0	1	0	0	0	1	0	0	0	1	0	0	0	1	0
**rectus femoris**	0	0	1	0	0	1	1	0	0	0	1	0	0	0	1	0
**vastus lateralis**	1	1	0	1	0	2	0	1	0	0	1	0	1	1	1	0
**vastus medialis**	0	0	1	1	0	3	0	1	0	0	1	0	1	3	0	1
**vastus intermedius**	0	0	1	0	1	2	0	2	0	0	1	0	1	1	1	0
**biceps femoris long head**	1	1	0	1	1	1	1	1	1	1	1	0	1	4	1	0
**biceps femoris short head**	0	0	1	0	1	2	1	1	0	0	0	0	1	2	1	2
**semimembranosus**	1	2	0	1	1	2	0	1	1	1	1	0	1	4	1	0
**semitendinosus**	1	1	1	0	1	1	1	0	0	0	1	0	1	3	1	1
**tibialis anterior**	0	0	1	0	0	2	0	1	0	0	1	0	0	0	1	0
**peroneus**	0	0	1	0	0	0	1	0	0	0	1	0	0	0	1	0
**medial gastrocnemius**	1	3	1	0	1	4	1	0	0	2	1	2	2	4	1	0
**lateral gastrocnemius**	0	0	1	0	1	3	0	2	0	0	1	0	1	4	0	1
**soleus**	1	1	0	1	1	1	1	1	0	0	1	0	2	4	1	1
**tibialis posterior**	0	0	1	0	0	0	1	0	0	0	1	0	0	0	1	0
**flexor hallucis longus**	1	1	0	2	0	0	1	0	0	0	1	0	2	3	0	2

Legend to the parameters:

Volume :

0=no change

1=muscle atrophy

2=hypertrophy

Fat:

Fatty degeneration according to modified Fischer grading scale:

0=no fatty degeneration

1=Mild

2=Moderate

3=Severe

4=Entire Muscle

Fat Symmetry:

0= asymettric fatty degeneration

1=symmetry

Edema:

0 = no edema
1= symmetrical edema
2=asymmetrical edema

Jarmula, Lusakowska et al.

Supplementary Material 3

WES Analysis of Patients 3-5

patient 3

Proband ID: MWAR023
Inferred Sex (Reported): Male (Male)
Inferred Ancestry (Reported): European (European)

Candidate Mutations

**Gene**<br>*Transcript*<br>*Genotype*[4]	**Gene Variant**[1]<br>*Transcript Change*[5]<br>*Protein Change*<br>*dbSNP*[7]	**ExAC AF**[2]<br>*Highest Pop*[6]	**ClinVar**[3]<br>*ClinVar Confidence*<br>*Stars*	**Notes**
**RYR1**<br>ENST00000359596.3<br>*het*	19:38954139 G>A<br>c.2654G>A<br>p.Arg885His<br>rs370634440	1.77e-04<br>3.66e-04	NA	
**ANO5**<br>ENST00000324559.8<br>*het*	11:22283708 G>T<br>c.1664G>T<br>p.Ser555Ile<br>rs375014127	7.42e-05<br>1.51e-04	NA	
**ANO5**<br>ENST00000324559.8<br>*het*	11:22225378 C>A<br>c.69C>A<br>p.Tyr23Ter	9.82e-06<br>1.84e-05	NA	

Footnotes:
[1]Genomic variants are based on build37/hg19. This is a filtered list of variants containing **nonsense, frameshift, essential splice** and **missense** variants.
[2]ExAC Allele Frequency (AF). For more information on ExAC please refer to *General Methodology* section.
[3]For more details on ClinVar and how confidence is rated please refer to *General Methodology* section.
[4]Genotype is either heterozygous (*het*), homozygous (*hom*) or hemizygous (*hem*) for the variant.
[5]Transcript changes are based on Ensembl.
[6]Highest allele frequency in any of the given ExAC populations (Pop).
[7]rsID if present in dbSNP version 141.
[+]Large insertion/deletions have been truncated.

Limitations:

- Analysis limited to gene list. For more information on gene list please refer to *Appendix*.
- This exome was sequenced in a research environment and results have not been clinically validated. Clinical validation of candidate causal variants should be performed prior to making a formal clinical diagnosis. Please refer to *Appendix* for coverage of this sample.
- Proband only approach used in this project and thus unable to determine phasing of two or more variants (whether in *cis* or *trans*) and also unable to determine if mutations are inherited or de novo. Determining phasing and inheritance of variants requires sequencing of parents.

Appendix - Candidate Gene List

All Rare Variants Called

**Gene**<br>*Transcript*<br>*Genotype*[4]	**Gene Variant**[1]<br>*Transcript Change*[5]<br>*Protein Change*<br>*dbSNP*[7]	**ExAC AF**[2]<br>*Highest Pop*[6]	**ClinVar**[3]<br>*ClinVar Confidence*	**Notes**
**ABHD5**<br>ENST00000458276.2<br>*hom*	3:43756577 C>CTG<br>c.773+28_773+29[4]TG	0.00e+00<br>0.00e+00	NA	
**ACADVL**<br>ENST00000543245.2<br>*het*	17:7127931 A>C<br>c.1748-30A>C	0.00e+00<br>0.00e+00	NA	
**ACADVL**<br>ENST00000543245.2<br>*het*	17:7127937 A>C<br>c.1748-24A>C	1.18e-03<br>3.85e-03	NA	
**ACADVL**<br>ENST00000543245.2<br>*het*	17:7127943 A>C<br>c.1748-18A>C	1.08e-03<br>7.52e-03	NA	
**ACADVL**<br>ENST00000543245.2<br>*het*	17:7127948 A>C<br>c.1748-13A>C	5.60e-04<br>6.34e-03	NA	
**ACADVL**<br>ENST00000543245.2<br>*het*	17:7127951 T>C<br>c.1748-10T>C	0.00e+00<br>0.00e+00	NA	
**ACADVL**<br>ENST00000543245.2<br>*het*	17:7127952 A>C<br>c.1748-9A>C	0.00e+00<br>0.00e+00	NA	
**ANO5**<br>ENST00000324559.8<br>*het*	11:22225378 C>A<br>c.69C>A<br>p.Tyr23Ter	9.82e-06<br>1.84e-05	NA	
**ANO5**<br>ENST00000324559.8<br>*het*	11:22283708 G>T<br>c.1664G>T<br>p.Ser555Ile	7.42e-05<br>1.51e-04	NA	

**Gene**<br>*Transcript*<br>*Genotype*[4]	**Gene Variant**[1]<br>*Transcript Change*[5]<br>*Protein Change*<br>*dbSNP*[7]	**ExAC AF**[2]<br>*Highest Pop*[6]	**ClinVar**[3]<br>*ClinVar Confidence*	**Notes**
	rs375014127			
**CHKB**<br>ENST00000406938.2<br>*het*	22:51021258 C>A<br>c.-48G>T	5.09e-03<br>1.69e-02	NA	
**DMD**<br>ENST00000357033.4<br>*het*	X:31950370 A>AT<br>c.6615-27delA	7.89e-04<br>3.07e-03	NA	
**DYSF**<br>ENST00000410020.3<br>*het*	2:71825707 C>T<br>c.3588C>T<br>c.3588C>T(p.=)	7.36e-03<br>1.16e-02	NA	
**FAM111B**<br>ENST00000343597.3<br>*het*	11:58892376 C>CA<br>c.807delA<br>p.Ala273HisfsTer26	4.04e-03<br>6.72e-03	NA	
**GBE1**<br>ENST00000429644.2<br>*het*	3:81719954 A>G<br>c.429+35T>C	5.34e-03<br>9.06e-03	NA	
**GMPPB**<br>ENST00000308375.6<br>*het*	3:49760503 C>G<br>c.304G>C<br>p.Asp102His<br>rs368542417	4.14e-05<br>7.54e-05	NA	
**MUSK**<br>ENST00000374448.4<br>*het*	9:113562589 T>C<br>c.1931T>C<br>p.Val644Ala<br>rs41279055	2.84e-03<br>4.22e-03	NA	
**MYBPC3**<br>ENST00000545968.1<br>*het*	11:47368208 T>G<br>c.909-13A>C	5.78e-05<br>3.29e-04	NA	
**MYH2**<br>ENST00000245503.5<br>*het*	17:10428348 C>T<br>c.4697G>A<br>p.Arg1566His<br>rs200732220	1.73e-04<br>3.00e-04	NA	
**MYH3**<br>ENST00000583535.1	17:10555926 CTTT>C<br>c.205-62_205-47delAAAAAAAAAAAA[+]	0.00e+00<br>0.00e+00	NA	

**Gene**<br>*Transcript*<br>*Genotype*[4]	**Gene Variant**[1]<br>*Transcript Change*[5]<br>*Protein Change*<br>*dbSNP*[7]	**ExAC AF**[2]<br>*Highest Pop*[6]	**ClinVar**[3]<br>*ClinVar Confidence*	**Notes**
het				
**MYH3**<br>ENST00000583535.1<br>*het*	17:10555926 CTTTT>C<br>c.205-62_205-47delAAAAAAAAAAAA[+]	0.00e+00<br>0.00e+00	NA	
**NEB**<br>ENST00000397345.3<br>*het*	2:152544120 G>C<br>c.2523+20C>G	2.53e-03<br>2.26e-02	NA	
**OPA1**<br>ENST00000361908.3<br>*het*	3:193332466 A>C<br>c.33-46A>C	0.00e+00<br>0.00e+00	NA	
**PLEC**<br>ENST00000322810.4<br>*het*	8:144992269 G>A<br>c.12131C>T<br>p.Thr4044Met<br>rs78461695	9.26e-03<br>1.30e-02	NA	
**PLEC**<br>ENST00000322810.4<br>*het*	8:144999731 C>T<br>c.4777G>A<br>p.Val1593Met<br>rs186848953	4.79e-03<br>7.23e-03	NA	
**PLEC**<br>ENST00000322810.4<br>*het*	8:145011204 A>C<br>c.789T>G<br>c.789T>G(p.=)	2.79e-03<br>4.81e-03	NA	
**RYR1**<br>ENST00000359596.3<br>*het*	19:38954139 G>A<br>c.2654G>A<br>p.Arg885His<br>rs370634440	1.77e-04<br>3.66e-04	NA	
**RYR1**<br>ENST00000359596.3<br>*het*	19:39062962 T>G<br>c.13998+52delTinsTGGGGGGGGGGG[+]	1.47e-03<br>2.81e-03	NA	
**SCN4A**<br>ENST00000435607.1<br>*het*	17:62038535 T>C<br>c.1845+17_1845+18delGAinsGGA	2.39e-03<br>4.07e-03	NA	
**SGCA**	17:48246702 G>A	7.56e-03	NA	

**Gene**<br>*Transcript*<br>*Genotype*[4]	**Gene Variant**[1]<br>*Transcript Change*[5]<br>*Protein Change*<br>*dbSNP*[7]	**ExAC AF**[2]<br>*Highest Pop*[6]	**ClinVar**[3]<br>*ClinVar Confidence*	**Notes**
ENST00000451235.2<br>*het*	c.528G>A<br>c.528G>A(p.=)	5.46e-02		
**STIM2**<br>ENST00000465503.1<br>*het*	4:27004737 T>TA<br>c.981+11_981+12delTAinsGA	4.87e-03<br>1.19e-02	NA	
**NUDT15**<br>ENST00000258662.2<br>*het*	13:48611918 A>C<br>c.36_42delAGGAGTCinsAGGAGTCGGA[+]<br>p.Val14_Gly15insGlyVal	3.43e-03<br>6.45e-03	NA	
**SYNE1**<br>ENST00000367255.5<br>*het*	6:152690074 TCA>T<br>c.9807+31_9807+32delTG	0.00e+00<br>0.00e+00	NA	
**SYNE1**<br>ENST00000367255.5<br>*het*	6:152712715 G>GAAAAAAAA<br>c.7713-13delT	6.49e-03<br>1.26e-02	NA	
**SYNE1**<br>ENST00000367255.5<br>*het*	6:152737573 C>T<br>c.5999G>A<br>p.Arg2000Lys<br>rs149146258	1.58e-03<br>5.59e-03	NA	
**TK2**<br>ENST00000451102.2<br>*het*	16:66575895 G>A<br>c.157-39C>T	2.29e-03<br>3.81e-03	NA	
**TTN**<br>ENST00000589042.1<br>*het*	2:179553730 AT>A<br>c.32095+42_32095+49delAAAAAAAA	6.92e-04<br>2.29e-03	NA	
**TTN**<br>ENST00000589042.1<br>*het*	2:179582769 C>A<br>c.24964G>T<br>p.Val8322Leu<br>rs201571580	7.54e-04<br>1.33e-03	NA	
**TTN**<br>ENST00000589042.1<br>*het*	2:179584211 A>G<br>c.23939-33T>C	0.00e+00<br>0.00e+00	NA	

**Gene**<br>*Transcript*<br>*Genotype*[4]	**Gene Variant**[1]<br>*Transcript Change*[5]<br>*Protein Change*<br>*dbSNP*[7]	**ExAC AF**[2]<br>*Highest Pop*[6]	**ClinVar**[3]<br>*ClinVar Confidence*	**Notes**
**TTN**<br>ENST00000589042.1<br>*het*	2:179596554 T>C<br>c.17048A>G<br>p.Tyr5683Cys<br>rs72648942	5.85e-03<br>8.56e-03	NA	
**VAPB**<br>ENST00000475243.1<br>*het*	20:56964367 CAGCG>C<br>c.-149_-143delCAGCGCGinsC	0.00e+00<br>0.00e+00	NA	
**VAPB**<br>ENST00000475243.1<br>*het*	20:56964373 G>C<br>c.-143G>C	0.00e+00<br>0.00e+00	NA	

Footnotes:

[1]Genomic variants are based on build37/hg19. This is a filtered list (AF <0.01) of variants containing **non-sense, frameshift, essential splice**,**missense** and **synonymous** variants.

[2]ExAC Allele Frequency (AF). For more information on ExAC please refer to *General Methodology* section.

[3]For more details on ClinVar and how confidence is rated please refer to *General Methodology* section.

[4]Genotype is either heterozygous (*het*), homozygous (*hom*) or hemizygous (*hem*) for the variant.

[5]Transcript changes are based on Ensembl.

[6]Highest allele frequency in any of the given ExAC populations (Pop).

[7]rsID if present in dbSNP version 141.

[+]Large insertion/deletions have been truncated.

Gene Coverage

Gene	Coverage[1]		Callable Sites[2] (%)		Uncallable Sites[2]	
	Cohort[3]	Sample[4]	Cohort	Sample	Cohort	Sample
ABHD5	81.52	81.30	100.00	100.00	0	0
ACADS	93.78	93.22	100.00	100.00	0	0
ACADVL	92.82	92.11	98.24	98.24	48	48
ACTA1	90.71	87.45	100.00	100.00	0	0
AGK	43.73	45.79	100.00	100.00	0	0
AGL	75.61	77.83	100.00	100.00	0	0
AGRN	77.01	70.36	99.76	95.26	17	331
ALG13	34.47	24.79	100.00	95.57	0	181
ALG14	54.88	55.44	100.00	100.00	0	0
ALG2	71.16	65.97	100.00	98.40	0	22
ANO5	75.71	78.88	100.00	100.00	0	0
ATP2A1	93.31	93.20	100.00	99.89	0	4
B3GALNT2	33.06	33.05	92.04	91.83	151	155
B3GNT1	51.99	44.29	98.60	98.21	18	23
BAG3	89.70	89.84	100.00	100.00	0	0
BIN1	83.63	84.44	100.00	100.00	0	0
C10orf2	96.55	95.47	100.00	100.00	0	0
CAPN3	90.94	92.05	100.00	100.00	0	0
CAV3	87.44	83.03	100.00	100.00	0	0
CCDC78	62.86	50.38	100.00	100.00	0	0
CFL2	68.21	69.93	100.00	100.00	0	0
CHAT	90.75	89.49	100.00	100.00	0	0
CHKB	91.98	90.22	100.00	100.00	0	0
CHRNA1	89.08	90.25	100.00	100.00	0	0
CHRNB1	91.20	91.79	96.92	94.77	56	95
CHRND	95.74	96.57	100.00	100.00	0	0
CHRNE	89.16	86.86	100.00	99.53	0	8
CHRNG	98.31	98.78	100.00	100.00	0	0
CLCN1	91.10	90.72	100.00	100.00	0	0
CNBP	62.31	61.81	100.00	100.00	0	0
CNTN1	71.74	73.90	100.00	100.00	0	0
COL12A1	44.81	44.90	100.00	99.92	0	9
COL6A1	86.80	84.53	100.00	100.00	0	0
COL6A2	87.60	84.19	100.00	100.00	0	0
COL6A3	86.48	86.27	99.59	99.29	43	74

Gene	Coverage[1]		Callable Sites[2] (%)		Uncallable Sites[2]	
	Cohort[3]	Sample[4]	Cohort	Sample	Cohort	Sample
COLQ	90.89	93.15	100.00	100.00	0	0
COX15	95.34	95.59	100.00	100.00	0	0
CPT2	87.59	85.07	100.00	99.28	0	15
CRYAB	87.02	88.57	100.00	100.00	0	0
DAG1	94.02	92.98	100.00	100.00	0	0
DES	82.38	77.44	100.00	95.28	0	75
DMD	61.42	49.05	100.00	99.69	0	40
DNAJB6	41.09	34.03	100.00	100.00	0	0
DNM2	86.07	85.49	97.80	96.43	82	133
DOK7	72.30	66.74	100.00	95.52	0	74
DOLK	93.48	92.52	100.00	100.00	0	0
DPAGT1	93.24	93.77	100.00	100.00	0	0
DPM1	62.80	67.45	93.18	89.77	74	111
DPM2	27.88	25.52	100.00	100.00	0	0
DPM3	84.43	86.07	100.00	100.00	0	0
DYSF	92.97	93.45	100.00	100.00	0	0
EMD	69.98	56.27	100.00	100.00	0	0
ENO3	97.90	97.40	100.00	100.00	0	0
ETFA	53.56	56.59	86.15	87.07	256	239
ETFB	98.62	98.06	100.00	100.00	0	0
ETFDH	58.38	57.87	100.00	100.00	0	0
FAM111B	53.15	53.90	99.42	99.11	13	20
FHL1	69.09	56.54	100.00	100.00	0	0
FKBP14	24.56	25.57	100.00	99.59	0	3
FKRP	55.29	45.65	100.00	79.27	0	312
FKTN	66.04	70.62	97.07	97.07	56	56
FLNC	94.92	94.24	100.00	100.00	0	0
GAA	84.62	84.02	100.00	100.00	0	0
GBE1	27.25	27.40	100.00	100.00	0	0
GFPT1	73.05	76.11	100.00	100.00	0	0
GMPPB	63.96	54.27	100.00	100.00	0	0
GNE	83.70	85.63	100.00	100.00	0	0
GYG1	77.76	77.41	100.00	100.00	0	0
GYS1	88.92	88.40	100.00	100.00	0	0
HNRNPDL	39.25	37.67	90.00	87.14	140	180
IGHMBP2	90.87	92.15	100.00	100.00	0	0

Gene	Coverage[1]		Callable Sites[2] (%)		Uncallable Sites[2]	
	Cohort[3]	Sample[4]	Cohort	Sample	Cohort	Sample
ISCU	69.30	69.53	100.00	93.99	0	53
ISPD	32.81	31.05	82.16	82.03	277	279
ITGA7	90.12	90.11	100.00	100.00	0	0
KBTBD13	49.11	42.71	96.77	84.07	45	222
KCNJ2	99.05	100.00	100.00	100.00	0	0
KLHL40	71.73	67.16	100.00	100.00	0	0
KLHL41	53.63	53.14	100.00	100.00	0	0
KLHL9	80.33	79.18	100.00	100.00	0	0
LAMA2	85.80	88.22	100.00	100.00	0	0
LAMB2	97.05	96.86	100.00	100.00	0	0
LAMP2	50.06	38.73	100.00	97.45	0	44
LARGE	90.15	90.99	100.00	100.00	0	0
LDB3	89.95	89.63	100.00	100.00	0	0
LDHA	70.61	69.39	100.00	100.00	0	0
LIMS2	55.94	54.38	90.90	90.90	134	134
LMNA	88.12	86.87	100.00	100.00	0	0
LPIN1	76.22	76.58	100.00	100.00	0	0
LRP4	95.67	95.83	100.00	100.00	0	0
MATR3	67.55	68.16	96.08	95.54	123	140
MEGF10	46.71	47.98	100.00	99.95	0	2
MSTN	72.08	73.08	100.00	100.00	0	0
MTM1	54.57	43.72	100.00	100.00	0	0
MTMR14	85.21	83.43	100.00	99.81	0	5
MTTP	80.04	82.94	100.00	100.00	0	0
MUSK	52.22	53.35	100.00	99.51	0	15
MYBPC3	50.86	48.70	99.47	99.31	24	31
MYF6	90.16	91.40	100.00	100.00	0	0
MYH14	68.18	67.30	100.00	99.05	0	66
MYH2	91.34	93.88	100.00	100.00	0	0
MYH3	95.96	97.18	100.00	100.00	0	0
MYH7	97.49	97.92	100.00	100.00	0	0
MYOT	75.98	78.45	100.00	100.00	0	0
NEB	45.79	46.59	87.75	86.29	3589	4018
OPA1	62.12	65.98	100.00	100.00	0	0
ORAI1	83.27	84.74	89.78	88.30	97	111
PABPN1	68.54	63.35	92.39	83.38	81	177

Gene	Coverage[1]		Callable Sites[2] (%)		Uncallable Sites[2]	
	Cohort[3]	Sample[4]	Cohort	Sample	Cohort	Sample
PFKM	85.41	85.32	100.00	99.97	0	1
PGAM2	99.17	99.06	100.00	100.00	0	0
PGK1	77.92	59.77	100.00	100.00	0	0
PGM1	91.24	94.24	100.00	100.00	0	0
PHKA1	66.18	51.71	100.00	100.00	0	0
PLEC	85.21	76.54	99.77	99.20	36	127
PNPLA2	78.73	69.18	99.59	98.88	7	19
POLG	91.70	89.56	99.00	96.69	42	139
POLG2	67.30	68.15	100.00	100.00	0	0
POMGNT1	90.97	91.36	100.00	100.00	0	0
POMGNT2	82.97	80.52	100.00	100.00	0	0
POMK	81.71	81.72	100.00	100.00	0	0
POMT1	93.06	93.05	100.00	100.00	0	0
POMT2	90.34	91.14	100.00	99.97	0	1
PREPL	45.05	45.88	99.96	100.00	1	0
PRKAG2	75.44	76.52	100.00	100.00	0	0
PTPLA	22.56	21.57	97.86	91.27	27	110
PTRF	84.71	82.15	99.34	96.12	8	47
PYGM	98.79	98.76	100.00	100.00	0	0
RAPSN	84.64	84.62	100.00	100.00	0	0
RRM2B	68.60	72.14	91.25	91.25	141	141
RYR1	91.80	91.47	97.64	96.58	412	598
SCN4A	74.92	74.53	100.00	100.00	0	0
SEPN1	80.43	80.45	89.90	89.90	205	205
SGCA	88.28	87.37	100.00	100.00	0	0
SGCB	68.02	74.20	99.40	95.44	7	53
SGCD	37.72	37.98	100.00	100.00	0	0
SGCG	86.15	91.83	100.00	100.00	0	0
SIL1	94.85	96.40	100.00	100.00	0	0
SLC22A5	92.52	90.99	100.00	100.00	0	0
SLC25A20	93.29	93.48	100.00	100.00	0	0
SLC25A4	76.10	78.28	100.00	100.00	0	0
SLC52A3	93.42	93.00	100.00	100.00	0	0
SMCHD1	24.46	25.14	98.56	96.24	102	266
STAC3	60.18	56.46	100.00	99.85	0	2
STIM1	86.56	85.92	100.00	100.00	0	0

Gene	Coverage[1]		Callable Sites[2] (%)		Uncallable Sites[2]	
	Cohort[3]	Sample[4]	Cohort	Sample	Cohort	Sample
STIM2	43.79	43.42	98.34	96.54	47	98
SUCLA2	70.70	76.50	100.00	100.00	0	0
SYNE1	84.25	86.32	100.00	100.00	0	0
SYNE2	74.71	77.40	99.98	99.99	4	3
TARDBP	67.48	66.98	89.56	89.56	165	165
TAZ	81.92	69.44	100.00	100.00	0	0
TCAP	83.34	84.49	100.00	100.00	0	0
TIA1	32.56	34.64	100.00	100.00	0	0
TK2	77.13	76.14	94.97	92.17	68	106
TMEM43	74.28	73.08	100.00	100.00	0	0
TMEM5	31.07	32.61	100.00	96.81	0	48
TNNI2	93.91	91.76	100.00	100.00	0	0
TNNT1	69.98	71.63	95.35	89.02	64	151
TNNT3	99.16	99.23	100.00	100.00	0	0
TNPO3	44.98	45.42	100.00	99.82	0	6
TOR1AIP1	51.16	49.69	97.51	95.44	55	101
TPM2	90.27	89.60	100.00	100.00	0	0
TPM3	96.87	98.21	100.00	100.00	0	0
TRAPPC11	37.43	37.19	100.00	100.00	0	0
TRIM32	94.96	96.18	100.00	100.00	0	0
TTN	58.05	56.11	98.35	97.48	2002	3061
UBA1	85.50	76.31	99.98	98.23	1	73
VAPB	80.46	82.12	100.00	100.00	0	0
VCP	98.26	98.18	100.00	100.00	0	0
VMA21	41.32	33.88	100.00	100.00	0	0
YARS2	83.53	87.22	100.00	100.00	0	0

Notes:

[1]Average per-base coverage for gene. Genes that are not completely callable are highlighted in **pink**. For more information on how coverage was calculated, please refer to *General Methodology* section.

[2]The percent of sites that are considered callable for this gene. For more information on how callability is determined, please refer to *General Methodology* section.

[3]The average for samples included in the cohort.

[4]The metric for the sample in this report.

patient 4

Proband ID: MWAR022
Inferred Sex (Reported): Female (Female)
Inferred Ancestry (Reported): European (European)

Candidate Mutations

**Gene**<br>*Transcript*<br>*Genotype*[4]	**Gene Variant**[1]<br>*Transcript Change*[5]<br>*Protein Change*<br>*dbSNP*[7]	**ExAC AF**[2]<br>*Highest Pop*[6]	**ClinVar**[3]<br>*ClinVar Confidence*<br>*Stars*	**Notes**
**ANO5**<br>ENST00000324559.8<br>*het*	11:22248879 A>T<br>c.395A>T<br>p.Lys132Met	0.00e+00<br>0.00e+00	NA	
**ANO5**<br>ENST00000324559.8<br>*het*	11:22301090 C>G<br>c.2521C>G<br>p.His841Asp	1.67e-05<br>3.04e-05	NA	

Footnotes:
[1]Genomic variants are based on build37/hg19. This is a filtered list of variants containing **nonsense, frameshift, essential splice** and **missense** variants.
[2]ExAC Allele Frequency (AF). For more information on ExAC please refer to *General Methodology* section.
[3]For more details on ClinVar and how confidence is rated please refer to *General Methodology* section.
[4]Genotype is either heterozygous (*het*), homozygous (*hom*) or hemizygous (*hem*) for the variant.
[5]Transcript changes are based on Ensembl.
[6]Highest allele frequency in any of the given ExAC populations (Pop).
[7]rsID if present in dbSNP version 141.
[+]Large insertion/deletions have been truncated.

Limitations:

- Analysis limited to gene list. For more information on gene list please refer to *Appendix*.
- This exome was sequenced in a research environment and results have not been clinically validated. Clinical validation of candidate causal variants should be performed prior to making a formal clinical diagnosis. Please refer to *Appendix* for coverage of this sample.
- Proband only approach used in this project and thus unable to determine phasing of two or more variants (whether in *cis* or *trans*) and also unable to determine if mutations are inherited or de novo. Determining phasing and inheritance of variants requires sequencing of parents.

Appendix - Candidate Gene List

All Rare Variants Called

**Gene**<br>*Transcript*<br>*Genotype*[4]	**Gene Variant**[1]<br>*Transcript Change*[5]<br>*Protein Change*<br>*dbSNP*[7]	**ExAC AF**[2]<br>*Highest Pop*[6]	**ClinVar**[3]<br>*ClinVar Confidence*	**Notes**
**ABHD5**<br>ENST00000458276.2<br>*het*	3:43756577 C>CTG<br>c.773+28_773+29[4]TG	0.00e+00<br>0.00e+00	NA	
**ABHD5**<br>ENST00000458276.2<br>*het*	3:43756579 G>C<br>c.773+29G>C	0.00e+00<br>0.00e+00	NA	
**ACADVL**<br>ENST00000543245.2<br>*het*	17:7127931 A>ACCCCCC<br>c.1748-30delAinsACCCCCCC	0.00e+00<br>0.00e+00	NA	
**ACADVL**<br>ENST00000543245.2<br>*het*	17:7127937 A>C<br>c.1748-24A>C	1.18e-03<br>3.85e-03	NA	
**ACADVL**<br>ENST00000543245.2<br>*het*	17:7127943 A>C<br>c.1748-18A>C	1.08e-03<br>7.52e-03	NA	
**ACADVL**<br>ENST00000543245.2<br>*het*	17:7127948 A>C<br>c.1748-13A>C	5.60e-04<br>6.34e-03	NA	
**AGRN**<br>ENST00000379370.2<br>*het*	1:981340 C>T<br>c.2681-4C>T	8.11e-04<br>1.30e-03	NA	
**ANO5**<br>ENST00000324559.8<br>*het*	11:22248879 A>T<br>c.395A>T<br>p.Lys132Met	0.00e+00<br>0.00e+00	NA	
**ANO5**<br>ENST00000324559.8<br>*het*	11:22301090 C>G<br>c.2521C>G<br>p.His841Asp	1.67e-05<br>3.04e-05	NA	

**Gene**<br>*Transcript*<br>*Genotype*[4]	**Gene Variant**[1]<br>*Transcript Change*[5]<br>*Protein Change*<br>*dbSNP*[7]	**ExAC AF**[2]<br>*Highest Pop*[6]	**ClinVar**[3]<br>*ClinVar Confidence*	**Notes**
**AR**<br>ENST00000374690.3<br>*het*	X:66765158 TGCA>T<br>c.171_182delGCAGCAGCAGCAinsGCA[+]<br>p.Gln61dup	3.15e-03<br>6.12e-03	NA	
**AR**<br>ENST00000374690.3<br>*het*	X:66765161 A>T<br>c.173A>T<br>p.Gln58Leu<br>rs200185441	0.00e+00<br>0.00e+00	NA	
**BAG3**<br>ENST00000369085.3<br>*het*	10:121431904 C>T<br>c.645C>T<br>c.645C>T(p.=)	1.74e-04<br>4.54e-04	NA	
**C10orf2**<br>ENST00000311916.2<br>*het*	10:102753257 G>A<br>c.2045G>A<br>p.Arg682His<br>rs182559752	6.46e-04<br>2.97e-03	NA	
**COL12A1**<br>ENST00000322507.8<br>*het*	6:75833783 C>T<br>c.6752G>A<br>p.Arg2251His<br>rs151324784	3.73e-04<br>5.86e-04	NA	
**COL12A1**<br>ENST00000322507.8<br>*het*	6:75890913 T>C<br>c.1906A>G<br>p.Lys636Glu	8.37e-06<br>1.05e-04	NA	
**DYSF**<br>ENST00000410020.3<br>*het*	2:71740897 C>A<br>c.605C>A<br>p.Ala202Glu<br>rs34999029	9.81e-03<br>1.48e-02	NA	
**DYSF**<br>ENST00000410020.3<br>*het*	2:71762387 T>C<br>c.1450-11T>C	0.00e+00<br>0.00e+00	NA	
**FLNC**<br>ENST00000325888.8<br>*het*	7:128485240 C>T<br>c.3721C>T<br>p.Arg1241Cys<br>rs146953558	6.29e-03<br>1.08e-02	NA	
**LAMA2**<br>ENST00000421865.2	6:129601217 C>T<br>c.2462C>T	2.03e-03<br>3.64e-03	NA	

**Gene**<br>*Transcript*<br>*Genotype*[4]	**Gene Variant**[1]<br>*Transcript Change*[5]<br>*Protein Change*<br>*dbSNP*[7]	**ExAC AF**[2]<br>*Highest Pop*[6]	**ClinVar**[3]<br>*ClinVar Confidence*	**Notes**
het	p.Thr821Met<br>rs117422805			
**MUSK**<br>ENST00000374439.1<br>*het*	9:113468417 C>T<br>c.336C>T<br>c.336C>T(p.=)	2.69e-03<br>4.64e-03	NA	
**MYH14**<br>ENST00000601313.1<br>*het*	19:50770307 T>G<br>c.2826+42T>G	0.00e+00<br>0.00e+00	NA	
**MYH3**<br>ENST00000583535.1<br>*het*	17:10555926 CTT>C<br>c.205-62_205-47delAAAAAAAAAAAA+	0.00e+00<br>0.00e+00	NA	
**MYH3**<br>ENST00000583535.1<br>*het*	17:10555926 CTTTTTTTTT+>C<br>c.205-63_205-47delAAAAAAAAAAAA+	3.25e-03<br>7.84e-03	NA	
**MYH3**<br>ENST00000583535.1<br>*het*	17:10555927 T>C<br>c.205-47A>G	0.00e+00<br>0.00e+00	NA	
**MYH3**<br>ENST00000583535.1<br>*het*	17:10555929 T>C<br>c.205-49A>G	0.00e+00<br>0.00e+00	NA	
**NEB**<br>ENST00000397345.3<br>*het*	2:152359276 ATT>A<br>c.23928+28_23928+30delAAA	6.06e-03<br>9.90e-03	NA	
**NEB**<br>ENST00000397345.3<br>*het*	2:152370963 A>G<br>c.23017-21T>C	6.20e-03<br>1.41e-02	NA	
**NEB**<br>ENST00000397345.3<br>*het*	2:152410513 C>T<br>c.19455G>A<br>c.19455G>A(p.=)	3.39e-03<br>1.33e-02	NA	
**PHKA1**	X:71813193 T>TC	0.00e+00	NA	

**Gene**<br>*Transcript*<br>*Genotype*[4]	**Gene Variant**[1]<br>*Transcript Change*[5]<br>*Protein Change*<br>*dbSNP*[7]	**ExAC AF**[2]<br>*Highest Pop*[6]	**ClinVar**[3]<br>*ClinVar Confidence*	**Notes**
ENST00000373542.4<br>*het*	c.3073-69delAinsGA	0.00e+00		
**PLEC**<br>ENST00000322810.4<br>*het*	8:144997370 G>T<br>c.7138C>A<br>p.Leu2380Ile<br>rs372627763	1.29e-04<br>2.35e-04	NA	
**PLEC**<br>ENST00000322810.4<br>*het*	8:145011889 G>C<br>c.675+430C>G	1.87e-04<br>7.23e-04	NA	
**PREPL**<br>ENST00000409936.1<br>*het*	2:44559583 G>A<br>c.1353+15C>T	6.82e-03<br>1.03e-02	NA	
**RYR1**<br>ENST00000359596.3<br>*het*	19:38968521 T>G<br>c.4454+11T>G	0.00e+00<br>0.00e+00	NA	
**RYR1**<br>ENST00000359596.3<br>*het*	19:38993391 TGGGGCAGGG+>T<br>c.7835+25_7835+42delGGGGCAGGGG+	2.53e-04<br>2.07e-03	NA	
**RYR1**<br>ENST00000359596.3<br>*het*	19:39056020 GGGC>G<br>c.13047_13049delGGC<br>p.Ala4352del	0.00e+00<br>0.00e+00	NA	
**SYNE1**<br>ENST00000367255.5<br>*het*	6:152712715 G>GA<br>c.7713-13delT	0.00e+00<br>0.00e+00	NA	
**SYNE2**<br>ENST00000358025.3<br>*het*	14:64443259 T>C<br>c.1129-22N>C	0.00e+00<br>0.00e+00	NA	
**SYNE2**<br>ENST00000358025.3<br>*het*	14:64453292 T>C<br>c.2270T>C<br>p.Leu757Ser<br>rs200319405	4.14e-04<br>7.04e-04	NA	

**Gene**<br>*Transcript*<br>*Genotype*[4]	**Gene Variant**[1]<br>*Transcript Change*[5]<br>*Protein Change*<br>*dbSNP*[7]	**ExAC AF**[2]<br>*Highest Pop*[6]	**ClinVar**[3]<br>*ClinVar Confidence*	**Notes**
**SYNE2**<br>ENST00000358025.3<br>*het*	14:64494308 C>G<br>c.6511C>G<br>p.Leu2171Val<br>rs199743242	4.23e-04<br>7.20e-04	NA	
**TNNT1**<br>ENST00000588981.1<br>*hom*	19:55646893 C>CT<br>c.612-1325_612-1322delAAAA	0.00e+00<br>0.00e+00	NA	
**TTN**<br>ENST00000589042.1<br>*het*	2:179478597 C>A<br>c.49413G>T<br>p.Trp16471Cys<br>rs202094100	3.92e-04<br>6.32e-04	NA	
**TTN**<br>ENST00000589042.1<br>*het*	2:179478746 C>CT<br>c.49345+32delA	7.50e-03<br>2.99e-02	NA	
**TTN**<br>ENST00000589042.1<br>*het*	2:179519303 T>C<br>c.38207-42A>G	3.33e-03<br>1.94e-02	NA	
**TTN**<br>ENST00000589042.1<br>*het*	2:179544076 C>T<br>c.33732G>A<br>c.33732G>A(p.=)	2.58e-04<br>4.24e-04	NA	
**TTN**<br>ENST00000589042.1<br>*het*	2:179553730 ATT>A<br>c.32095+42_32095+49delAAAAAAAA	1.41e-03<br>3.44e-03	NA	
**TTN**<br><br>*het*	2:179553755 G>T	5.93e-04<br>7.97e-04	NA	
**UBA1**<br>ENST00000335972.6<br>*het*	X:47063147 T>TA<br>c.1575+52delTinsTA	0.00e+00<br>0.00e+00	NA	
**YARS2**<br>ENST00000324868.8<br>*het*	12:32905700 G>A<br>c.947+1152C>T	9.33e-04<br>1.30e-03	NA	

**Gene**<br>*Transcript*<br>*Genotype*[4]	**Gene Variant**[1]<br>*Transcript Change*[5]<br>*Protein Change*<br>*dbSNP*[7]	**ExAC AF**[2]<br>*Highest Pop*[6]	**ClinVar**[3]<br>*ClinVar Confidence*	**Notes**
**YARS2**<br>ENST00000324868.8<br>*het*	12:32905701 ATTATC>A<br>c.947+1146_947+1150delGATAA	3.20e-04<br>4.54e-04	NA	

Footnotes:
[1]Genomic variants are based on build37/hg19. This is a filtered list (AF <0.01) of variants containing **non-sense, frameshift, essential splice**,**missense** and **synonymous** variants.
[2]ExAC Allele Frequency (AF). For more information on ExAC please refer to *General Methodology* section.
[3]For more details on ClinVar and how confidence is rated please refer to *General Methodology* section.
[4]Genotype is either heterozygous (*het*), homozygous (*hom*) or hemizygous (*hem*) for the variant.
[5]Transcript changes are based on Ensembl.
[6]Highest allele frequency in any of the given ExAC populations (Pop).
[7]rsID if present in dbSNP version 141.
[+]Large insertion/deletions have been truncated.

Gene Coverage

Gene	Coverage[1]		Callable Sites[2] (%)		Uncallable Sites[2]	
	Cohort[3]	Sample[4]	Cohort	Sample	Cohort	Sample
ABHD5	81.52	78.25	100.00	100.00	0	0
ACADS	93.78	96.85	100.00	100.00	0	0
ACADVL	92.82	95.61	98.24	98.24	48	48
ACTA1	90.71	99.47	100.00	100.00	0	0
AGK	43.73	36.57	100.00	96.81	0	53
AGL	75.61	70.07	100.00	100.00	0	0
AGRN	77.01	87.27	99.76	98.63	17	96
ALG13	34.47	43.21	100.00	99.93	0	3
ALG14	54.88	43.68	100.00	100.00	0	0
ALG2	71.16	79.95	100.00	100.00	0	0
ANO5	75.71	73.68	100.00	100.00	0	0
ATP2A1	93.31	95.29	100.00	97.80	0	79
B3GALNT2	33.06	25.98	92.04	87.82	151	231
B3GNT1	51.99	69.88	98.60	100.00	18	0
BAG3	89.70	91.60	100.00	100.00	0	0
BIN1	83.63	88.52	100.00	100.00	0	0
C10orf2	96.55	99.98	100.00	100.00	0	0
CAPN3	90.94	92.75	100.00	100.00	0	0
CAV3	87.44	94.75	100.00	100.00	0	0
CCDC78	62.86	80.17	100.00	100.00	0	0
CFL2	68.21	81.28	100.00	100.00	0	0
CHAT	90.75	93.83	100.00	100.00	0	0
CHKB	91.98	91.01	100.00	100.00	0	0
CHRNA1	89.08	93.82	100.00	100.00	0	0
CHRNB1	91.20	93.97	96.92	100.00	56	0
CHRND	95.74	95.79	100.00	100.00	0	0
CHRNE	89.16	95.84	100.00	100.00	0	0
CHRNG	98.31	99.93	100.00	100.00	0	0
CLCN1	91.10	93.04	100.00	100.00	0	0
CNBP	62.31	71.95	100.00	100.00	0	0
CNTN1	71.74	65.19	100.00	100.00	0	0
COL12A1	44.81	40.40	100.00	99.18	0	88
COL6A1	86.80	94.23	100.00	100.00	0	0
COL6A2	87.60	93.69	100.00	100.00	0	0
COL6A3	86.48	93.00	99.59	99.59	43	43

Gene	Coverage[1]		Callable Sites[2] (%)		Uncallable Sites[2]	
	Cohort[3]	Sample[4]	Cohort	Sample	Cohort	Sample
COLQ	90.89	93.70	100.00	100.00	0	0
COX15	95.34	96.71	100.00	100.00	0	0
CPT2	87.59	93.28	100.00	100.00	0	0
CRYAB	87.02	92.10	100.00	100.00	0	0
DAG1	94.02	99.91	100.00	100.00	0	0
DES	82.38	91.62	100.00	100.00	0	0
DMD	61.42	74.45	100.00	100.00	0	0
DNAJB6	41.09	34.42	100.00	100.00	0	0
DNM2	86.07	86.39	97.80	98.01	82	74
DOK7	72.30	76.61	100.00	97.94	0	34
DOLK	93.48	99.98	100.00	100.00	0	0
DPAGT1	93.24	93.86	100.00	100.00	0	0
DPM1	62.80	56.38	93.18	95.94	74	44
DPM2	27.88	24.42	100.00	100.00	0	0
DPM3	84.43	94.47	100.00	100.00	0	0
DYSF	92.97	93.56	100.00	100.00	0	0
EMD	69.98	91.29	100.00	100.00	0	0
ENO3	97.90	99.85	100.00	100.00	0	0
ETFA	53.56	45.34	86.15	86.09	256	257
ETFB	98.62	98.90	100.00	100.00	0	0
ETFDH	58.38	54.95	100.00	100.00	0	0
FAM111B	53.15	61.09	99.42	98.48	13	34
FHL1	69.09	85.33	100.00	100.00	0	0
FKBP14	24.56	18.39	100.00	83.60	0	121
FKRP	55.29	62.28	100.00	97.01	0	45
FKTN	66.04	57.09	97.07	100.00	56	0
FLNC	94.92	99.02	100.00	100.00	0	0
GAA	84.62	87.87	100.00	100.00	0	0
GBE1	27.25	20.10	100.00	93.03	0	172
GFPT1	73.05	68.53	100.00	100.00	0	0
GMPPB	63.96	77.47	100.00	100.00	0	0
GNE	83.70	78.87	100.00	100.00	0	0
GYG1	77.76	81.74	100.00	100.00	0	0
GYS1	88.92	93.14	100.00	100.00	0	0
HNRNPDL	39.25	35.97	90.00	91.14	140	124
IGHMBP2	90.87	96.05	100.00	100.00	0	0

Gene	Coverage[1]		Callable Sites[2] (%)		Uncallable Sites[2]	
	Cohort[3]	Sample[4]	Cohort	Sample	Cohort	Sample
ISCU	69.30	65.59	100.00	98.19	0	16
ISPD	32.81	26.93	82.16	78.49	277	334
ITGA7	90.12	92.14	100.00	100.00	0	0
KBTBD13	49.11	56.82	96.77	99.35	45	9
KCNJ2	99.05	100.00	100.00	100.00	0	0
KLHL40	71.73	85.65	100.00	100.00	0	0
KLHL41	53.63	61.95	100.00	100.00	0	0
KLHL9	80.33	93.22	100.00	100.00	0	0
LAMA2	85.80	82.89	100.00	100.00	0	0
LAMB2	97.05	99.84	100.00	100.00	0	0
LAMP2	50.06	60.68	100.00	99.83	0	3
LARGE	90.15	89.95	100.00	100.00	0	0
LDB3	89.95	95.63	100.00	100.00	0	0
LDHA	70.61	61.58	100.00	100.00	0	0
LIMS2	55.94	59.56	90.90	90.90	134	134
LMNA	88.12	90.58	100.00	100.00	0	0
LPIN1	76.22	78.12	100.00	100.00	0	0
LRP4	95.67	96.78	100.00	99.68	0	21
MATR3	67.55	75.30	96.08	95.54	123	140
MEGF10	46.71	39.46	100.00	100.00	0	0
MSTN	72.08	78.39	100.00	100.00	0	0
MTM1	54.57	62.60	100.00	100.00	0	0
MTMR14	85.21	84.39	100.00	100.00	0	0
MTTP	80.04	77.97	100.00	100.00	0	0
MUSK	52.22	52.70	100.00	99.12	0	27
MYBPC3	50.86	55.81	99.47	100.00	24	0
MYF6	90.16	100.00	100.00	100.00	0	0
MYH14	68.18	71.21	100.00	100.00	0	0
MYH2	91.34	95.18	100.00	100.00	0	0
MYH3	95.96	97.77	100.00	99.98	0	1
MYH7	97.49	99.08	100.00	100.00	0	0
MYOT	75.98	70.87	100.00	100.00	0	0
NEB	45.79	44.71	87.75	90.29	3589	2844
OPA1	62.12	57.56	100.00	100.00	0	0
ORAI1	83.27	80.32	89.78	86.30	97	130
PABPN1	68.54	67.89	92.39	88.92	81	118

Gene	Coverage[1]		Callable Sites[2] (%)		Uncallable Sites[2]	
	Cohort[3]	Sample[4]	Cohort	Sample	Cohort	Sample
PFKM	85.41	84.15	100.00	100.00	0	0
PGAM2	99.17	100.00	100.00	100.00	0	0
PGK1	77.92	96.76	100.00	100.00	0	0
PGM1	91.24	92.32	100.00	100.00	0	0
PHKA1	66.18	80.50	100.00	100.00	0	0
PLEC	85.21	94.42	99.77	99.77	36	36
PNPLA2	78.73	81.78	99.59	96.28	7	63
POLG	91.70	93.95	99.00	99.00	42	42
POLG2	67.30	71.44	100.00	100.00	0	0
POMGNT1	90.97	93.94	100.00	100.00	0	0
POMGNT2	82.97	93.85	100.00	100.00	0	0
POMK	81.71	87.75	100.00	100.00	0	0
POMT1	93.06	93.43	100.00	100.00	0	0
POMT2	90.34	92.13	100.00	100.00	0	0
PREPL	45.05	38.89	99.96	99.96	1	1
PRKAG2	75.44	71.79	100.00	99.91	0	2
PTPLA	22.56	16.63	97.86	88.89	27	140
PTRF	84.71	91.83	99.34	100.00	8	0
PYGM	98.79	99.48	100.00	100.00	0	0
RAPSN	84.64	87.33	100.00	100.00	0	0
RRM2B	68.60	66.76	91.25	91.25	141	141
RYR1	91.80	93.54	97.64	97.71	412	401
SCN4A	74.92	78.25	100.00	100.00	0	0
SEPN1	80.43	79.99	89.90	90.00	205	203
SGCA	88.28	93.67	100.00	100.00	0	0
SGCB	68.02	55.38	99.40	95.44	7	53
SGCD	37.72	28.58	100.00	100.00	0	0
SGCG	86.15	81.36	100.00	100.00	0	0
SIL1	94.85	96.56	100.00	100.00	0	0
SLC22A5	92.52	98.08	100.00	100.00	0	0
SLC25A20	93.29	92.62	100.00	100.00	0	0
SLC25A4	76.10	81.52	100.00	100.00	0	0
SLC52A3	93.42	98.85	100.00	100.00	0	0
SMCHD1	24.46	18.93	98.56	91.29	102	616
STAC3	60.18	64.21	100.00	100.00	0	0
STIM1	86.56	88.71	100.00	100.00	0	0

Gene	Coverage[1]		Callable Sites[2] (%)		Uncallable Sites[2]	
	Cohort[3]	Sample[4]	Cohort	Sample	Cohort	Sample
STIM2	43.79	36.33	98.34	90.21	47	277
SUCLA2	70.70	57.72	100.00	100.00	0	0
SYNE1	84.25	83.72	100.00	99.76	0	71
SYNE2	74.71	76.64	99.98	99.40	4	146
TARDBP	67.48	68.96	89.56	89.56	165	165
TAZ	81.92	98.97	100.00	100.00	0	0
TCAP	83.34	99.08	100.00	100.00	0	0
TIA1	32.56	25.08	100.00	100.00	0	0
TK2	77.13	77.10	94.97	95.71	68	58
TMEM43	74.28	72.68	100.00	100.00	0	0
TMEM5	31.07	25.29	100.00	99.87	0	2
TNNI2	93.91	96.09	100.00	100.00	0	0
TNNT1	69.98	71.29	95.35	98.25	64	24
TNNT3	99.16	98.90	100.00	100.00	0	0
TNPO3	44.98	40.35	100.00	99.97	0	1
TOR1AIP1	51.16	58.75	97.51	100.00	55	0
TPM2	90.27	89.12	100.00	100.00	0	0
TPM3	96.87	96.10	100.00	100.00	0	0
TRAPPC11	37.43	29.89	100.00	95.58	0	177
TRIM32	94.96	99.52	100.00	100.00	0	0
TTN	58.05	63.24	98.35	99.37	2002	766
UBA1	85.50	93.45	99.98	100.00	1	0
VAPB	80.46	74.41	100.00	100.00	0	0
VCP	98.26	98.78	100.00	100.00	0	0
VMA21	41.32	58.40	100.00	100.00	0	0
YARS2	83.53	86.56	100.00	100.00	0	0

Notes:
[1]Average per-base coverage for gene. Genes that are not completely callable are highlighted in pink. For more information on how coverage was calculated, please refer to *General Methodology* section.
[2]The percent of sites that are considered callable for this gene. For more information on how callability is determined, please refer to *General Methodology* section.
[3]The average for samples included in the cohort.
[4]The metric for the sample in this report.

patient 5

Proband ID: MWAR110
Inferred Sex (Reported): Female (Female)
Inferred Ancestry (Reported): European (European)

Candidate Mutations

**Gene**<br>*Transcript*<br>*Genotype*[4]	**Gene Variant**[1]<br>*Transcript Change*[5]<br>*Protein Change*<br>*dbSNP*[7]	**ExAC AF**[2]<br>*Highest Pop*[6]	**ClinVar**[3]<br>*ClinVar Confidence*<br>*Stars*	**Notes**
**ANO5**<br>ENST00000324559.8<br>*het*	11:22291971 A>G<br>c.2012A>G<br>p.Tyr671Cys	8.24e-06<br>1.50e-05	NA	
**ANO5**<br>ENST00000324559.8<br>*het*	11:22242646 C>CA<br>c.191dupA<br>p.Asn64LysfsTer15	1.03e-03<br>1.71e-03	NA	

Footnotes:
[1]Genomic variants are based on build37/hg19. This is a filtered list of variants containing **nonsense, frameshift, essential splice** and **missense** variants.
[2]ExAC Allele Frequency (AF). For more information on ExAC please refer to *General Methodology* section.
[3]For more details on ClinVar and how confidence is rated please refer to *General Methodology* section.
[4]Genotype is either heterozygous (*het*), homozygous (*hom*) or hemizygous (*hem*) for the variant.
[5]Transcript changes are based on Ensembl.
[6]Highest allele frequency in any of the given ExAC populations (Pop).
[7]rsID if present in dbSNP version 141.
[+]Large insertion/deletions have been truncated.

Limitations:

- Analysis limited to gene list. For more information on gene list please refer to *Appendix*.
- This exome was sequenced in a research environment and results have not been clinically validated. Clinical validation of candidate causal variants should be performed prior to making a formal clinical diagnosis. Please refer to *Appendix* for coverage of this sample.
- Proband only approach used in this project and thus unable to determine phasing of two or more variants (whether in *cis* or *trans*) and also unable to determine if mutations are inherited or de novo. Determining phasing and inheritance of variants requires sequencing of parents.

Appendix - Candidate Gene List

All Rare Variants Called

**Gene**<br>*Transcript*<br>*Genotype*[4]	**Gene Variant**[1]<br>*Transcript Change*[5]<br>*Protein Change*<br>*dbSNP*[7]	**ExAC AF**[2]<br>*Highest Pop*[6]	**ClinVar**[3]<br>*ClinVar Confidence*	**Notes**
**ABHD5**<br>ENST00000458276.2<br>*het*	3:43756577 C>CTG<br>c.773+61_773+62dupGT	0.00e+00<br>0.00e+00	NA	
**ACADVL**<br>ENST00000543245.2<br>*het*	17:7127943 A>C<br>c.1748-18A>C	1.08e-03<br>7.52e-03	NA	
**ACADVL**<br>ENST00000543245.2<br>*het*	17:7127948 A>C<br>c.1748-13A>C	5.60e-04<br>6.34e-03	NA	
**ACADVL**<br>ENST00000543245.2<br>*het*	17:7127951 T>C<br>c.1748-10T>C	0.00e+00<br>0.00e+00	NA	
**AGRN**<br>ENST00000379370.2<br>*het*	1:982302 C>A<br>c.3353C>A<br>p.Thr1118Lys<br>rs149159118	2.15e-03<br>1.43e-02	NA	
**ALG13**<br>ENST00000394780.3<br>*het*	X:110987996 ACCT>A<br>c.2833_2835delCCT<br>p.Pro945del	7.11e-03<br>1.55e-02	NA	
**ANO5**<br>ENST00000324559.8<br>*het*	11:22242646 C>CA<br>c.191dupA<br>p.Asn64LysfsTer15	1.03e-03<br>1.71e-03	NA	
**ANO5**<br>ENST00000324559.8<br>*het*	11:22291971 A>G<br>c.2012A>G<br>p.Tyr671Cys	8.24e-06<br>1.50e-05	NA	
**AR**<br>ENST00000374690.3<br>*het*	X:66765158 TGCA>T<br>c.237_239delGCA<br>p.Gln80del	3.15e-03<br>6.12e-03	NA	

**Gene**<br>*Transcript*<br>*Genotype*[4]	**Gene Variant**[1]<br>*Transcript Change*[5]<br>*Protein Change*<br>*dbSNP*[7]	**ExAC AF**[2]<br>*Highest Pop*[6]	**ClinVar**[3]<br>*ClinVar Confidence*	**Notes**
**CAPN3**<br>ENST00000397163.3<br>*het*	15:42684803 G>A<br>c.946-34G>A	1.39e-04<br>3.34e-04	NA	
**CAPN3**<br>ENST00000397163.3<br>*het*	15:42695248 C>A<br>c.1745+48C>A	1.89e-04<br>1.22e-03	NA	
**CLCN1**<br>ENST00000343257.2<br>*het*	7:143039510 G>C<br>c.1842G>C<br>p.Lys614Asn<br>rs140205115	1.38e-03<br>2.16e-03	NA	
**COL6A1**<br>ENST00000361866.3<br>*het*	21:47410376 A>AG<br>c.1002+40_1002+41insG	2.72e-04<br>1.89e-03	NA	
**COL6A1**<br>ENST00000361866.3<br>*het*	21:47410379 A>ACGGGGAGGG[+]<br>c.1002+43_1002+44insCGGGGAGGGA[+]	0.00e+00<br>0.00e+00	NA	
**COL6A2**<br>ENST00000300527.4<br>*het*	21:47532521 C>T<br>c.714+30C>T	2.95e-04<br>4.92e-04	NA	
**DMD**<br><br>*het*	X:31132813 T>G	1.57e-04<br>4.16e-04	NA	
**DMPK**<br>ENST00000343373.4<br>*het*	19:46274987 ACCGGCCCGC[+]>A<br>c.1532+30_1532+41delGATGCGGGCC[+]	5.48e-04<br>9.41e-04	NA	
**DYSF**<br>ENST00000410020.3<br>*het*	2:71780887 A>C<br>c.1985-50A>C	7.18e-04<br>3.66e-03	NA	
**DYSF**<br>ENST00000410020.3	2:71780891 G>C<br>c.1985-46G>C	4.36e-04<br>1.73e-03	NA	

**Gene**<br>*Transcript*<br>*Genotype*[4]	**Gene Variant**[1]<br>*Transcript Change*[5]<br>*Protein Change*<br>*dbSNP*[7]	**ExAC AF**[2]<br>*Highest Pop*[6]	**ClinVar**[3]<br>*ClinVar Confidence*	**Notes**
het				
**DYSF**<br>ENST00000410020.3<br>*het*	2:71780895 T>C<br>c.1985-42T>C	0.00e+00<br>0.00e+00	NA	
**DYSF**<br>ENST00000410020.3<br>*het*	2:71780898 A>C<br>c.1985-39A>C	0.00e+00<br>0.00e+00	NA	
**DYSF**<br>ENST00000410020.3<br>*het*	2:71780900 T>C<br>c.1985-37T>C	0.00e+00<br>0.00e+00	NA	
**FLNC**<br>ENST00000325888.8<br>*het*	7:128482640 C>T<br>c.2277C>T<br>c.2277C>T(p.=)	0.00e+00<br>0.00e+00	NA	
**IGHMBP2**<br>ENST00000255078.3<br>*het*	11:68673601 C>G<br>c.151C>G<br>p.Gln51Glu<br>rs117061430	6.28e-03<br>1.44e-02	NA	
**ITGA7**<br>ENST00000257880.7<br>*het*	12:56080043 C>T<br>c.3371G>A<br>p.Arg1124Gln<br>rs201332795	4.01e-04<br>6.55e-04	NA	
**ITGA7**<br>ENST00000553804.1<br>*het*	12:56086993 C>T<br>c.2656G>A<br>p.Glu886Lys<br>rs144983062	4.01e-03<br>6.03e-03	NA	
**KBTBD13**<br>ENST00000432196.2<br>*het*	15:65369514 G>A<br>c.361G>A<br>p.Val121Met<br>rs201466173	7.09e-04<br>4.85e-03	NA	
**LAMB2**<br>ENST00000418109.1<br>*het*	3:49162352 C>T<br>c.2891G>A<br>p.Arg964Gln	2.48e-05<br>2.60e-04	NA	
**LDB3**	10:88439092 A>G	3.30e-05	NA	

**Gene**<br>*Transcript*<br>*Genotype*[4]	**Gene Variant**[1]<br>*Transcript Change*[5]<br>*Protein Change*<br>*dbSNP*[7]	**ExAC AF**[2]<br>*Highest Pop*[6]	**ClinVar**[3]<br>*ClinVar Confidence*	**Notes**
ENST00000429277.2<br>*het*	c.94-32A>G	2.90e-04		
**LDB3**<br>ENST00000429277.2<br>*het*	10:88451715 A>G<br>c.956A>G<br>p.Lys319Arg	6.65e-03<br>4.84e-02	NA	
**LDB3**<br>ENST00000429277.2<br>*het*	10:88478529 G>A<br>c.1918G>A<br>p.Val640Ile<br>rs45618633	6.99e-03<br>6.37e-02	NA	
**MYH3**<br>ENST00000583535.1<br>*het*	17:10555926 CTTT>C<br>c.205-49_205-47delAAA	0.00e+00<br>0.00e+00	NA	
**MYH3**<br>ENST00000583535.1<br>*het*	17:10555926 CTTTTT>C<br>c.205-51_205-47delAAAAA	0.00e+00<br>0.00e+00	NA	
**MYH3**<br>ENST00000583535.1<br>*het*	17:10555932 T>C<br>c.205-52A>G	0.00e+00<br>0.00e+00	NA	
**NEB**<br>ENST00000397345.3<br>*het*	2:152381168 CTTT>C<br>c.22273-41_22273-39delAAA	0.00e+00<br>0.00e+00	NA	
**NEB**<br>ENST00000397345.3<br>*het*	2:152381170 T>C<br>c.22273-40A>G	2.76e-05<br>2.37e-04	NA	
**NEB**<br>ENST00000397345.3<br>*het*	2:152381172 T>C<br>c.22273-42A>G	1.09e-05<br>9.79e-05	NA	
**NEB**<br>ENST00000397345.3<br>*het*	2:152456944 G>C<br>c.13788+12C>G	0.00e+00<br>0.00e+00	NA	

**Gene**<br>*Transcript*<br>*Genotype*[4]	**Gene Variant**[1]<br>*Transcript Change*[5]<br>*Protein Change*<br>*dbSNP*[7]	**ExAC AF**[2]<br>*Highest Pop*[6]	**ClinVar**[3]<br>*ClinVar Confidence*	**Notes**
**NEB**<br>ENST00000397345.3<br>*het*	2:152457023 T>C<br>c.13721A>G<br>p.His4574Arg	0.00e+00<br>0.00e+00	NA	
**PFKM**<br>ENST00000340802.6<br>*het*	12:48501129 ACT>A<br>c.-9-16_-9-15delCT	1.99e-03<br>2.73e-03	NA	
**PHKA1**<br>ENST00000373542.4<br>*het*	X:71813192 T>C<br>c.3073-68A>G	0.00e+00<br>0.00e+00	NA	
**POMGNT2**<br>ENST00000344697.2<br>*het*	3:43122465 G>A<br>c.459C>T<br>c.459C>T(p.=)	1.40e-04<br>4.32e-04	NA	
**PTPLA**<br>ENST00000361271.3<br>*het*	10:17646048 T>TA<br>c.258-3dupT	4.11e-03<br>1.88e-02	NA	
**RYR1**<br>ENST00000359596.3<br>*het*	19:38976331 G>A<br>c.5036G>A<br>p.Arg1679His<br>rs146504767	1.53e-03<br>2.63e-03	NA	
**SCN4A**<br>ENST00000435607.1<br>*het*	17:62038535 T>C<br>c.1845+18A>G	2.39e-03<br>4.07e-03	NA	
**SCN4A**<br>ENST00000435607.1<br>*het*	17:62049196 C>T<br>c.497G>A<br>p.Gly166Glu	0.00e+00<br>0.00e+00	NA	
**SCN4A**<br>ENST00000435607.1<br>*het*	17:62049198 T>G<br>c.495A>C<br>c.495A>C(p.=)	0.00e+00<br>0.00e+00	NA	
**STIM2**<br>ENST00000465503.1<br>*het*	4:27004500 T>A<br>c.804-49T>A	1.85e-03<br>1.07e-02	NA	

**Gene**<br>*Transcript*<br>*Genotype*[4]	**Gene Variant**[1]<br>*Transcript Change*[5]<br>*Protein Change*<br>*dbSNP*[7]	**ExAC AF**[2]<br>*Highest Pop*[6]	**ClinVar**[3]<br>*ClinVar Confidence*	**Notes**
**TNNT1**<br>ENST00000588981.1<br>*het*	19:55646893 C>CTT<br>c.612-1323_612-1322dupAA	0.00e+00<br>0.00e+00	NA	
**TTN**<br>ENST00000589042.1<br>*het*	2:179419925 G>GA<br>c.88307-47dupT	3.19e-03<br>1.32e-02	NA	
**TTN**<br>ENST00000589042.1<br>*het*	2:179517639 C>T<br>c.38895G>A<br>c.38895G>A(p.=)	8.55e-06<br>6.08e-05	NA	
**TTN**<br>ENST00000589042.1<br>*het*	2:179519303 T>C<br>c.38207-42A>G	3.33e-03<br>1.94e-02	NA	
**TTN**<br>ENST00000589042.1<br>*het*	2:179553730 AT>A<br>c.32095+49delA	6.92e-04<br>2.29e-03	NA	
**TTN**<br>ENST00000589042.1<br>*het*	2:179553730 ATT>A<br>c.32095+48_32095+49delAA	1.41e-03<br>3.44e-03	NA	
**TTN**<br>ENST00000589042.1<br>*het*	2:179634389 TATA>T<br>c.8902+14_8902+16delTAT	5.72e-04<br>1.62e-03	NA	
**UBA1**<br>ENST00000335972.6<br>*het*	X:47063147 T>C<br>c.1575+52T>C	0.00e+00<br>0.00e+00	NA	
**YARS2**<br>ENST00000324868.8<br>*het*	12:32905674 C>CAAA<br>c.947+1175_947+1177dupTTT	0.00e+00<br>0.00e+00	NA	
**YARS2**<br>ENST00000324868.8	12:32905674 CA>C<br>c.947+1177delT	0.00e+00<br>0.00e+00	NA	

**Gene**<br>*Transcript*<br>*Genotype*[4]	**Gene Variant**[1]<br>*Transcript Change*[5]<br>*Protein Change*<br>*dbSNP*[7]	**ExAC AF**[2]<br>*Highest Pop*[6]	**ClinVar**[3]<br>*ClinVar Confidence*	**Notes**
het				

Footnotes:
[1]Genomic variants are based on build37/hg19. This is a filtered list (AF $<$0.01) of variants containing **non-sense, frameshift, essential splice**,**missense** and **synonymous** variants.
[2]ExAC Allele Frequency (AF). For more information on ExAC please refer to *General Methodology* section.
[3]For more details on ClinVar and how confidence is rated please refer to *General Methodology* section.
[4]Genotype is either heterozygous (*het*), homozygous (*hom*) or hemizygous (*hem*) for the variant.
[5]Transcript changes are based on Ensembl.
[6]Highest allele frequency in any of the given ExAC populations (Pop).
[7]rsID if present in dbSNP version 141.
[+]Large insertion/deletions have been truncated.

Gene Coverage

Gene	Coverage[1]		Callable Sites[2] (%)		Uncallable Sites[2]	
	Cohort[3]	Sample[4]	Cohort	Sample	Cohort	Sample
ABHD5	83.68	91.38	100.00	94.44	0	67
ACADS	94.54	98.13	100.00	100.00	0	0
ACADVL	93.76	97.78	98.24	99.45	48	15
ACTA1	93.53	99.78	100.00	100.00	0	0
AGK	45.08	45.21	100.00	100.00	0	0
AGL	78.86	90.31	100.00	100.00	0	0
AGRN	81.32	94.95	99.10	96.55	63	241
ALG13	36.96	56.04	100.00	100.00	0	0
ALG14	54.11	61.88	100.00	100.00	0	0
ALG2	75.43	92.00	100.00	100.00	0	0
ANO5	77.61	67.02	100.00	100.00	0	0
ATP2A1	94.12	97.40	100.00	100.00	0	0
B3GALNT2	34.38	55.20	93.04	96.42	132	68
B3GNT1	58.75	90.00	99.22	100.00	10	0
BAG3	89.48	89.96	100.00	99.51	0	9
BIN1	84.84	91.92	100.00	100.00	0	0
C10orf2	97.85	100.00	100.00	100.00	0	0
CAPN3	91.79	96.82	100.00	100.00	0	0
CAV3	89.42	95.57	100.00	100.00	0	0
CCDC78	73.79	98.63	100.00	100.00	0	0
CFL2	77.81	96.75	100.00	100.00	0	0
CHAT	92.00	98.28	100.00	100.00	0	0
CHKB	89.99	90.83	100.00	100.00	0	0
CHRNA1	92.36	99.90	100.00	100.00	0	0
CHRNB1	93.26	99.79	100.00	100.00	0	0
CHRND	96.33	99.69	100.00	100.00	0	0
CHRNE	92.21	100.00	100.00	100.00	0	0
CHRNG	98.84	100.00	100.00	100.00	0	0
CLCN1	92.66	98.80	100.00	100.00	0	0
CNBP	71.89	99.30	100.00	100.00	0	0
CNTN1	74.15	67.12	100.00	100.00	0	0
COL12A1	46.85	45.11	100.00	99.97	0	3
COL6A1	90.04	98.94	100.00	100.00	0	0
COL6A2	90.63	98.66	100.00	100.00	0	0
COL6A3	90.25	99.07	99.59	99.59	43	43

Gene	Coverage[1]		Callable Sites[2] (%)		Uncallable Sites[2]	
	Cohort[3]	Sample[4]	Cohort	Sample	Cohort	Sample
COLQ	92.21	97.68	100.00	100.00	0	0
COX15	96.39	99.77	100.00	100.00	0	0
CPT2	90.21	99.51	100.00	100.00	0	0
CRYAB	90.19	99.07	100.00	100.00	0	0
DAG1	96.25	99.99	100.00	100.00	0	0
DES	84.69	96.23	100.00	100.00	0	0
DMD	62.89	72.07	100.00	100.00	0	0
DNAJB6	43.18	69.43	100.00	100.00	0	0
DNM2	86.20	89.63	98.66	97.32	50	100
DOK7	73.98	87.13	100.00	100.00	0	0
DOLK	95.96	100.00	100.00	100.00	0	0
DPAGT1	94.40	99.63	100.00	100.00	0	0
DPM1	67.41	89.93	95.94	100.00	44	0
DPM2	31.60	57.71	100.00	100.00	0	0
DPM3	88.01	100.00	100.00	100.00	0	0
DYSF	92.98	96.42	100.00	100.00	0	0
EMD	74.97	99.83	100.00	100.00	0	0
ENO3	98.64	100.00	100.00	100.00	0	0
ETFA	55.54	74.07	87.45	89.12	232	201
ETFB	98.36	99.89	100.00	100.00	0	0
ETFDH	63.28	85.94	100.00	100.00	0	0
FAM111B	63.64	83.81	100.00	100.00	0	0
FHL1	72.44	91.36	100.00	100.00	0	0
FKBP14	28.20	48.16	100.00	100.00	0	0
FKRP	57.88	74.97	100.00	100.00	0	0
FKTN	67.28	75.96	100.00	100.00	0	0
FLNC	96.59	99.99	100.00	100.00	0	0
GAA	86.99	96.29	100.00	100.00	0	0
GBE1	26.86	24.02	100.00	99.39	0	15
GFPT1	76.75	96.22	100.00	100.00	0	0
GMPPB	74.62	96.58	100.00	100.00	0	0
GNE	86.65	96.26	100.00	100.00	0	0
GYG1	83.01	98.16	100.00	100.00	0	0
GYS1	90.90	98.75	100.00	100.00	0	0
HNRNPDL	46.08	76.51	91.43	100.00	120	0
IGHMBP2	93.09	99.35	100.00	100.00	0	0

Gene	Coverage[1]		Callable Sites[2] (%)		Uncallable Sites[2]	
	Cohort[3]	Sample[4]	Cohort	Sample	Cohort	Sample
ISCU	72.53	83.38	100.00	100.00	0	0
ISPD	34.20	33.65	82.16	82.16	277	277
ITGA7	91.25	97.19	100.00	100.00	0	0
KBTBD13	52.77	87.36	100.00	100.00	0	0
KCNJ2	99.39	100.00	100.00	100.00	0	0
KLHL40	77.98	95.74	100.00	100.00	0	0
KLHL41	60.50	79.21	100.00	100.00	0	0
KLHL9	86.83	98.36	100.00	100.00	0	0
LAMA2	86.67	80.82	100.00	100.00	0	0
LAMB2	98.07	100.00	100.00	100.00	0	0
LAMP2	53.21	85.48	100.00	100.00	0	0
LARGE	90.79	96.74	100.00	100.00	0	0
LDB3	91.97	99.12	100.00	100.00	0	0
LDHA	74.39	92.98	100.00	100.00	0	0
LIMS2	57.87	75.20	90.90	90.90	134	134
LMNA	89.36	96.77	100.00	100.00	0	0
LPIN1	79.87	94.80	100.00	100.00	0	0
LRP4	96.39	98.94	100.00	99.44	0	36
MATR3	74.40	94.80	95.54	98.34	140	52
MEGF10	47.32	47.64	100.00	100.00	0	0
MSTN	76.55	72.92	100.00	100.00	0	0
MTM1	58.61	83.02	100.00	100.00	0	0
MTMR14	85.73	93.50	100.00	100.00	0	0
MTTP	81.84	78.70	100.00	100.00	0	0
MUSK	53.15	51.15	100.00	100.00	0	0
MYBPC3	53.17	77.93	100.00	100.00	0	0
MYF6	93.63	96.92	100.00	100.00	0	0
MYH14	67.45	83.07	100.00	100.00	0	0
MYH2	93.52	99.41	100.00	100.00	0	0
MYH3	97.23	99.83	100.00	99.98	0	1
MYH7	98.21	100.00	100.00	100.00	0	0
MYOT	79.74	94.22	100.00	100.00	0	0
NEB	47.84	52.33	90.81	93.91	2694	1784
OPA1	66.67	82.41	100.00	100.00	0	0
ORAI1	80.83	82.17	89.78	94.42	97	53
PABPN1	68.47	75.87	91.92	91.83	86	87

Gene	Coverage[1]		Callable Sites[2] (%)		Uncallable Sites[2]	
	Cohort[3]	Sample[4]	Cohort	Sample	Cohort	Sample
PFKM	85.99	89.45	100.00	100.00	0	0
PGAM2	99.43	100.00	100.00	100.00	0	0
PGK1	81.41	99.77	100.00	100.00	0	0
PGM1	92.24	94.67	100.00	100.00	0	0
PHKA1	67.97	88.56	100.00	100.00	0	0
PLEC	88.29	98.37	99.77	99.77	36	36
PNPLA2	80.60	89.14	99.41	99.82	10	3
POLG	93.11	98.98	100.00	100.00	0	0
POLG2	71.99	90.31	100.00	100.00	0	0
POMGNT1	92.89	98.82	100.00	100.00	0	0
POMGNT2	88.11	99.70	100.00	100.00	0	0
POMK	87.21	99.75	100.00	100.00	0	0
POMT1	94.51	99.17	100.00	100.00	0	0
POMT2	91.63	98.73	100.00	100.00	0	0
PREPL	49.65	58.35	100.00	99.96	0	1
PRKAG2	77.18	89.07	100.00	96.17	0	90
PTPLA	24.92	45.61	99.52	98.81	6	15
PTRF	88.09	97.57	100.00	100.00	0	0
PYGM	98.99	99.92	100.00	100.00	0	0
RAPSN	85.59	96.28	100.00	100.00	0	0
RRM2B	71.90	83.82	91.25	91.25	141	141
RYR1	92.09	94.93	98.00	98.74	350	221
SCN4A	76.72	90.73	100.00	100.00	0	0
SEPN1	80.21	83.85	90.00	89.95	203	204
SGCA	90.26	95.06	100.00	100.00	0	0
SGCB	69.82	86.46	95.44	95.44	53	53
SGCD	35.63	28.20	100.00	100.00	0	0
SGCG	87.47	99.35	100.00	100.00	0	0
SIL1	95.68	98.55	100.00	100.00	0	0
SLC22A5	93.44	99.46	100.00	100.00	0	0
SLC25A20	94.03	96.85	100.00	100.00	0	0
SLC25A4	78.74	87.86	100.00	96.76	0	33
SLC52A3	95.19	99.84	100.00	100.00	0	0
SMCHD1	28.12	41.38	98.09	96.53	135	245
STAC3	65.39	87.12	100.00	100.00	0	0
STIM1	88.16	96.49	100.00	100.00	0	0

Gene	Coverage[1]		Callable Sites[2] (%)		Uncallable Sites[2]	
	Cohort[3]	Sample[4]	Cohort	Sample	Cohort	Sample
STIM2	44.95	44.59	98.13	92.65	53	208
SUCLA2	74.34	96.62	100.00	100.00	0	0
SYNE1	86.57	94.08	100.00	100.00	0	0
SYNE2	79.41	95.81	100.00	100.00	0	0
TARDBP	71.27	86.81	89.56	89.56	165	165
TAZ	84.39	99.66	100.00	100.00	0	0
TCAP	88.42	100.00	100.00	100.00	0	0
TIA1	34.83	62.37	100.00	100.00	0	0
TK2	77.78	85.36	95.71	97.63	58	32
TMEM43	76.20	89.51	100.00	100.00	0	0
TMEM5	35.25	51.94	100.00	100.00	0	0
TNNI2	95.21	100.00	100.00	100.00	0	0
TNNT1	71.84	88.68	100.00	100.00	0	0
TNNT3	99.13	100.00	100.00	100.00	0	0
TNPO3	46.54	62.67	100.00	100.00	0	0
TOR1AIP1	55.41	71.89	100.00	100.00	0	0
TPM2	92.36	99.92	100.00	100.00	0	0
TPM3	96.92	99.27	100.00	100.00	0	0
TRAPPC11	41.68	65.85	100.00	100.00	0	0
TRIM32	96.74	99.94	100.00	100.00	0	0
TTN	64.78	64.72	99.67	99.59	402	497
UBA1	87.97	98.47	100.00	100.00	0	0
VAPB	82.07	91.70	100.00	100.00	0	0
VCP	98.44	98.90	100.00	100.00	0	0
VMA21	43.73	68.90	100.00	100.00	0	0
YARS2	88.00	99.99	100.00	100.00	0	0

Notes:

[1]Average per-base coverage for gene. Genes that are not completely callable are highlighted in **pink**. For more information on how coverage was calculated, please refer to *General Methodology* section.

[2]The percent of sites that are considered callable for this gene. For more information on how callability is determined, please refer to *General Methodology* section.

[3]The average for samples included in the cohort.

[4]The metric for the sample in this report.

General Methodology

Exome Sequencing
We performed whole exome sequencing on DNA samples at the Broad Institute's Genomics Platform, using Illumina exome capture, 38 Mb baited target, and the Broad's in-solution hybrid selection process. For input DNA we used >250 ng of DNA, at >2 $ng/\mu l$. Our exome-sequencing pipeline included sample plating, library preparation (2-plexing of samples per hybridization), hybrid capture, sequencing (76 bp paired reads), sample identification QC check, and data storage. Our hybrid selection libraries cover >80% of targets at 20x and a mean target coverage of >80x. The exome sequencing data was de-multiplexed and each sample's sequence data were aggregated into a single Picard BAM file.

Variant Calling, Filtering and Annotation
Exome sequencing data was processed through a pipeline based on *Picard*, using base quality score recalibration and local realignment at known indels. We used the *BWA* aligner for mapping reads to the human genome build 37 (hg19). Single Nucleotide Polymorphism (SNPs) and insertions/deletions (indels) were jointly called across all samples using *Genome Analysis Toolkit (GATK)* HaplotypeCaller package version 3.1. Default filters were applied to SNP and indel calls using the GATK Variant Quality Score Recalibration (VQSR) approach. Lastly, the variants were annotated using *Variant Effect Predictor (VEP)*.

Ancestry Inference
A set of ~5,800 common SNPs was used to perform principal component analysis (PCA) with 1000 genomes samples and ancestry was inferred based on distance to nearest cluster formed by Exome Aggregation Consortium (ExAC) samples. This analysis is able to identify individuals with a European, South Asian, East Asian, African or Latino ancestry.

Gender Inference
The gender of the individual was inferred using the fraction of heterozygous calls amongst a set of common variant on chromosome X. In addition, the Y chromosome coverage normalized to chromosome 20 coverage was also used to infer gender.

Gene coverage
The coding bases (including 10 bp flanking region) of the candidate genes was determined using the Gencode v19 regions. The per base coverage of each sample was determined using *samtools depth*. Only reads with mapping quality (MQ) ≥20 and bases with base quality (BQ) ≥10 were included in the coverage metric. Lastly, bases with from overlapping reads were only counted once. A site with coverage >6 was considered callable.

Analysis
The variant call set was uploaded on to *xBrowse* and an analysis limited to the candidate gene list was performed using the various inheritance patterns. The main report contains variants restricted to nonsense, frameshift, essential splice site and missense variants and filtered on variant site and genotype quality. The appendix listing each gene contains all variants discovered regardless of annotation and quality.

Exome Aggregation Consortium (ExAC) data set
The Exome Aggregation Consortium (ExAC) is a coalition of investigators seeking to aggregate and harmonize exome sequencing data from a wide variety of large-scale sequencing projects, and to make summary data available for the wider scientific community. The data set contains >60,000 unrelated individuals sequenced as part of various disease-specific and population genetic studies. The allele frequencies are summarized into the major ancestries European, Non-Finnish European, South Asian, East Asian, African and Latino. For more details please visit http://exac.broadinstitute.org/about

ClinVar Database

ClinVar is a freely accessible, public archive of reports of the relationships among human variations and phenotypes, with supporting evidence. The level of confidence in the accuracy of variation calls and assertions of clinical significance depends in large part on the supporting evidence and is variable for the variants included in ClinVar. A review status (stars) is also assigned by ClinVar to each variant, to indicate the level of supporting evidence.

Stars	Review Status
None	Either conficting interpretations (in which case the independent values are enumerated), or no submitter provided an interpretation
1	One submitter provided an interpretation (classified by single submitter)
2	Two or more submitters provided the same interpretation (classified by multiple submitters)
3	Reviewed by expert panel
4	Practice guideline (reviewed by professional society)

Tools/Resources

Tool/Resource	Link
BWA	https://github.com/lh3/bwa
Picard	https://github.com/broadinstitute/picard
Genome Analysis Toolkit (GATK)	https://www.broadinstitute.org/gatk
samtools	https://github.com/samtools/samtools
Gencode v19	http://www.gencodegenes.org/releases/19.html
Variant Effect Predictor (VEP)	http://www.ensembl.org/info/docs/tools/vep/index.html
xBrowse	https://xbrowse.broadinstitute.org
Exome Aggregation Consortium (ExAC)	http://exac.broadinstitute.org
ClinVar	http://www.ncbi.nlm.nih.gov/clinvar

Jarmula, Lusakowska et al.

Supplementary Material 4 .
Tables.

Suppl. Table 1. Percentage hydrogen bonds occupancies of the residues of Asp81 and Gly81 in the trajectories of wild-type and Asp81Gly mutant structures, respectively, of ANO5.

	Hydrogen bond occupancy [%] with:			
	Arg78	Tyr652	Gln79	Gln256
Asp81	100.0 (A)*	15.8 (A)	0.66 (A)	0.0
Gly81	0.0	0.0	27.2 (D)	24.9 (A)

* (D) and (A) stand for the respective residue at position 81 acting as hydrogen bond donor or acceptor, respectively.

Suppl. Table 2. Percentage hydrogen bonds occupancies of the residues of Lys132 and Met132 in the trajectories of wild-type and Lys132Met mutant structures, respectively, of ANO5.

	Hydrogen bond occupancy [%] with:
	Leu115
Lys132	55.4 (D)*
Met132	69.8 (D) + 71.3 (A)

* (D) and (A) stand for the respective residue at position 132 acting as hydrogen bond donor or acceptor, respectively.

Suppl. Table 3. Percentage hydrophobic interactions occupancies of the residues of Lys132 and Met132 in the trajectories of wild-type and Lys132Met mutant structures, respectively, of ANO5.

	Hydrophobic interaction occupancy [%] with:	
	Leu115	Ala135
Lys132	24.2	0.0
Met132	85.5	97.4

Suppl. Table 4. Percentage hydrogen bonds occupancies of the residues of Ser555 and Ile555 in the trajectories of wild-type and Ser555Ile mutant structures, respectively, of ANO5.

	Hydrogen bond occupancy [%] with:		
	Glu544	Glu551	Lys559
Ser555	97.8 (D)*	19.1 (D)	15.3 (A)
Ile555	0.0	68.9 (D)	52.3 (A)

* (D) and (A) stand for the respective residue at position 555 acting as hydrogen bond donor or acceptor, respectively.

Suppl. Table 5. Percentage hydrophobic interactions occupancies of the residues of Ser555 and Ile555 in the trajectories of wild-type and Ser555Ile mutant structures, respectively, of ANO5.

	Hydrophobic interaction occupancy [%] with:
	Pro546
Ser555	0.0
Ile555	24.2

Suppl. Table 6. Percentage hydrogen bonds occupancies of the residues of Tyr671 and Cys671 in the trajectories of wild-type and Tyr671Cys mutant structures, respectively, of ANO5.

	Hydrogen bond occupancy [%] with:		
	Arg234	Gly668	Glu675
Tyr671	89.2 (D)*	33.6 (D)	34.6 (A)
Cys671	0.0	32.8 (D)	0.0

* (D) and (A) stand for the respective residue at position 671 acting as hydrogen bond donor or acceptor, respectively.

Suppl. Table 7. Percentage hydrophobic interactions occupancies of the residues of Tyr671 and Cys671 in the trajectories of wild-type and Tyr671Cys mutant structures, respectively, of ANO5.

	Hydrophobic interaction occupancy [%] with:	
	Lys233	Met560
Tyr671	20.6	91.2
Cys671	3.0	0.0

Suppl. Table 8. Percentage hydrogen bonds occupancies of the residues of Arg758 and Cys758 in the trajectories of wild-type and Arg758Cys mutant structures, respectively, of ANO5.

	Hydrogen bond occupancy [%] with:						
	Glu816	Asn817	Lys818	His821	Asn822	Met823	Gln824
Arg758	65.8 (D)*	8.9 (D)	5.3 (D)	49.7 (D)	55.6 (A)	31.6 (A)	0.0
Cys758	0.1 (D)	19.9 (D)	0.0	0.0	0.0	0.0	78.1 (A)

* (D) and (A) stand for the respective residue at position 758 acting as hydrogen bond donor or acceptor, respectively.

Suppl. Table 9. Percentage hydrophobic interactions occupancies of the residues of Arg758 and Cys758 in the trajectories of wild-type and Arg758Cys mutant structures, respectively, of ANO5.

	Hydrophobic interaction occupancy [%] with:			
	Ile756	Val760	Tyr761	Met823
Arg758	99.1	0.0	0.0	84.2
Cys758	0.0	60.1	71.9	0.0

Suppl. Table 10. Percentage hydrogen bonds occupancies of the residues of HIS841 and Asp841 in the trajectories of wild-type and His841Asp mutant structures, respectively, of ANO5.

	Hydrogen bond occupancy [%] with:				
	Arg364	Asn366	Ile837	Val838	Leu845
His841	20.2 (A)*	-	18.8 (D)	3.9 (D)	29.1 (A)
Asp841	60.7 (A)	31.5 (A)	1.8 (D)	7.6 (D)	16.0 (A)

* (D) and (A) stand for the respective residue at position 841 acting as hydrogen bond donor or acceptor, respectively.

Suppl. Table 11. Percentage hydrophobic interactions occupancies of the residues of His841 and Asp841 in the trajectories of wild-type and His841Asp mutant structures, respectively, of ANO5.

	Hydrophobic interaction occupancy [%] with:		
	Arg364	Phe844	Leu845
His841	57.2	28.1	87.6
Asp841	-	-	-

Suppl. Table 12. Hydrophobic contacts between A1, A2, B1 and B2 α-helices present in the average structures from the trajectories of the wild-type ANO5 and its mutants.

System	Residue 1	α-helix	Residue 2	α-helix	Contact type
**1. ANO5**					
	Phe 624	A1	Trp 1551	B1	π-π stacked
	Ala 630, Ile 631	A1	Trp 710	A2	amide-π stacked
	Met 618	A1	Leu 696	A2	alkyl
	Ala 630	A1	Val 707	A2	alkyl
	Ala 635	A1	Ile 1540	B1	alkyl
	Ala 635	A1	Val 1616	B2	alkyl
	Ala 635	A1	Val 1620	B2	alkyl
	Leu 636	A1	Ile 1540	B1	alkyl
	Met 618	A1	Ala 697	A2	alkyl
	Met 1531	B1	Leu 1609	B2	alkyl
	Met 1531	B1	Ala 1606	B2	alkyl
	Leu 634	A1	Val 1616	B2	alkyl
	Ile 631	A1	Val 1620	B2	alkyl
	Tyr 632	A1	Ile 1540	B1	π-alkyl
	Tyr 632	A1	Ile 1544	B1	π-alkyl
	Pro 633	A1	Trp 710	A2	π-alkyl
	Leu 634	A1	Trp 710	A2	π-alkyl
	Lys 628	A1	Trp 1551	B1	π-alkyl
	Ile 631	A1	Trp 1623	B2	π-alkyl
	Lys 628	A1	Trp 1623	B2	π-alkyl
**2. Asp81Gly**					
	Met 618	A1	Leu 696	A2	alkyl
	Ala 635	A1	Ile 1544	B1	alkyl
	Met 618	A1	Ala 697	A2	alkyl
	Trp 638	A1	Ile 1536	B1	π-alkyl
	Ile 627	A1	Trp 1551	B1	π-alkyl
**3. Lys132Met**					
	Trp 639	A1	Phe 1537	B1	π-π T-shaped
	Met 618	A1	Leu 696	A2	alkyl
	Ile 631	A1	Ile 1540	B1	alkyl

	Ala 635	A1	Ile 1536	B1	alkyl
	Met 618	A1	Ala 693	A2	alkyl
	Met 618	A1	Ala 697	A2	alkyl
	Ile 623	A1	Val 703	A2	alkyl
	Met 1531	B1	Leu 1609	B2	alkyl
	Ile 627	A1	Lys 1541	B1	alkyl
	Ala 1543	B1	Val 1620	B2	alkyl
	Ala 1543	B1	Lys 1624	B2	alkyl
	Ile 623	A1	Ala 1548	B1	alkyl
	Met 1531	B1	Ala 1606	B2	alkyl
	Met 1531	B1	Ala 1610	B2	alkyl
	Trp 638	A1	Ile 1536	B1	π-alkyl
	Ala 630	A1	Trp 710	A2	π-alkyl
	Ile 631	A1	Trp710	A2	π-alkyl
**4. Ser555Ile**					
	Trp 639	A1	Ile 1536	B1	π-sigma
	Phe 624	A1	Trp 1552	B1	π-π T-shaped
	Met 618	A1	Leu 696	A2	alkyl
	Leu 634	A1	Ile 1540	B1	alkyl
	Ala 635	A1	Ile 1536	B1	alkyl
	Met 618	A1	Ala 693	A2	alkyl
	Met 618	A1	Ala 697	A2	alkyl
	Met 1531	B1	Leu 1609	B2	alkyl
	Ala 1543	B1	Val 1620	B2	alkyl
	Ile 627	A1	Ala 1548	B1	alkyl
	Leu 1547	B1	Lys 1624	B2	alkyl
	Trp 638	A1	Ile 1540	B1	π-alkyl
	Trp 639	A1	Ile 1536	B1	π-alkyl
	Ile 623	A1	Trp 1552	B1	π-alkyl
	Ile 627	A1	Trp 1552	B1	π-alkyl
**5. Tyr671Cys**					
	Met 618	A1	Leu 696	A2	alkyl
	Ala 630	A1	Val 707	A2	alkyl
	Ile 631	A1	Ile 1540	B1	alkyl
	Ile 631	A1	Ile 1544	B1	alkyl

	Leu 634	A1	Ile 1536	B1	alkyl
	Leu 634	A1	Ile 1540	B1	alkyl
	Ala 635	A1	Ile 1540	B1	alkyl
	Met 618	A1	Ala 697	A2	alkyl
	Ala 1543	B1	Lys 1624	B2	alkyl
	Ile 631	A1	Ala 1548	B1	alkyl
	Leu 1527	B1	Ala 1606	B2	alkyl
	Ala 630	A1	Trp 710	A2	π-alkyl
	Leu 634	A1	Trp 710	A2	π-alkyl
	Ile 699	A2	Trp1551	B1	π-alkyl
**6. Arg758Cys**					
	Ile 623	A1	Leu 1547	B1	alkyl
	Ala 635	A1	Ile 1536	B1	alkyl
	Met 1531	B1	Leu 1609	B2	alkyl
	Ile 623	A1	Ala 1548	B1	alkyl
**7. His841Asp**					
	Trp 639	A1	Phe 1537	B1	π-π stacked
	Trp 638, Trp 639	A1	Phe 1537	B1	amide-π stacked
	Met 618	A1	Leu 696	A2	alkyl
	Ile 627	A1	Leu 1547	B1	alkyl
	Lys 628	A1	Ile 1544	B1	alkyl
	Ala 635	A1	Ile 1540	B1	alkyl
	Met 618	A1	Ala 693	A2	alkyl
	Met 1531	B1	Leu 1609	B2	alkyl
	Met 1531	B1	Ala 1610	B2	alkyl
	Phe 624	A1	Ile 1544	B1	π-alkyl
	Trp 638	A1	Ile 1536	B1	π-alkyl
	Ile 616	A1	Trp 1551	B1	π-alkyl
	Ile 623	A1	Trp 1551	B1	π-alkyl
	Leu 1547	B1	Trp 1623	B2	π-alkyl

Suppl. Table 13. Hydrogen bonds above 10% between central α-helices from chains A and B present in the trajectories of the wild-type ANO5 and its mutants.

System	Acceptor	α-helix	Donor	α-helix	% in trajectory
ANO5	Ile 1540	B1	Tyr 632	A1	71.9
ANO5	Ile 627	A1	Trp 1623	B2	57.4
Asp81Gly	Asn 1550	B1	Asn 626	A1	45.2
His841Asp	Thr 1532	B1	Trp 638	A1	31.7
Lys132Met	Thr 1532	B1	Trp 638	A1	27.5

Jarmula, Lusakowska et al.

Supplementary Material 5. Secondary structure assignment.

The graphs shown below present evolution of secondary structure assignment of the A1/A2/B1/B2 and 669-684 α-helices in the course of trajectories of wild-type ANO5 and its mutants: Asp81Gly, Lys132Met, Ser555Ile, Tyr671Cys, Arg758Cys and His841Asp. The assignment for α-helix 669-684 is provided only for the wild-type protein and Tyr671Cys mutant as Tyr671 residue is located within this helix.

ANO5

Helix A1

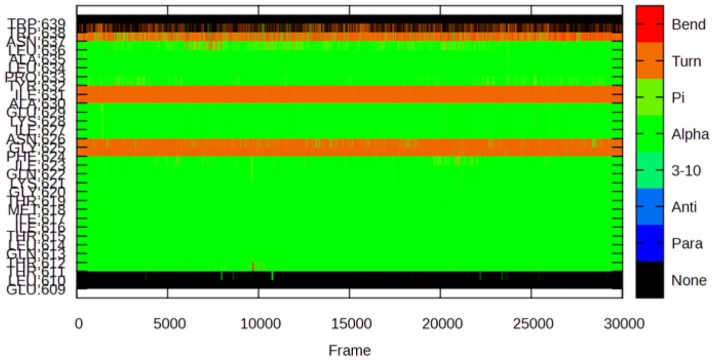

Helix A2

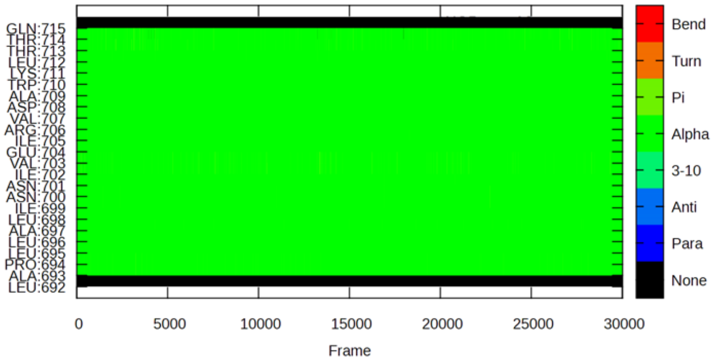

Helix B1

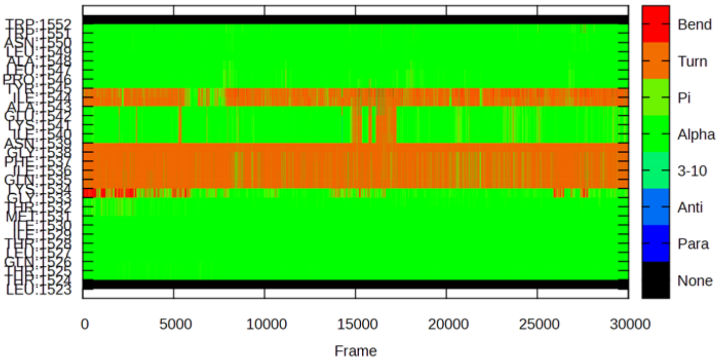

Helix B2

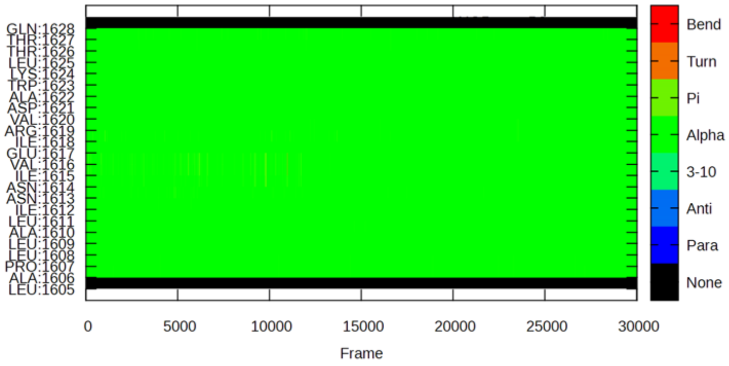

Helix 669-684

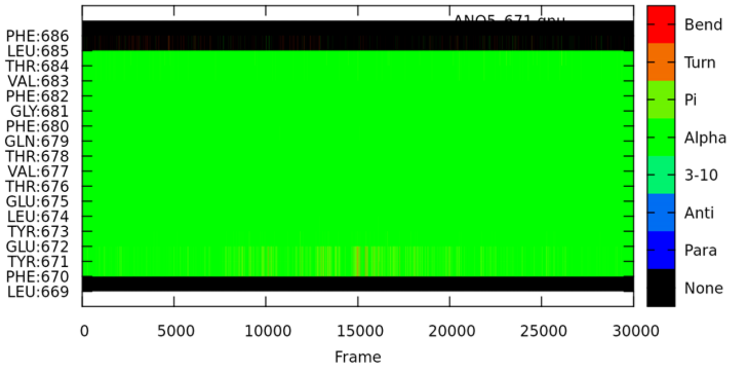

Asp81Gly

Helix A1

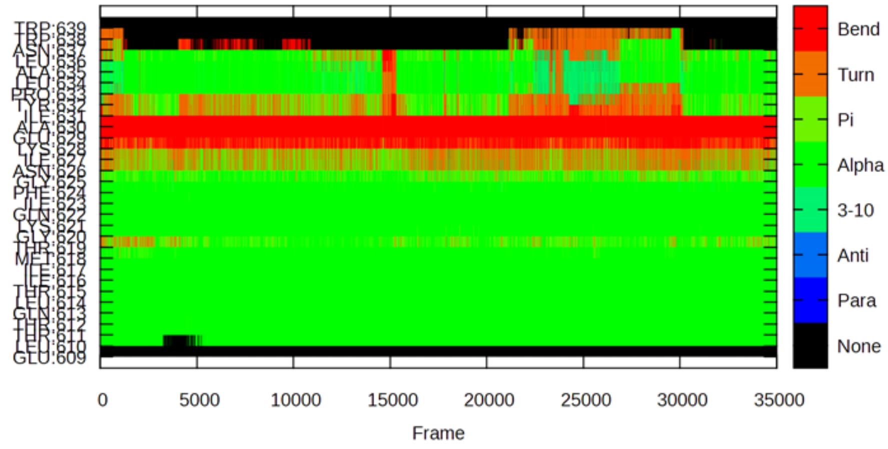

Helix A2

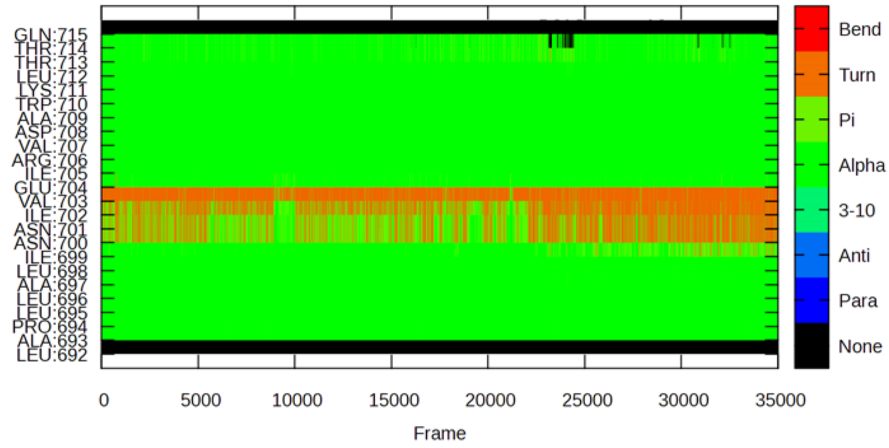

Helix B1

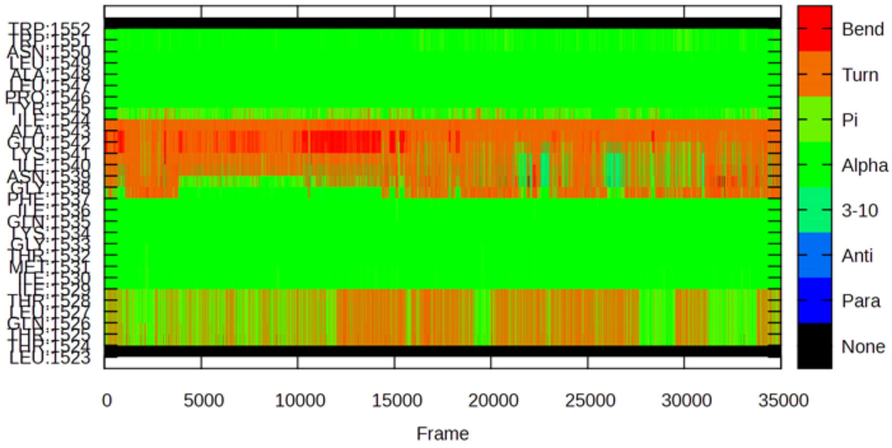

Helix B2

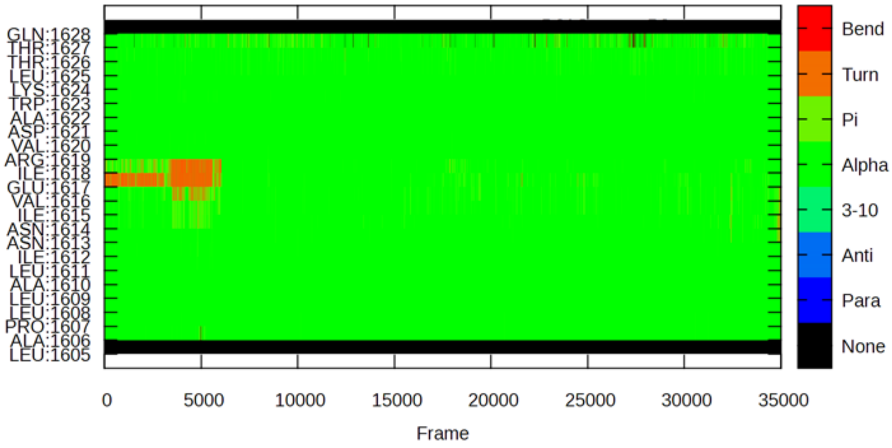

Lys132Met

Helix A1

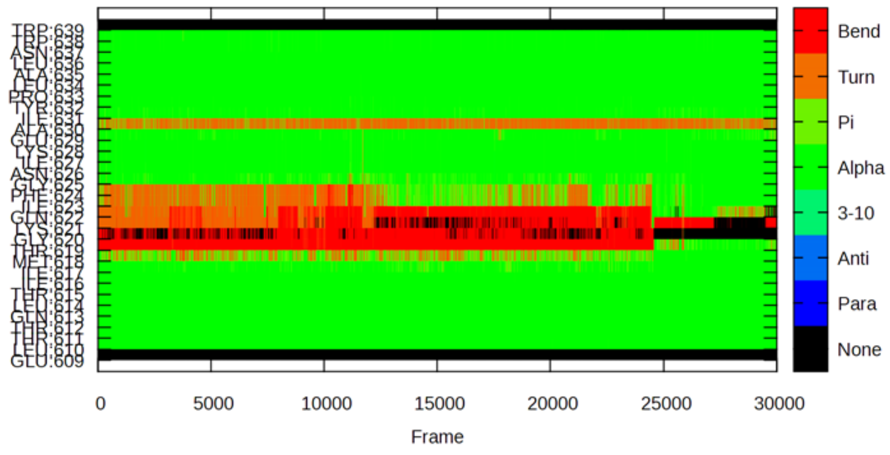

Helix A2

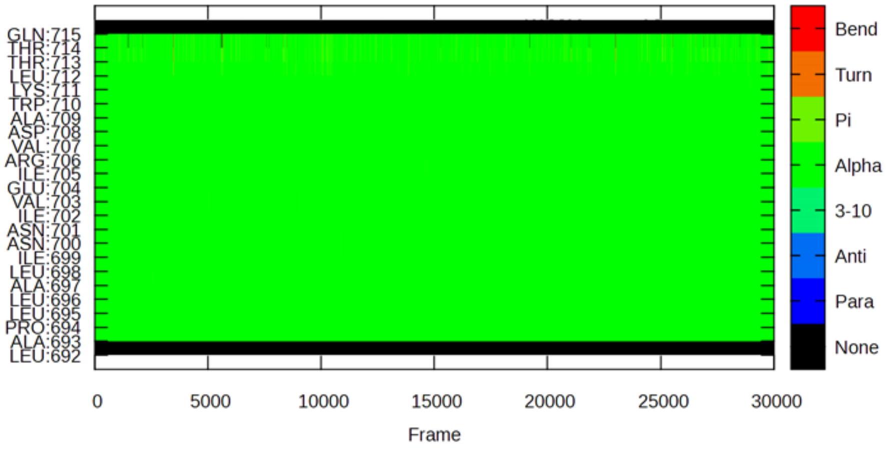

Helix B1

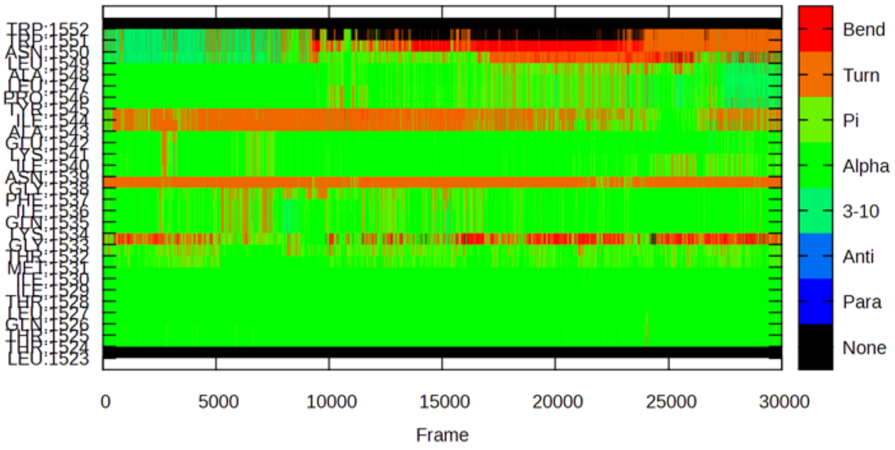

Helix B2

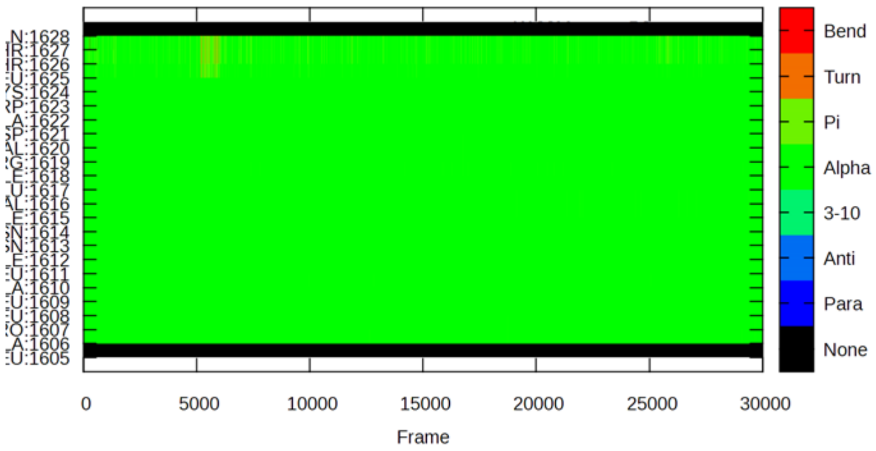

Ser555Ile

Helix A1

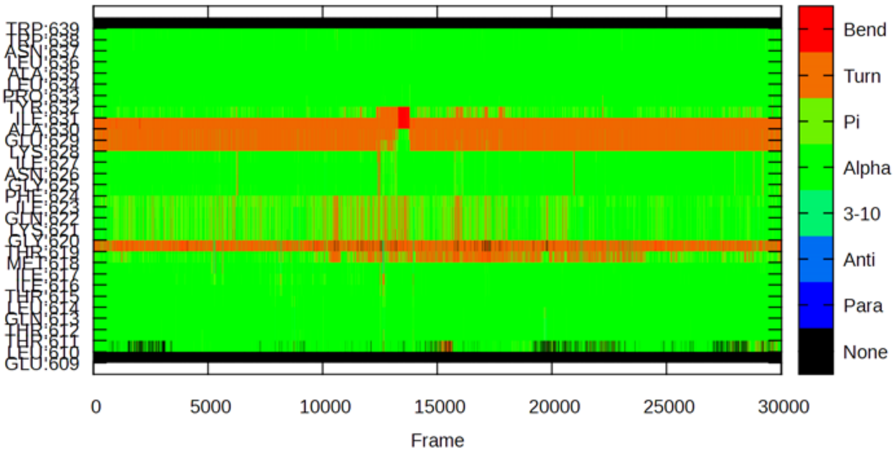

Helix A2

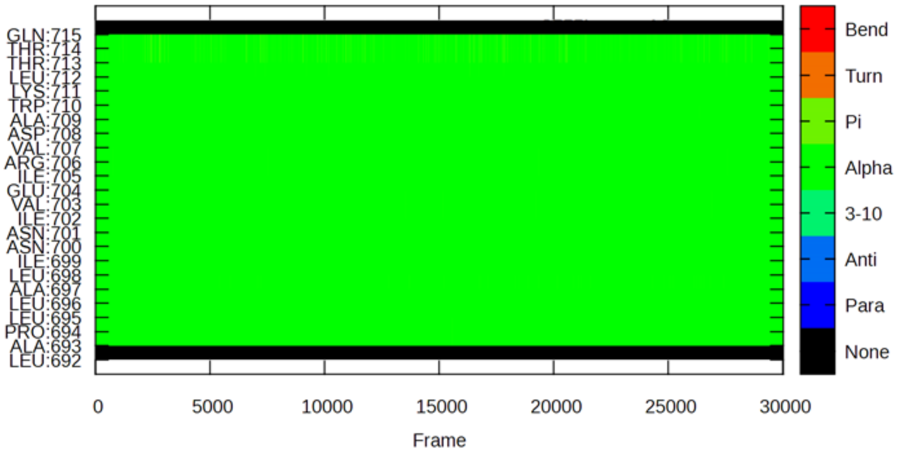

Helix B1

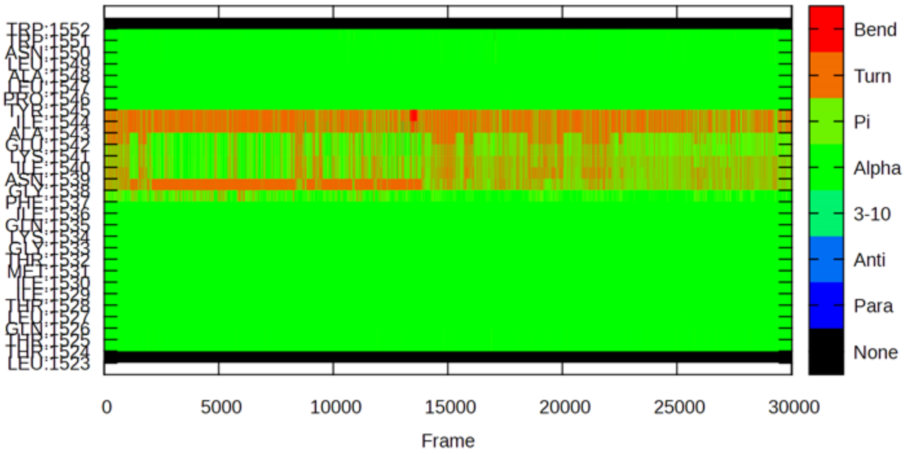

Helix B2

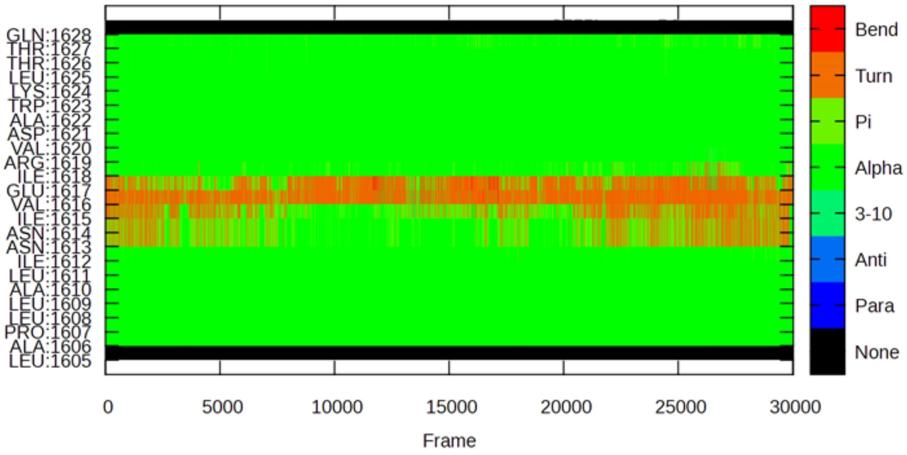

Tyr671Cys

Helix A1

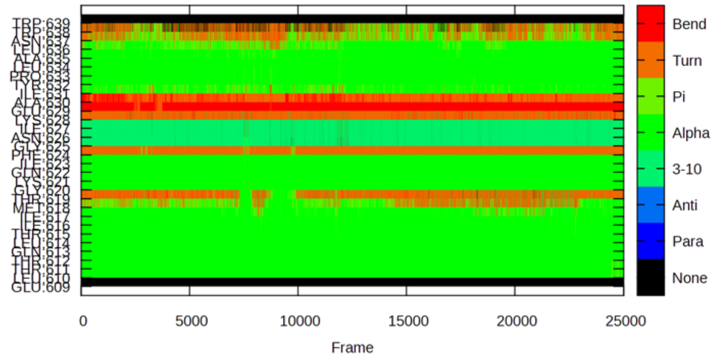

Helix A2

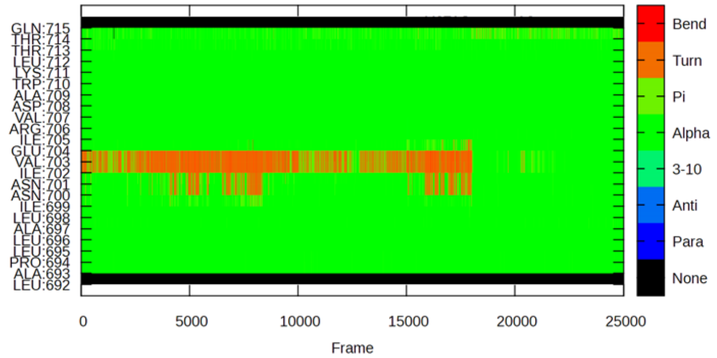

Helix B1

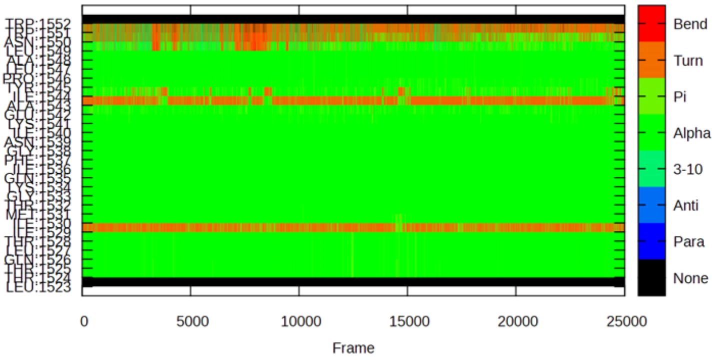

Helix B2

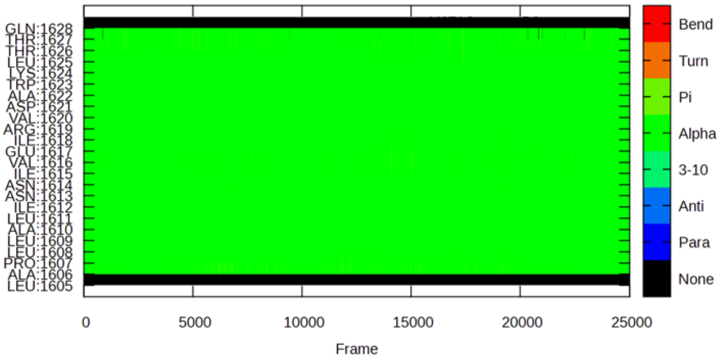

Helix 669-684

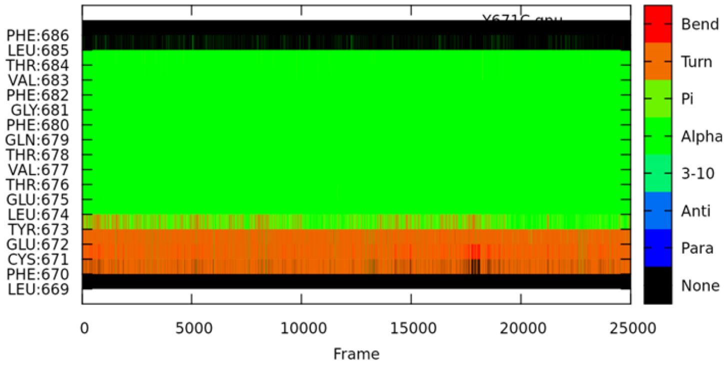

Arg758Cys

Helix A1

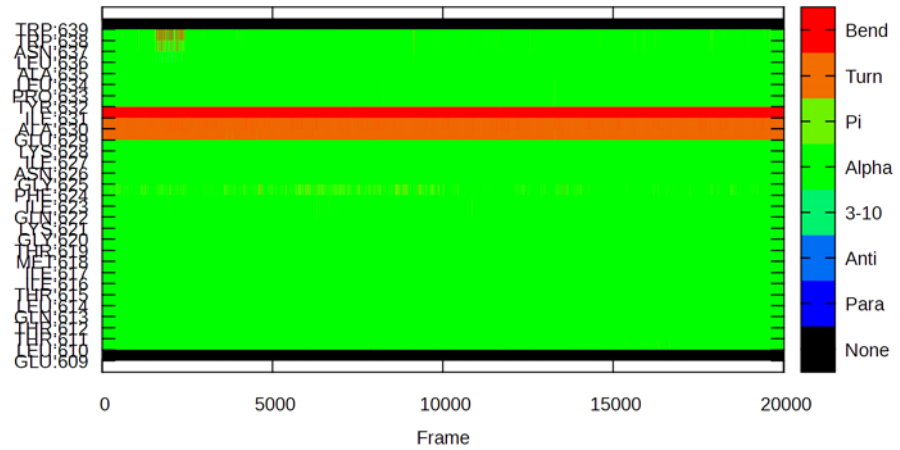

Helix A2

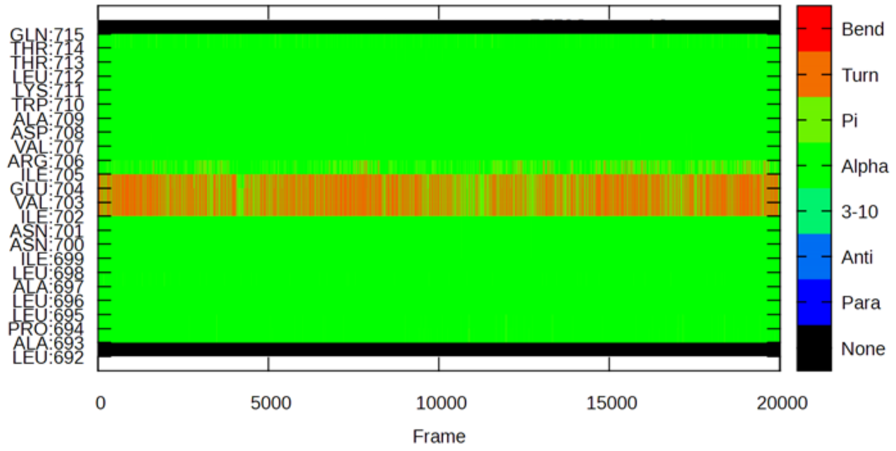

Helix B1

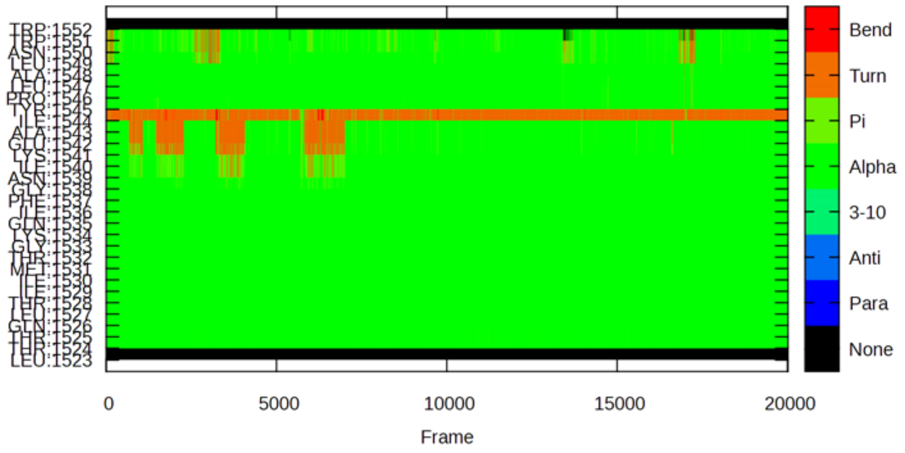

Helix B2

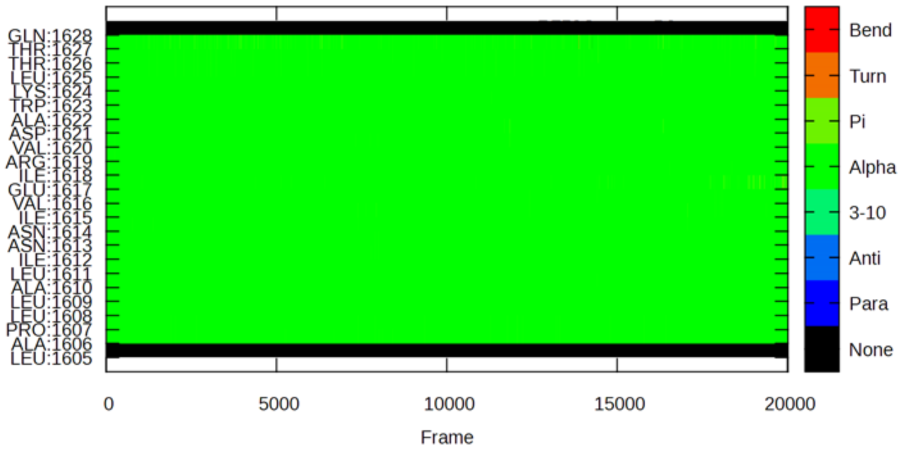

His841Asp

Helix A1

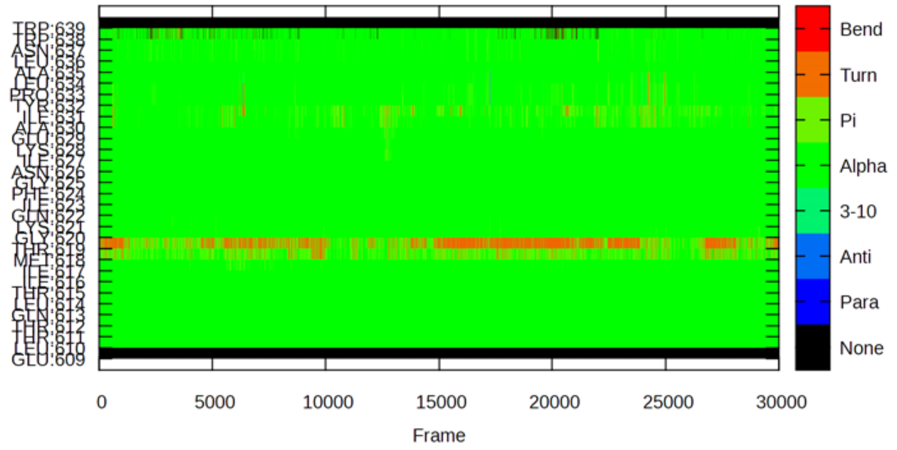

Helix A2

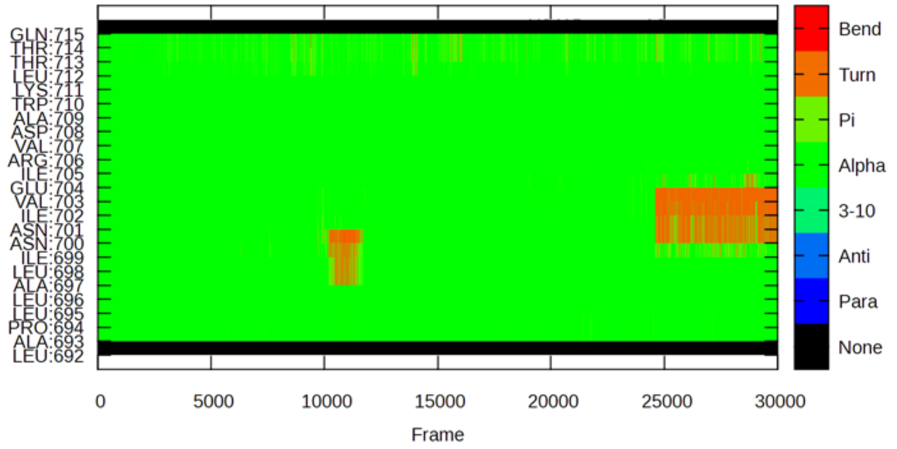

Helix B1

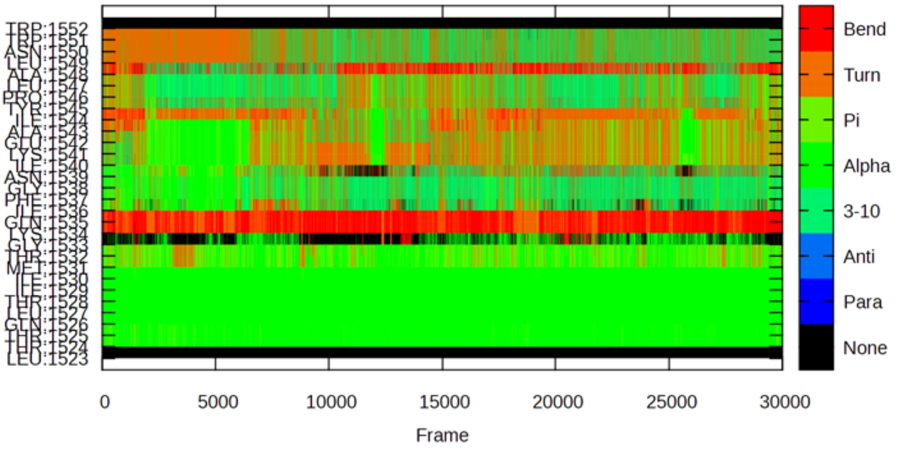

Helix B2

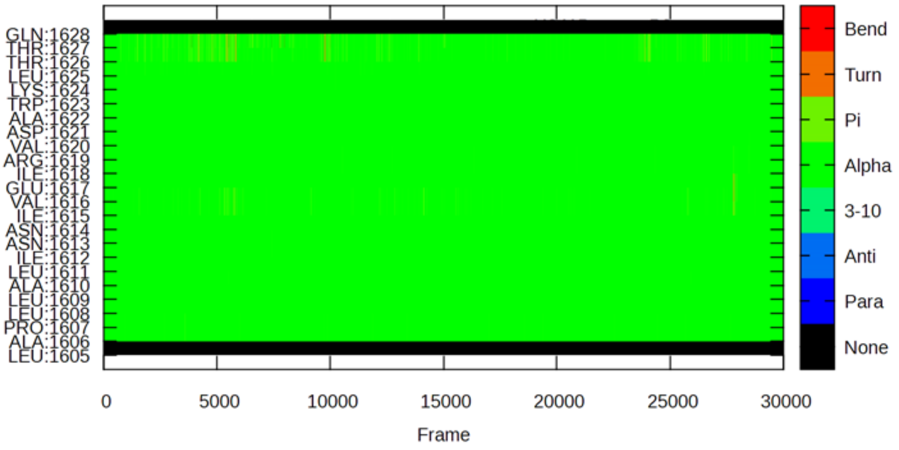

Jarmula, Lusakowska et al.

Supplementary Material 6.

Principal Component Analyses (Essential Dynamics).

The plots shown below present time development of the projections of trajectories along the first eigenvector (PC1) in PCA-1 and PCA-2 plotted against each other in the wild-type ANO5 versus Asp81Gly/Lys132Met/Ser555Ile/Tyr671Cys/Arg758Cys/His841Asp mutant pairs.

ANO5 versus Asp81Gly

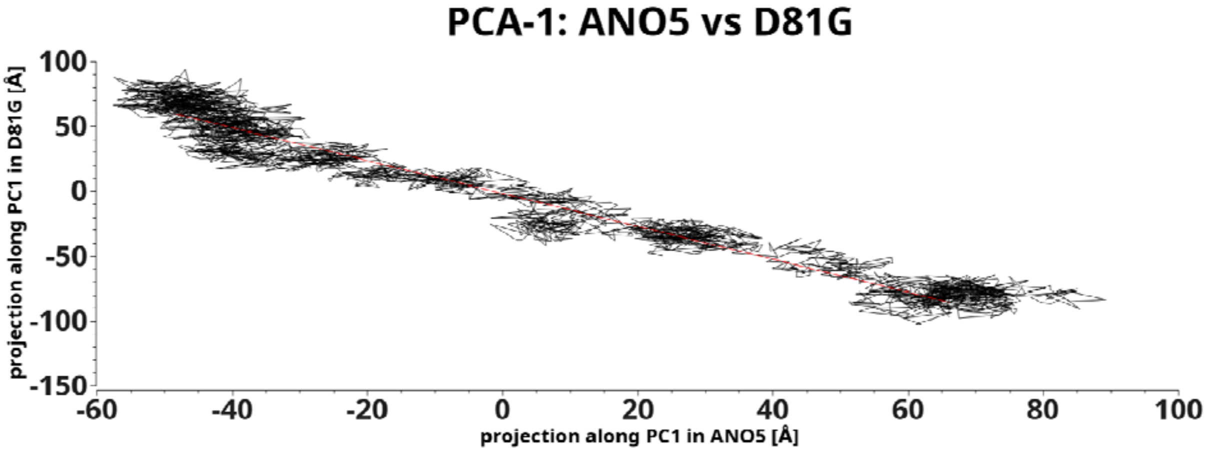

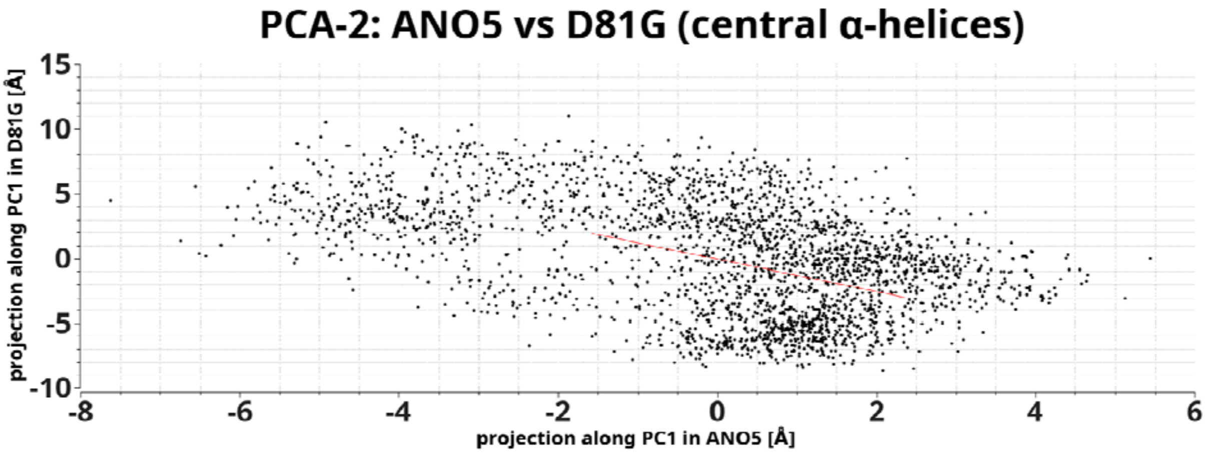

ANO5 versus Lys132Met

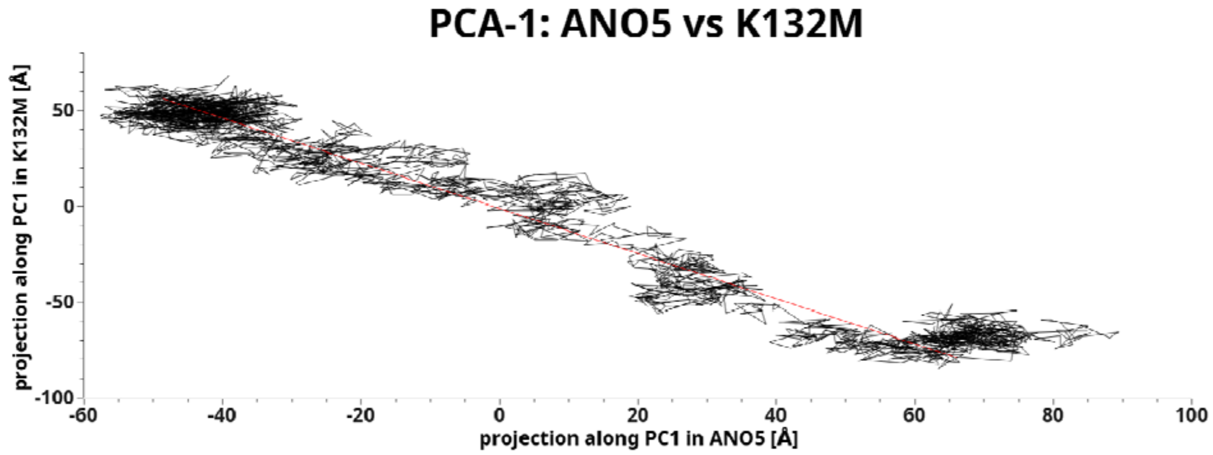

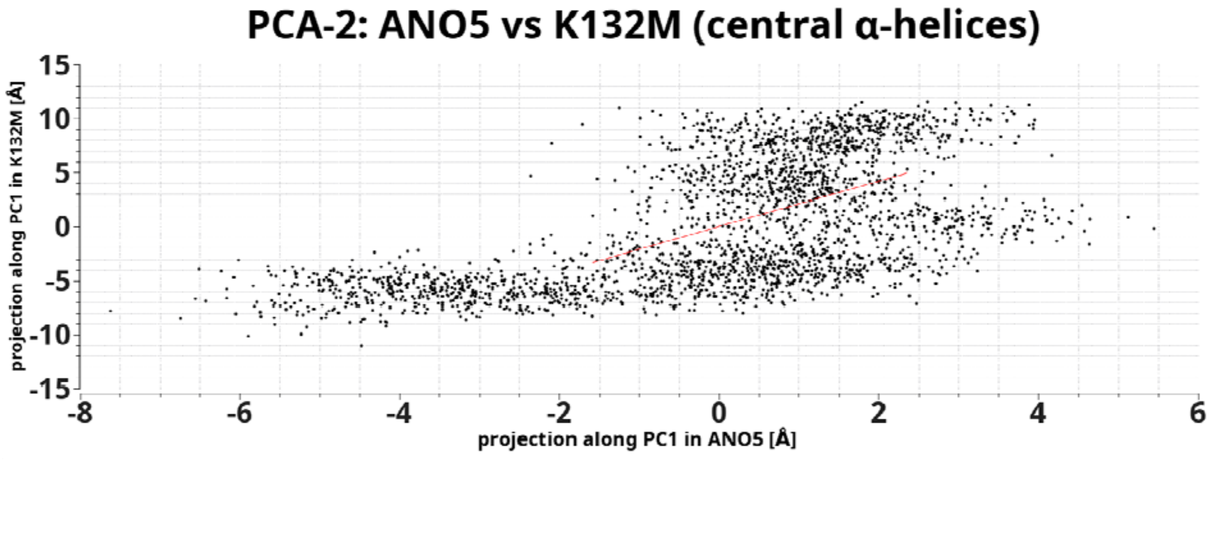

ANO5 versus Ser555Ile

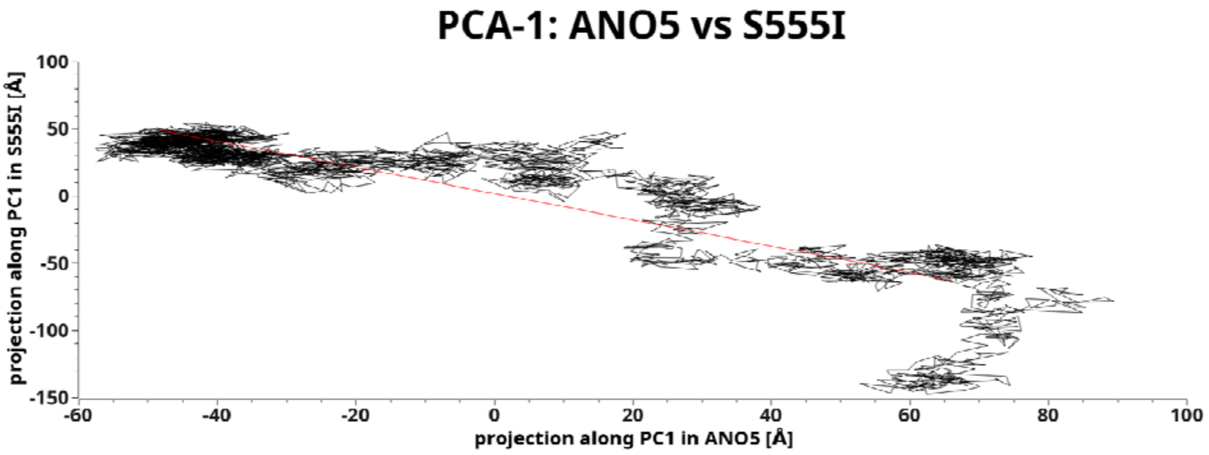

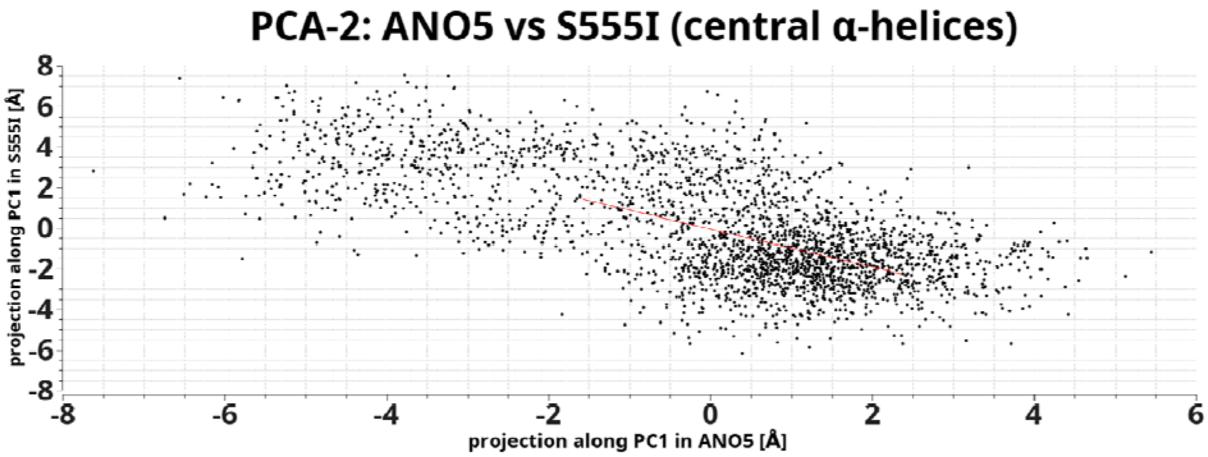

ANO5 versus Tyr671Cys

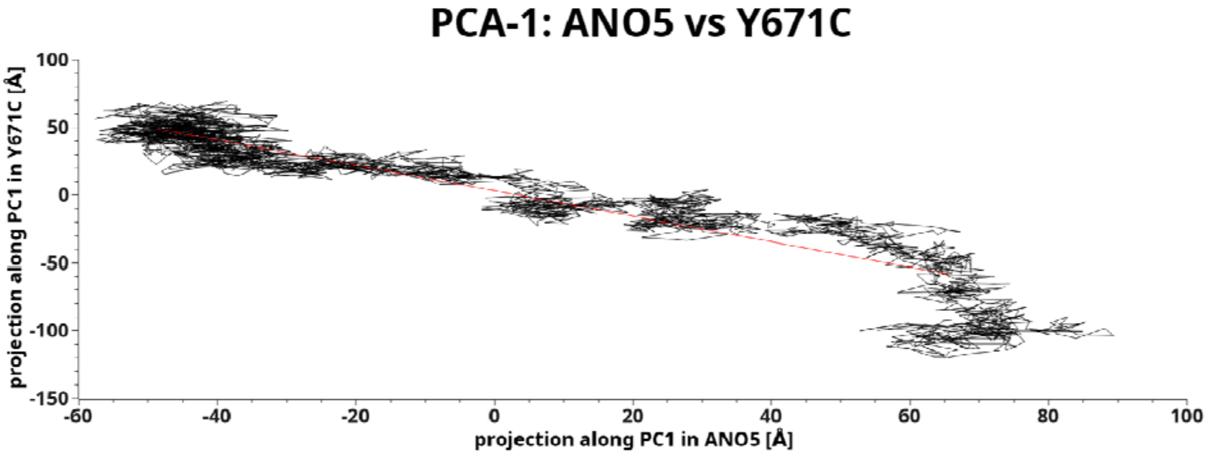

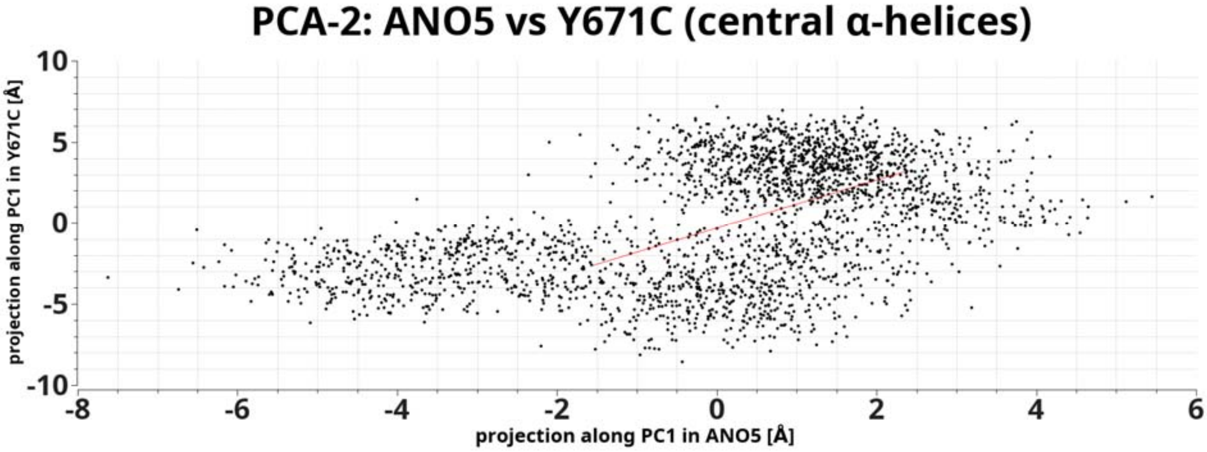

ANO5 versus Arg758Cys

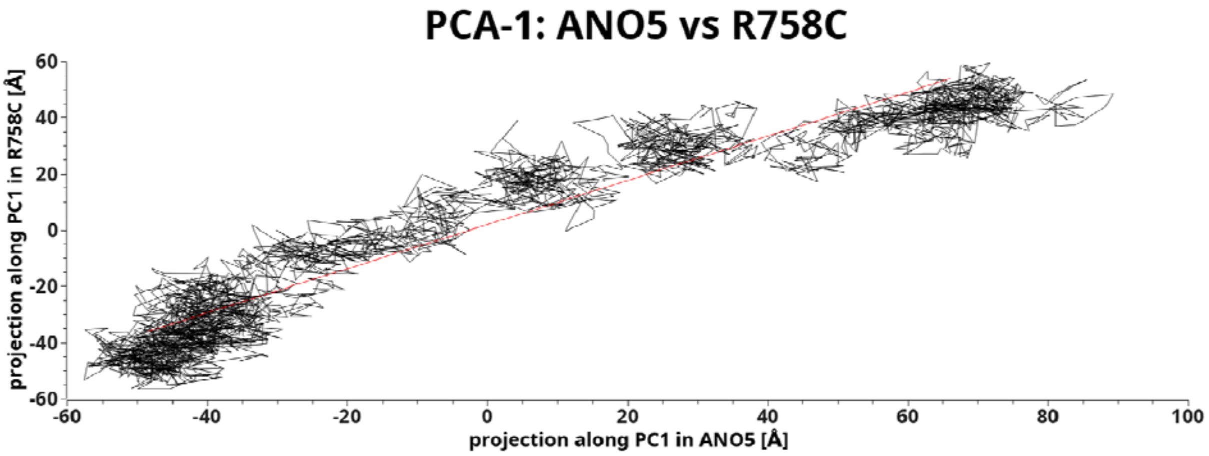

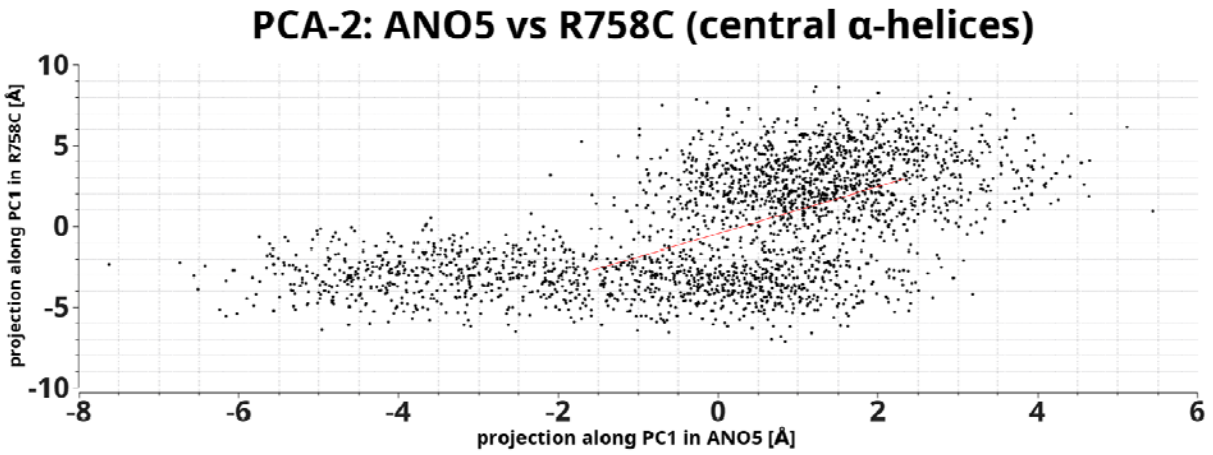

ANO5 versus His841Asp

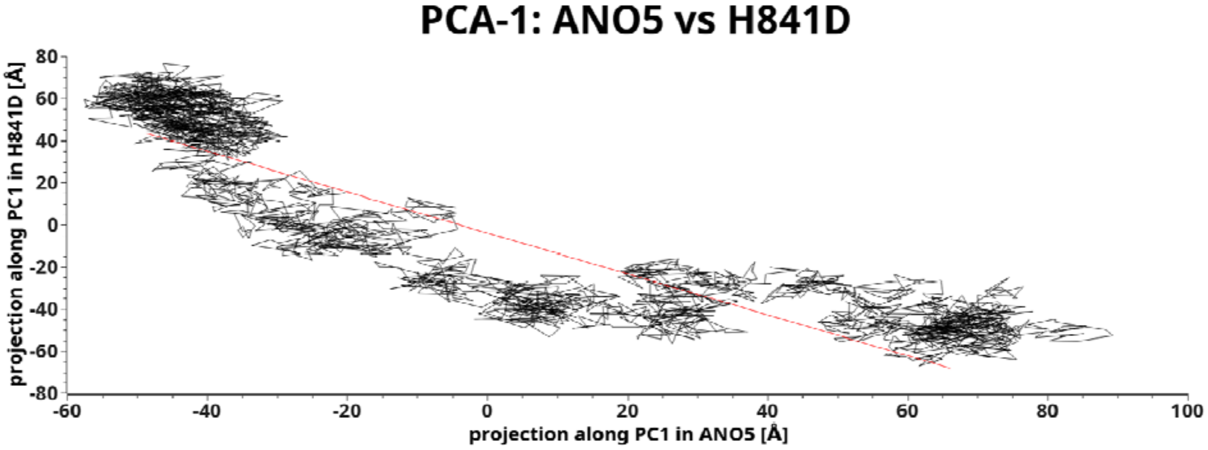

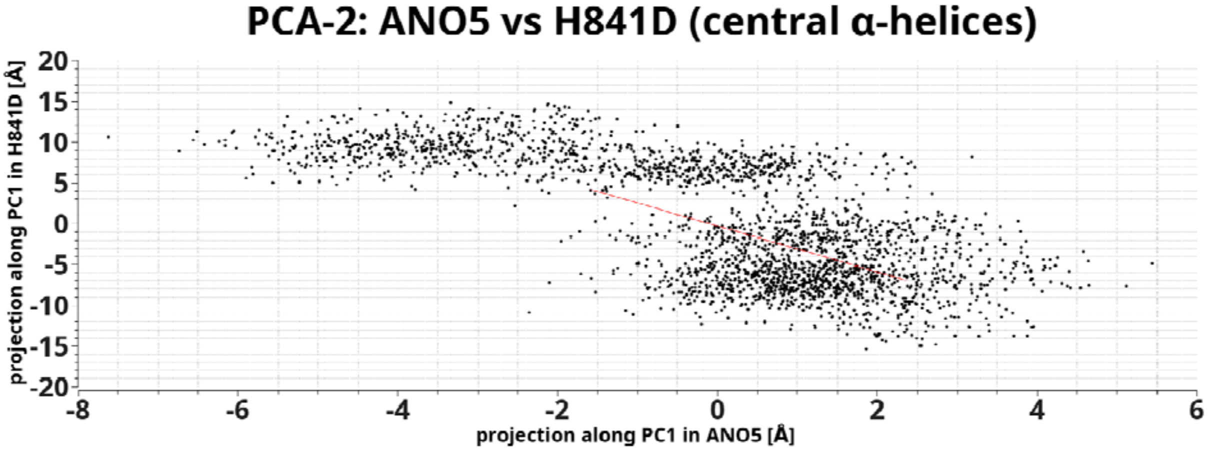

Jarmula, Lusakowska et al.

Supplementary Material 7.

PCA-1 mode-1

The images presented below reflect concerted motions associated with the first eigenvectors (PC1) in PCA-1 for the structures of wild-type ANO5 and its mutants. Chains of subunits A and B are shown as C-alpha tubes and colored red and blue, respectively. The actual frames represent the average structures from the first mode of motion. Motions are represented by green arrows indicating the direction of motion and its amplitude. 1409-1424, the central loop from subunit B.

ANO5

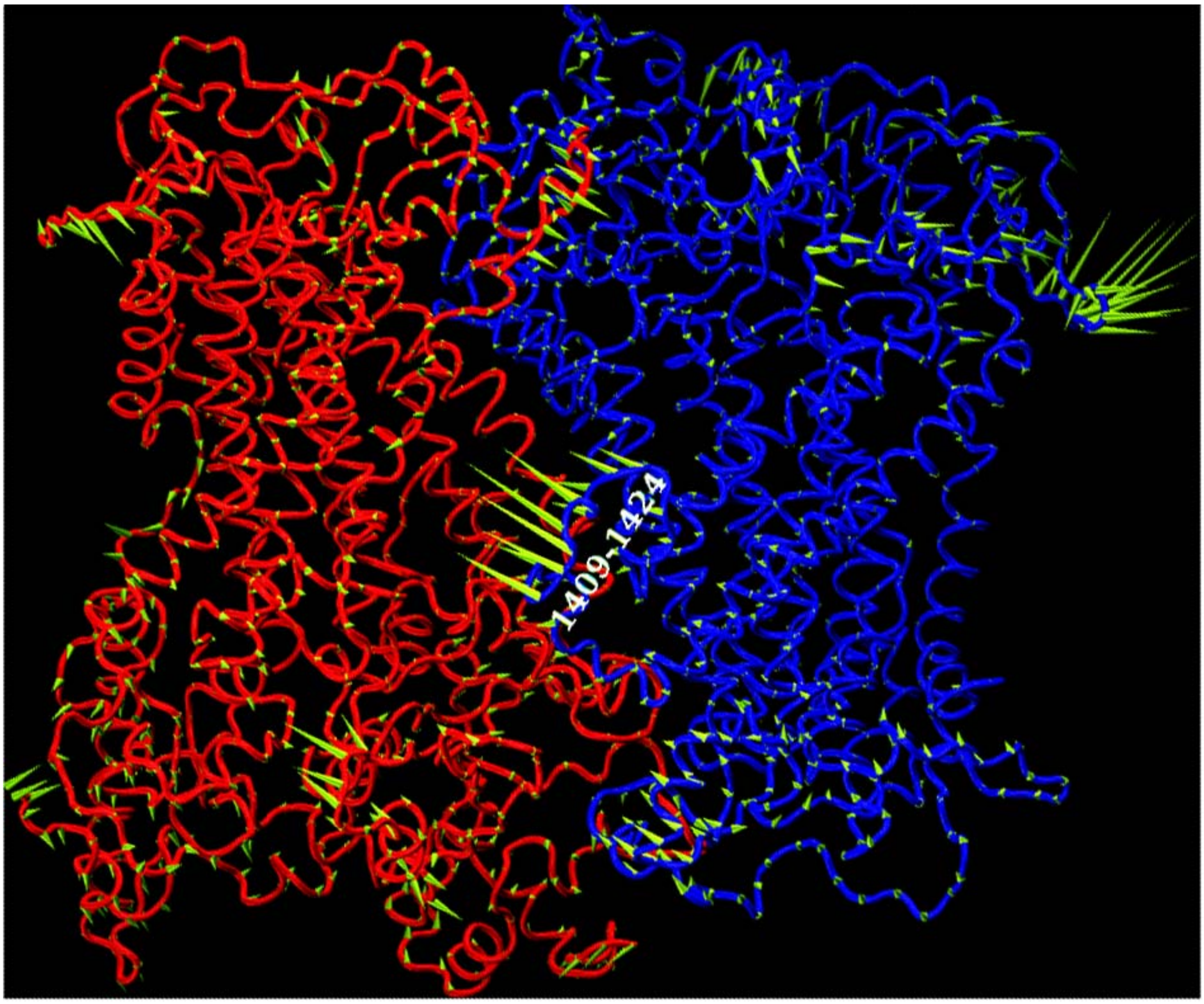

Asp81Gly mutant

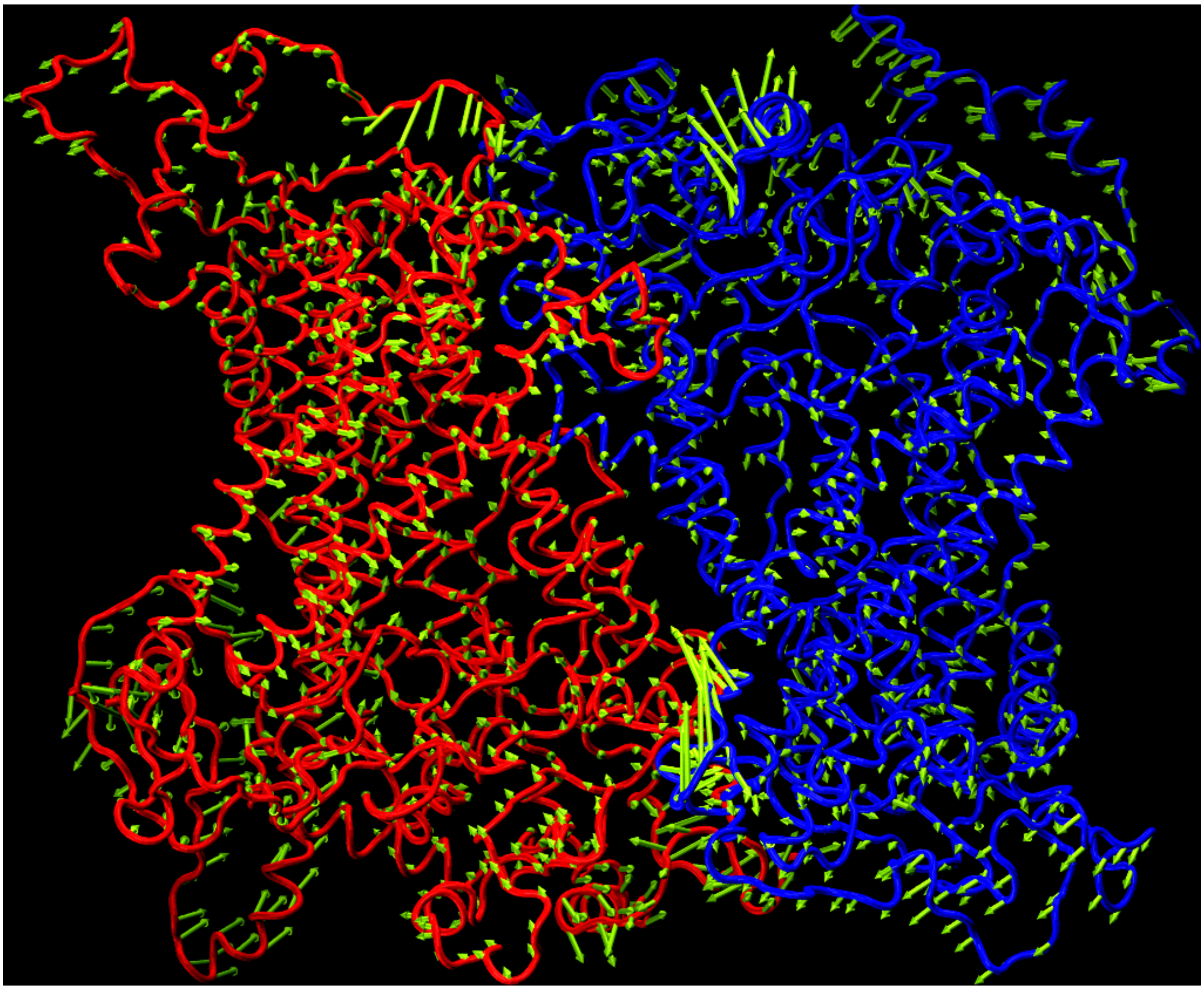

Lys132Met mutant

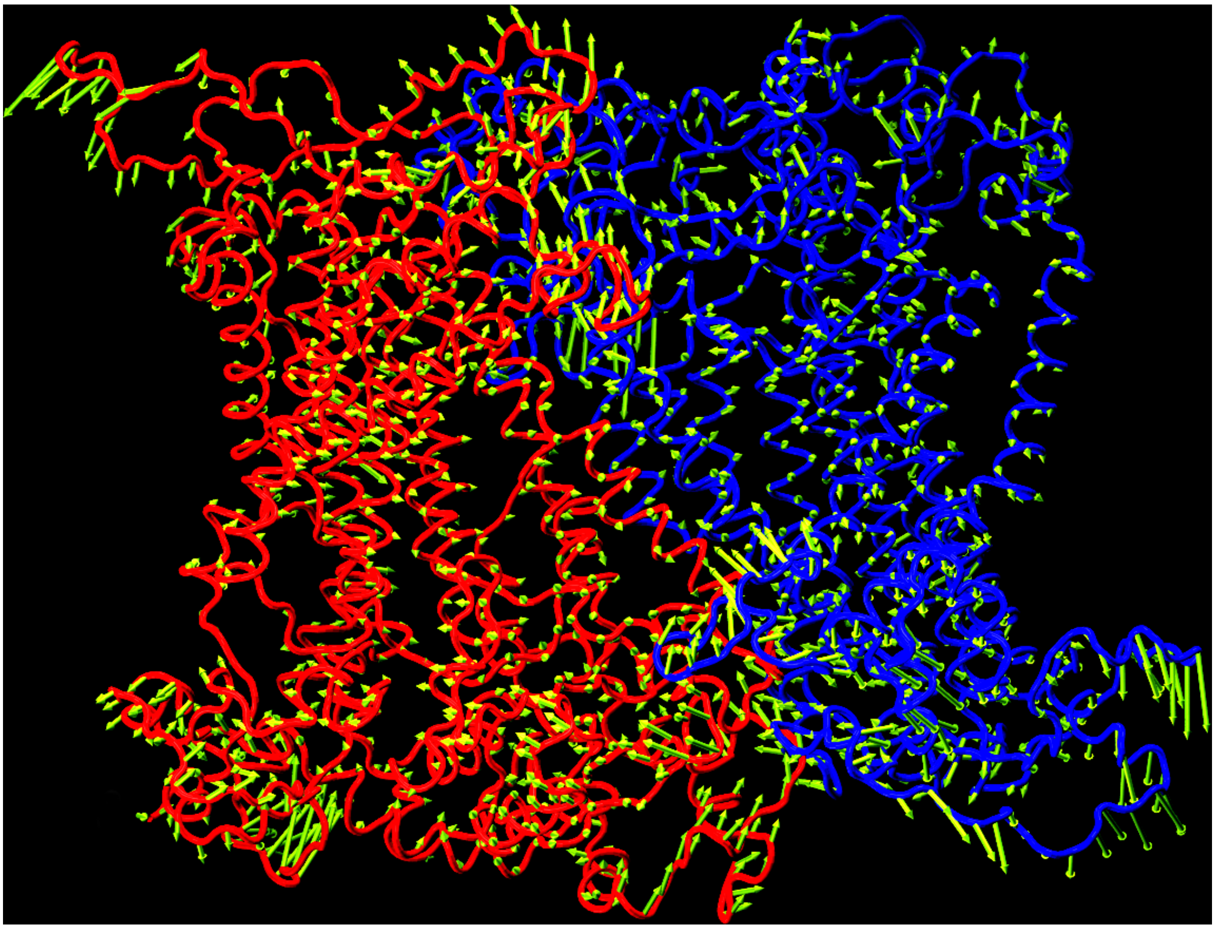

Ser555Ile mutant

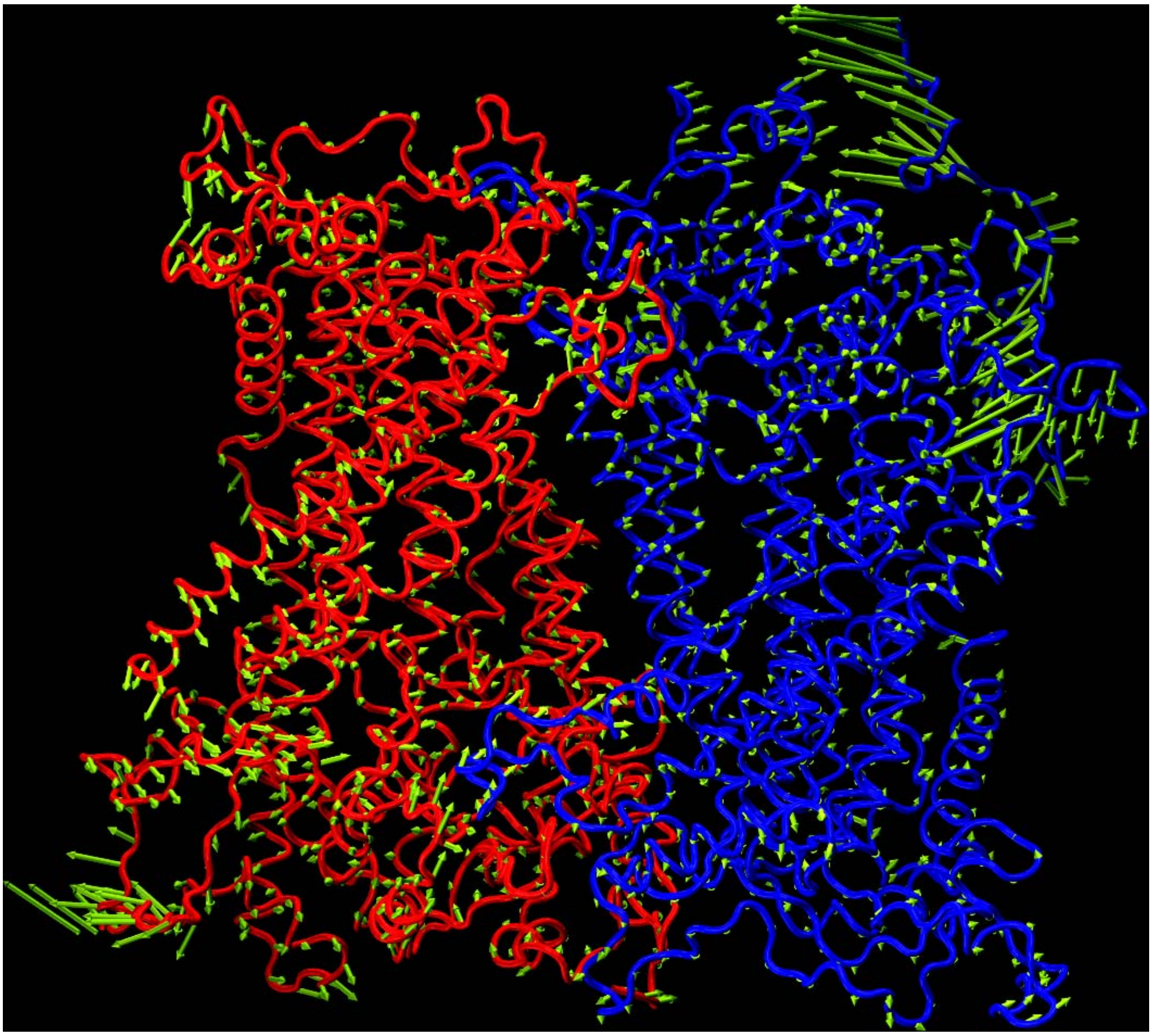

Tyr671Cys mutant

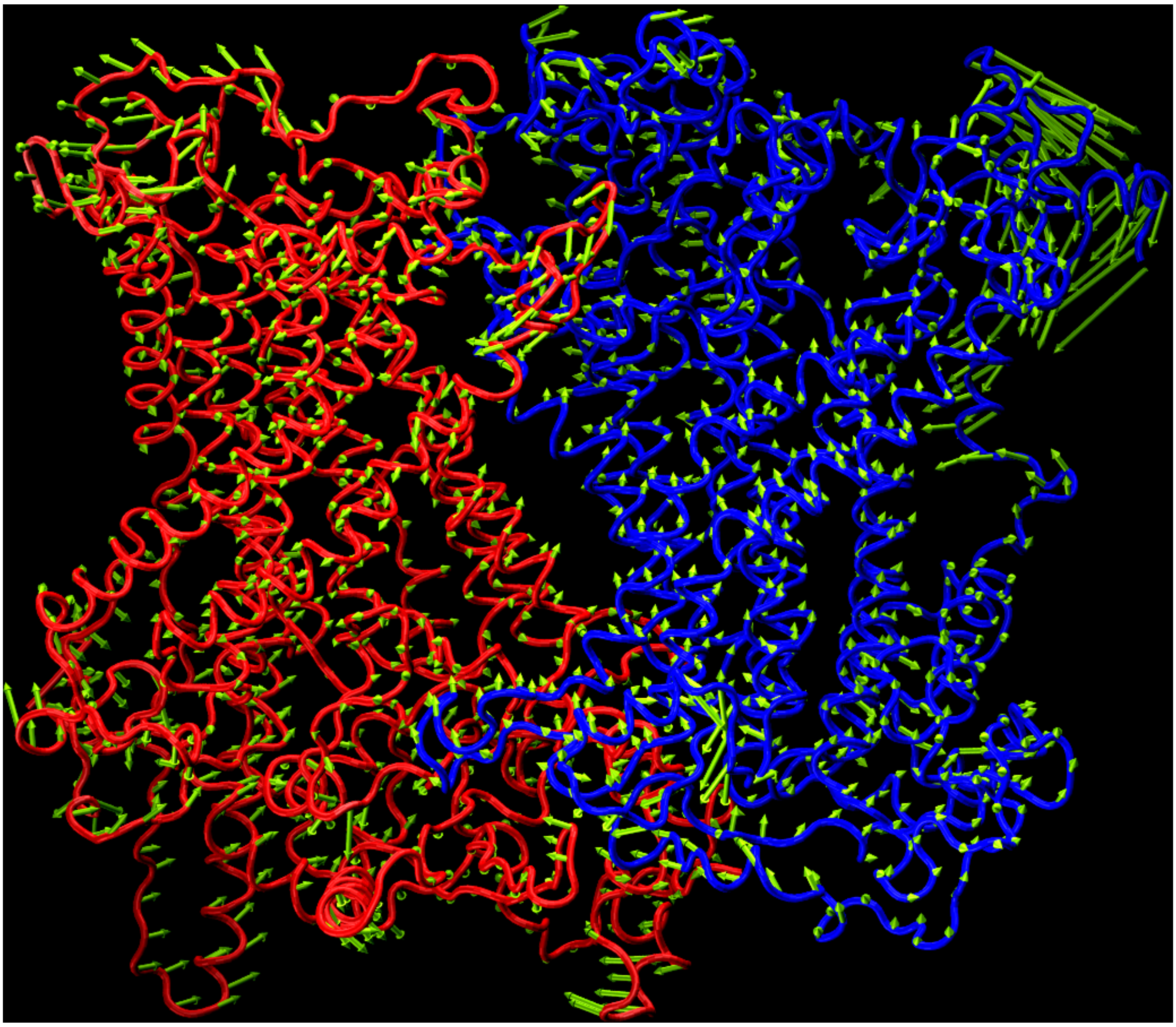

Arg758Cys mutant

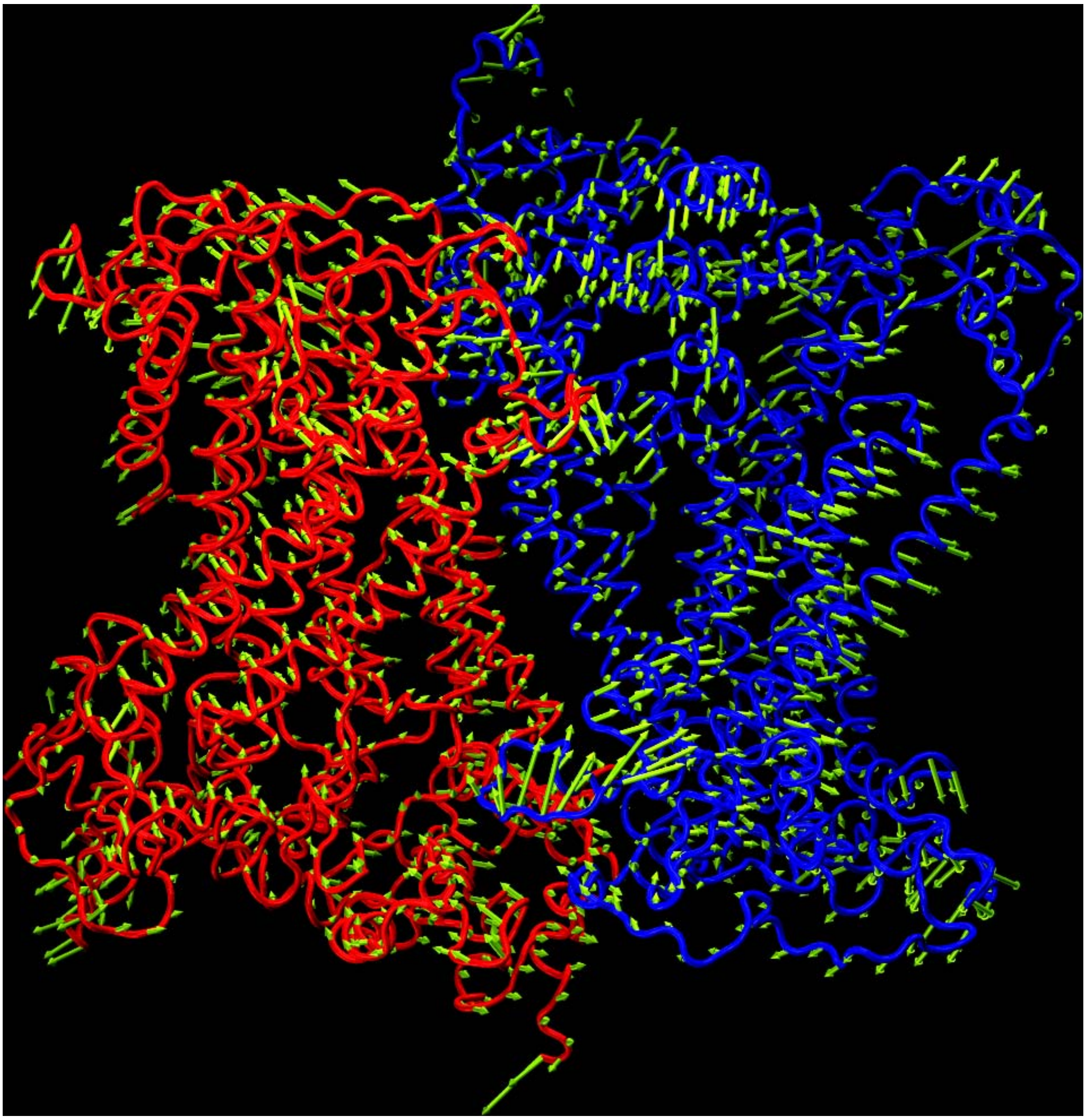

His841Asp mutant

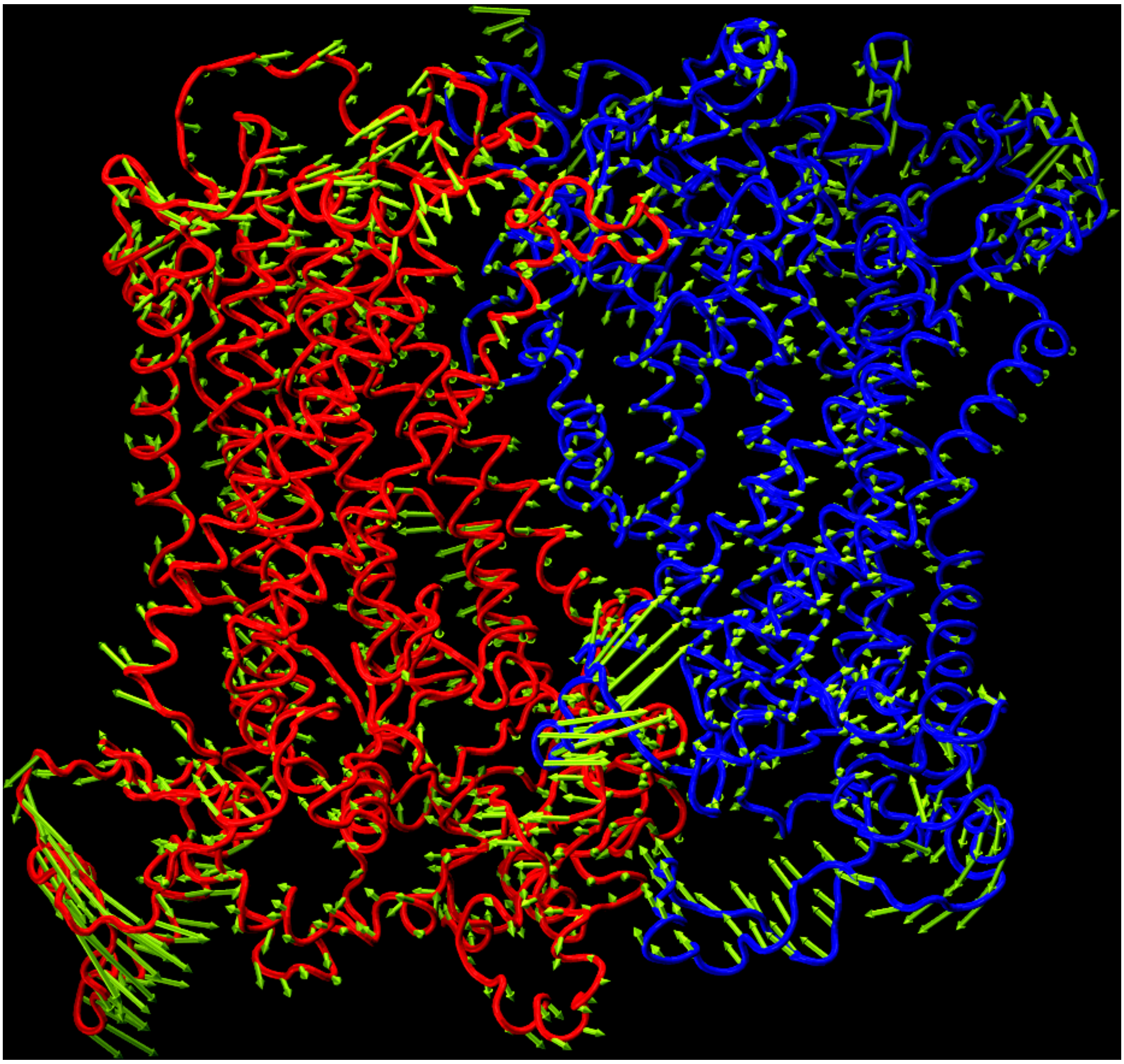

Supplement: Supplementary file 1 — Supplementary Materials 1-7 [file 41598_2019_47849_MOESM1_ESM.pdf]
